# Unlocking the potential of Asian genomic data: a collaborative framework for precision medicine innovation

**DOI:** 10.1093/gigascience/giag052

**Published:** 2026-05-05

**Authors:** The MedHackathon Asia Community, Wasin Poncheewin, Naravut Suvannang, Tazro Ohta, Shih Wee Seow, Yuko Kitano, Afiahayati, Yosuke Kawai, Toshiaki Katayama, Mayumi Kamada, Waritta Sawaengdee, Pundharika Piboonsiri, Scott Comran Edmunds, Sungwon Jeon, Shoichiro Takahashi, Ayu Kasamatsu, Warunyoo Phannasorn, Tanyaluck Kampoun, Wuthiwat Ruangchai, Thavin Bodharamik, Peerut Chienwichai, Viraporn Thepbundit, Tsuyoshi Hachiya, Nitirot Phasitthanaphak, Punna Kunhapan, Piyakrit Wongboonchai, Sunchai Payungporn, Soichi Ogishima, Apiwat Sangphukieo, Thanapak Jaimalai, Pitiporn Noisagul, Jakris Eu-ahsunthornwattana, Kittiya Nittayaboon, Worawich Phornsiricharoenphant, Watcharapot Janpoung, Vorthunju Nakhonsri, Manon Boonbangyang, Surakameth Mahasirimongkol, Natsuko Yamamoto, Pawaris Chanprem, Eiichiro Uchino, Nuttinee Teerakulkittipong, Anupong Joompang, Decha Kumla, Worachote Boonsriwong, Nut Pipatpanyanugoon, Shuhua Xu, Minae Kawashima, Jong Bhak, Nicolas Bertin, Licht Toyo-oka, Mohammed S Mustak

**Keywords:** International data sharing, Asian population genomics, Precision medicine, Regional collaboration, Ethical, legal, and social issues, Federated data analysis, Biobanking

## Abstract

Asian genomic datasets possess unparalleled potential to advance global understanding of human genetic diversity. Encompassing the world’s largest population pool with diverse ethnicities, these datasets capture comprehensive genomic variations shaped by heterogeneous socioeconomic conditions, climate exposures, and clinical environments. However, current national genome initiatives across Asia demonstrate substantial disunity, stemming from limited cross-border communication and collaborative infrastructure, thereby diminishing their collective impact on biomedical research and precision medicine development. The MedHackathon Asia 2025 catalyzed crucial dialogues toward establishing a regional community dedicated to three pillars: harmonized biobank collaboration, standardized genomic data protocols, and cooperative governance frameworks. This multidisciplinary convening brought together researchers, clinicians, bioinformaticians, and national precision medicine program leaders from across Asia to share best practices, identify implementation challenges, and formulate foundational strategies for sustained cooperation. This community review synthesizes critical outcomes from these deliberations, emphasizing the imperative for continuous regional collaboration while advocating for the development of sustainable architectures enabling: (1) equitable biobank resource sharing, (2) genomic data standardization, and (3) ethical governance models. Through consolidation and expansion of this emerging network, Asian nations are expected to lead transformative contributions to global genomic science while ensuring appropriate representation in biomedical innovation. Such coordinated efforts promise to accelerate healthcare advancements with equitable benefits extending throughout the region and worldwide.

## Introduction

Asia is home to over half of the world’s population and harbors immense genetic, cultural, clinical, and climate diversity, presenting an extraordinary opportunity to enhance our understanding of human genetics and the application of next-generation precision medicine. With the growing number of national and international genome projects across the region, easy, open-minded, friendly, and efficient coordination and interaction among Asian researchers can amplify these benefits, facilitating the most impactful biomedical discoveries and clinical advancements in the world. There have been several notable attempts and achievements in the past to address the challenge of integrating diverse Asian populations and building shared genomic resources, such as the HUGO Pan-Asia SNP consortium [1] and the GenomeAsia 100K Project [2]. Moreover, recent advances in AI, particularly language technologies such as neural machine translation, are lowering communication barriers across Asia’s diverse languages and cultures, making cross-border collaboration easier. At the same time, AI-driven analyses in genomics often benefit from large, high-quality, and ancestrally diverse datasets; pooling and harmonizing Asian genomic resources can therefore increase both scientific insight and clinical utility. Together, these trends create a strong opportunity for Asian countries to collaborate and maximize the impact of their unique genomic resources on biotechnology, public health, and patient care.

The challenges inherent in genomic data sharing in Asia span multiple, distinct layers. One major layer concerns data governance and policy: differing privacy protection laws and national data governance policies, together with uneven levels of institutional maturity and financial, technical, and human resources across countries, create substantial ethical, regulatory, and practical barriers that delay genomic data sharing and impede cross-border collaboration [3–6]. Another layer involves technical standardization. The lack of interoperable systems further complicates data sharing, as it becomes difficult to integrate datasets across jurisdictions and institutions. A third layer relates to privacy and security [7, 8]. If high-profile security incidents were to occur, they could erode public trust and prompt more cautious or stricter interpretations of privacy protections and consent requirements, which in turn would further constrain cross-border genomic data sharing. Prior legal scholarship on biobank governance emphasizes that these risks should be addressed through careful governance design from the outset [9]. This includes transparent rules for information management and data access, robust controls for linkage and sharing, and a predefined incident response process.

To overcome these challenges, we propose a region-wide approach to genomic research that prioritizes collaboration, standardization, and continuous knowledge exchange. Establishing a strong network of local researchers across Asia is essential for bridging communication gaps and ensuring that genomic research efforts align with shared goals. By fostering an interconnected research community, we can promote the adoption of common standards for data collection, storage, and sharing, thereby enhancing the impact of national genome projects.

By working together, Asian researchers can build a more inclusive and impactful genomic research ecosystem. A well-coordinated regional approach will not only elevate the value of national efforts but also ensure that Asia contributes meaningfully to global human genetics research. The time to act is now, by fostering stronger collaborations and aligning our research frameworks, we can unlock the full potential of Asia’s genomic diversity for the benefit of all.

MedHackathon Asia was initiated as a crucial step toward realizing this vision. By bringing together experts in biobanking, data analysis, and data governance, this initiative has provided a platform for researchers to discuss current challenges, share experiences, and explore strategies for harmonizing genomic research across the region. This community review summarizes the key discussions from MedHackathon Asia 2025, emphasizing the importance of a continuous and structured effort to strengthen regional collaboration. It highlights the critical need for standardizing genomic data management, addressing regulatory challenges, and establishing a sustainable model for cross-border data sharing.

This paper represents a summary of an ongoing discussion. Situations and policies within individual countries or initiatives may evolve over time; thus, the statements presented here do not represent the official position of any specific country or institution. Instead, they reflect constructive, respectful dialogue among members of our open community.

## Participants of MedHackathon Asia 2025

**Figure 1:**
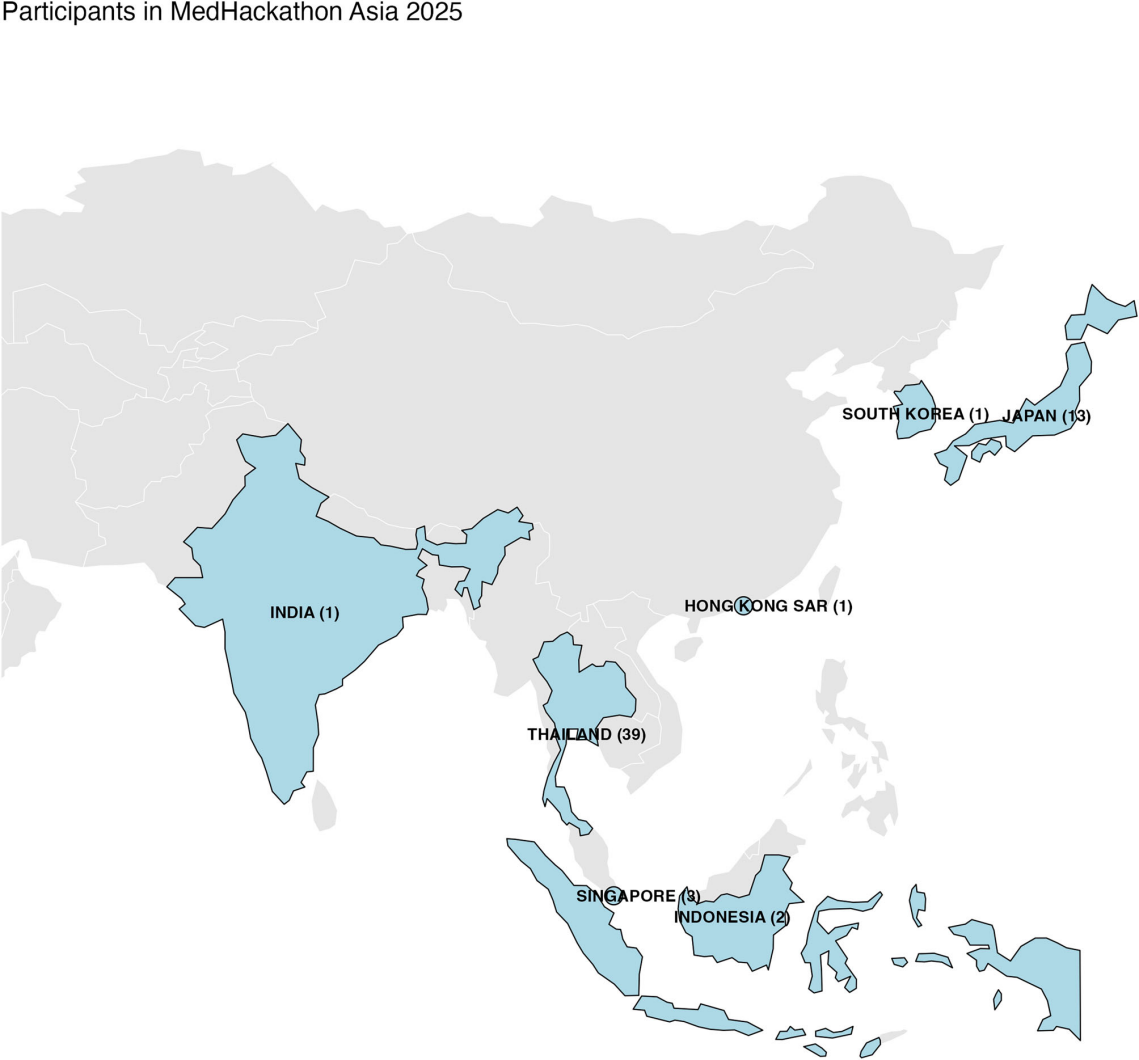
Distribution of participants and bio-resources in MedHackathon Asia 2025. Countries with participants are highlighted, with the number of participants indicated in parentheses.

The geographic distribution of participants in MedHackathon Asia 2025, along with bio-resources across Asia, is illustrated in Fig. 1. Thailand, as the host of this inaugural event, had the highest number of participants, followed by Japan, with other countries contributing smaller numbers. A comprehensive inventory of existing bio-resources reveals that several countries host multiple initiatives, including national biobanks, registries, and alliances. Japan stands out with multiple national biobanks as well as alliance and registry initiatives. Thailand, Singapore, South Korea, India, Indonesia, Hong Kong SAR, Taiwan, China, and Pakistan maintain one or more biobanks, while multinational and Asia-Pacific alliances further highlight regional collaboration in biobanking and data sharing (Table 1; Supplementary Table S1). This visualization underscores the geographic reach of MedHackathon 2025 and the distribution of bio-resource infrastructure across Asia.

**Table 1:** Summary of Asian biobanks. (See Supplementary Table S1 for full details.)

Jurisdiction	Resource name	Participants	Sample type (healthy/disease)	Accessibility	Since
Thailand	Genomics Thailand	50K	Disease/ethnicity	Aggregated data publicly availableControlled local access on request	2019
Japan	Tohoku Medical Megabank project (TMM)	150K	Population	Controlled	2013
Japan	BioBank Japan	270K	Disease	Controlled	2003
Japan	Biobank Network Japan	800K	Disease	Controlled	2018
Japan	JGA (Japanese Genotype-phenotype Archive)	422K (samples)	Population/healthy/disease	Controlled	2013
Singapore	SG10K	10K	Healthy	Managed—access through a DAC	2017
Singapore	PRECISE-SG100K cohort	100K	Healthy	Managed—access through a DAC	2021
South Korea	Korean Genome Project	10K	Population/healthy/disease	Partly open	2015
India	GenomeIndia	≈19K	Population	Supposedly open, but policies still being drafted	2020
Indonesia	Biomedical and Genome Science Initiative (BGSi)	≈4K	Disease	Regulated by Indonesia’s Ministry of Health	2022
Multinational	GenomeAsia 100k	100K (to be enrolled)	Natural populations	Controlled global access on request	2016
Hong Kong*	Hong Kong Genome Project (HKGP)	≈50K	Disease	Local access; global researchers by approval (de-identified data for approved studies)	2021
Taiwan*	Taiwan Biobank (TWB)	≈200K (ongoing)	Population	Global via application (IRB approval and fee required)	2012
Taiwan*	Taiwan Precision Medicine Initiative (TPMI)	≈500K (enrolled; aim 1M)	Various conditions	Open/controlled approved researchers (partner hospitals and Academia Sinica)	2019
Mainland China*	China Kadoorie Biobank (CKB)	510K	Chronic non-communicable diseases (NCDs)	Controlled global access on request (bona fide researchers apply; data delivered on approval)	2004
Mainland China*	Han100K	100K	Natural populations	Web-based, Open	2019
Mainland China*	Chinese Pangenome Consortium (CPC)	≈1K	Natural populations	Web-based, open	2021
Asia-Pacific*	Asian Pangenome Consortium (APC)	100K (to be enrolled)	Natural populations	Web-based, open	2025
Pakistan*	Pakistan Genome Resource	≈145K	Healthy/disease	Controlled by CNCD	2014

Note: “*” indicates there was no official representative from that jurisdiction at MedHackathon Asia 2025.

## The evolving landscape of biobanks in Asia

Biobanks play a pivotal role in biomedical research by providing high-quality biological specimens and comprehensive genomic data, which are essential for studying genetic diversity, understanding disease mechanisms, and advancing personalized medicine. Across Asia, biobank initiatives vary significantly in scale, governance, and accessibility, reflecting different national priorities and regulatory contexts. Japan has been proactive in biobank development since the early 2000s, with BioBank Japan, established in 2003, collecting samples from 270,000 participants and contributing significantly to genome-wide association studies through large-scale SNP array genotyping. Building upon advances in next-generation sequencing, the Tohoku Medical Megabank (TMM) project was launched in 2013 as a pioneering population-based cohort, sequencing whole genomes of 100,000 individuals to establish a foundational genomic reference in Asia. Collectively, these initiatives now form “Biobank Network Japan,” storing >1,680,000 samples from both population-based cohorts and hospital-based biobanks. Additionally, the Japanese Genotype-phenotype Archive (JGA), although not a biobank itself, serves as an essential platform enabling genomic data sharing among researchers domestically and internationally.

At a regional level, the GenomeAsia 100K Project provides an additional cross-country reference resource; its pilot phase reported a whole-genome sequencing (WGS) dataset from 1,739 individuals across 219 population groups and 64 countries in Asia [2]. Singapore’s National Precision Medicine Programme completed its initial phase (2017–21) with 10,000 participants (SG10K) [10], and subsequently concluded Phase II (2021–25) with >100,000 genomes sequenced under the PRECISE-SG100K cohort [11]. The program has since entered Phase III (launched 14 November 2025), which aims to scale national implementation by recruiting up to ∼10% of Singapore’s resident population and integrating genomics into routine healthcare delivery [12]. South Korea’s Korean Genome Project [13, 14] and India’s GenomeIndia [15] initiative are also progressively enhancing their genomic databases, although their access models vary significantly due to different regulatory approaches. Thailand’s Genomics Thailand initiative, launched in 2019, reports ~50,000 genomes across six groups (cancer, rare diseases, non-communicable diseases, infectious diseases, pharmacogenomics, and population genomics/ethnic groups) and emphasizes controlled access to protect data privacy and security [16–18]. In addition, Indonesia’s Biomedical and Genome Science Initiative (BGSi) [19], established in 2022, focuses primarily on disease-specific studies and represents an emerging contribution to the biobanking landscape in the region (Table 1).

In regions without representatives at MedHackathon Asia 2025, several large-scale initiatives continue to shape Asia’s genomic landscape. China has made major investments in biobanking, including the China National GeneBank, established in 2016 in Shenzhen, which serves as a national-level facility for bio-resource conservation, public welfare, and life science innovation. Meanwhile, the China Kadoorie Biobank, initiated in 2004, has enrolled >510,000 participants to investigate chronic diseases across diverse Chinese populations, and the Han100K Initiative systematically catalogs genetic variations across 100,000 Han Chinese individuals, providing a structured genomic resource for China [20]. Hong Kong launched its first large-scale WGS program through the Hong Kong Genome Project, which began in 2021 with the goal of sequencing 45,000–50,000 genomes over 5 years, initially focusing on undiagnosed diseases and hereditary cancers to support the integration of genomics into clinical care [21]. Taiwan has also made significant strides, with the Taiwan Biobank, established in 2012, enrolling >150,000 participants (toward a target of 200,000) and linking genomic data to long-term national health insurance and registry records, enabling robust longitudinal studies [22]. In addition, the Taiwan Precision Medicine Initiative, launched in 2019, is building a 1-million-person, hospital-based cohort through a network of 16 major medical centers, with over half of the target already enrolled and genotyped [23]. Pakistan also hosts an important national-scale resource, the Pakistan Genome Resource, which has been described as a biobank comprising whole-exome and whole-genome sequences of 145,037 participants, with data access handled via academic request and confidentiality agreements [24, 25]. Although China, Hong Kong, and Taiwan were not represented at MedHackathon Asia 2025, their contributions to genomic research are highly valued, and we warmly welcome engagement in future collaborative efforts and regional gatherings (Table 1).

### Data governance and data sharing policies across biobanks

Managing genomic data governance and sample accessibility is a global challenge due to varying privacy laws, data sharing regulations, and national policies [3]. Asian countries similarly encounter these complexities, generally following comparable approaches for domestic data sharing, typically through Data Access Committees (DACs) or equivalent approval processes, as observed in Japan, South Korea, Thailand, and Singapore [4]. However, cross-border genomic data sharing is substantially more challenging and involves additional considerations, notably personal information protection, national security, and economic security concerns. Personal information protection governs privacy and data handling, facilitating easier data exchange among countries with comparable standards, such as GDPR equivalence [26]. In contrast, national economic security concerns, including the classification of genomic data as a potential strategic national resource, introduce additional oversight and restrictions on international sharing [27, 28]. For example, in China, cross-border sharing of genomic data is subject to overlapping legal and administrative controls. Human genetic resource information is regulated alongside genetic materials, and provision of such information to foreign entities may require filing and, where national security or public interest concerns arise, security review [29, 30]. Recent reviews indicate that, despite ASEAN member states having developed individual regulatory arrangements, overlapping legacy frameworks and data sovereignty often impede seamless regional data sharing, underscoring the critical need for consolidated and harmonized governance frameworks [31].

### Country-specific data sharing policies

Asian countries generally adopt a combination of open and controlled access policies depending on data sensitivity. While aggregated statistical data from biobanks are often openly accessible, individual-level data and biological samples typically require ethical approval from committees such as Institutional Review Boards (IRBs) or DACs. For instance, Japan, South Korea, Thailand, and Singapore follow this general model with slight differences: accessing individual-level data internationally generally requires formal research collaboration agreements with local researchers or institutions, as exemplified by Singapore’s managed access approach [7, 32, 33]. Similarly, Thailand’s individual-level data access is currently restricted to domestic researchers, with potential for future expansion. China’s genetic data policies reflect a dual commitment to fostering biomedical innovation and safeguarding national interests. While recent reforms have relaxed some controls, strict oversight, particularly for international collaborations, remains [30].

Researchers must navigate a complex landscape of approvals, ethical reviews, and data localization requirements. Despite these commonalities, understanding precise differences in each country’s policies remains challenging due to varying legal frameworks and cultural contexts, further emphasizing the importance of working toward harmonized governance frameworks. These differences are highlighted when access pathways are compared across three illustrative country examples (Table 2).

**Table 2:** Comparison of access requirements for national genomic resources in Japan, Singapore, and Thailand and as illustrative examples.

Access aspect	Japan	Singapore	Thailand
Primary resource	Tohoku Medical Megabank project, BioBank Japan, and Biobank Network Japan/NBDC Human Database/JGA—WGS + omics + phenotype	PRECISE-SG100K via TRUST platform—integrated WGS + phenotype + EHR for ∼100k participants	Genomics Thailand—national WGS resource (target ∼50,000 Thai participants) covering five priority disease areas and diverse Thai ethnic populations
Domestic access	Permitted controlled access and collaboration with data provider permitted (with review)**•** Japanese academic/clinical and industry researchers apply to use data after approval by DAC and ethics committee	Permitted (via national calls)**•** Singapore public academic/clinical researchers apply to PRECISE calls and TRUST DAC for access to PRECISE-SG100K	Only permitted (Thai institutions)**•** Thai investigators access Genomics Thailand data under national governance and ethics review**•** Data are framed as a national health resource
International access	Currently limited• Meta-analyses have been widely conducted• The Japanese government is currently developing a national economic security framework, and it is not permitted at this time	Allowed (with Singapore PI)• Overseas or industry users must apply with a Singapore research team leading the project• Data are accessed through TRUST, not by downloading	Currently limited• Policies prioritize Thai researchers and domestic health system use• International projects usually work via Thai collaborators who run the analyses inside national systems
Data hosting model	Trusted research environment (TRE) and download• ToMMo supercomputer: on-premises secure analytics with VDI remote access since 2015; the integrated dbTMM dataset is only accessible inside ToMMo supercomputer with DAC approval.• JGA/NBDC: encrypted download of controlled access data to approved institutional or “off-premise” servers is allowed under strict security rules	Trusted Research Environment (TRE)• TRUST platform: cloud-based secure analytics; the integrated PRECISE-SG100K dataset is only accessible inside TRUST with DAC approval• Raw data are generally not downloaded; analysis is done in-platform	Secure data environment (SDE-style)• Genomics Thailand run national systems for WGS processing, variant annotation, and a genome databank, with explicit “security system for data protection”• Individual-level data are kept on national servers; routine download to external sites is not described in public materials
Procedure paperwork and governance	Medium complexity1. Register as an authorized researcher2. Submit proposal for review by DAC after ethical approval3. Sign an MTA/DUA/security agreement; periodic reporting expected	High complexity (managed)1. Apply via PRECISE “Call for Proposals” or collaboration route (2024–26) with Singapore public academic/clinical PI2. Scientific + governance review, including TRUST DAC for EHR-linked data3. Accounts created on TRUST; outputs are checked before export	High complexity (restricted)1. Register as an authorized researcher within Genomics Thailand (Thai institution)2. Submit proposal for review by national governance bodies3. Institutional verification + compliance with Thai data protection rules; strict control on what can leave the secure environment
Key systems	• ToMMo supercomputer—Trusted research environment• dbTMM—integrated database of Tohoku Medical Megabank project• JGA—Japanese Genotype-phenotype Archive (data archive and access)	• TRUST—Trusted Research and Real-world Utilisation and Sharing Tech; national TRE hosting integrated genomic, phenotype and clinical data from PRECISE-SG100K cohort	• ThaiGeR—Thai Genome Reference Database (allele frequencies from >14K Thai genomes).• V@PP—Variant Annotation and Prioritization Platform for clinical/rare disease and cancer use
	• NBDC Human Database—policy and application portal		• Genomics Thailand Supercomputer —national informatics and storage infrastructure.
Possible ideal use case (illustrative)	A collaboration that wants to run analyses on WGS + phenotype data inside a TRE, without moving data out, involving a Japanese-based PI	A collaboration that wants to run analyses on linked WGS + EHR data inside a cloud TRE, without moving data out, led by a Singapore-based PI using the TRUST platform	A Thai-led precision medicine project that analyzes Thai genomes in a secure national environment (e.g., for variant interpretation or clinical filtering), with any international partners receiving summary results, not raw data

To illustrate how governance priorities translate into practical access pathways, Table 2 compares the access requirements for three national genomic resources in Japan, Singapore, and Thailand. Across all three settings, individual-level genomic and linked clinical data are handled under controlled access frameworks, typically requiring institutional ethics approval and review by a DAC (or equivalent), while summary-level outputs are generally easier to share. The main differences emerge for cross-border use and the technical model of access. In Japan, many datasets can be accessed by overseas investigators through JGA/NBDC processes, although permissions are dataset-specific and some resources are restricted to domestic use; access may be provided via encrypted download under strict security requirements or through supervised “data-visiting” arrangements (e.g., ToMMo’s trusted research environment model). Singapore adopts a managed access approach centered on a Trusted Research Environment (TRUST), where international projects typically require a Singapore-based principal investigator (PI) and analyses are performed within the platform rather than through direct raw data transfer. Thailand currently emphasizes domestic health system use, with access largely limited to Thai institutions and international work commonly conducted through Thai collaborators within national secure environments; in practice, external partners typically receive approved outputs or summary results rather than individual-level data. Together, these examples show how policy and infrastructure co-evolve, through platforms to balance scientific utility with privacy, oversight, and data sovereignty considerations.

### Role of digital platforms and infrastructure

Digital platforms play a crucial role in operationalizing data governance and ensuring secure data access and management. The Genomics Thailand initiative employs multiple digital tools, including an SMS platform for real-time updates, a Variant Annotation and Prioritization Platform (V@PP) for genomic analysis, and the Thai Genome Reference Database (ThaiGeR), along with a Secure Data Environment (SDE) featuring an “Air-lock” mechanism to restrict direct data downloads [16, 18, 34, 35]. Additionally, the Thai Exploratory Aggregated Genome Database (ThxAD) further enhances data exploration [36]. Japan’s advanced systems, such as the Tohoku University Tohoku Medical Megabank Organization (ToMMo) Supercomputer and Data Browser, provide multi-tiered access tailored to diverse research needs [37]. Singapore’s TRUST platform offers a managed access, cloud-based environment balancing data accessibility and security [38]. Meanwhile, South Korea’s Korean Genome Project follows an open-access and open-source model [39], while GenomeIndia and Indonesia’s BGSi utilize managed, or hybrid approaches tailored to their regulatory and cultural environments [15].

### Emerging models of consent and ethical considerations

Beyond data access policies and technological solutions, informed consent mechanisms are crucial for ethically sound and sustainable biobanking practices. Emerging models, such as dynamic consent, which allows participants to modify consent preferences in real time, have been explored as potential solutions to address limitations inherent in traditional one-time consent processes [40]. However, dynamic consent faces practical challenges, particularly regarding legacy samples, deceased donors, and long-term participant engagement [41]. As alternatives, approaches emphasizing careful regulation of data usage (“exit control”) or frameworks treating health data as common goods (“data commons”) are gaining attention [6, 42, 43]. The European Health Data Space and ongoing discussions on the ownership and governance of individual-level health data further underscore the importance of ensuring individual autonomy, transparency, and societal sustainability in data sharing practices [43].

### Toward inclusive dialogue and regional harmonization

Addressing these governance challenges will benefit significantly from continued and open dialogue among biobank researchers and genomic scientists across Asia. Forming collaborative communities or advisory boards to facilitate these discussions is a valuable possibility that warrants exploration. However, such efforts must respect each country’s unique policies, guidelines, and cultural contexts. MedHackathon discussions emphasized that all Asian countries should have the opportunity to engage and contribute, ensuring inclusive participation without pressure toward any particular governance model. Ultimately, we believe that mutual understanding, ongoing cooperation, and collaborative development of harmonized policies will be essential for leveraging Asia’s genomic diversity effectively and responsibly in biomedical research.

## Balancing data security and research accessibility

A persistent challenge for biobank management is achieving a balance between robust data security and facilitating meaningful research access. Controlled access repositories, such as Japan’s NBDC Human Database and South Korea’s controlled access genome archive, employ tiered clearance systems based on data sensitivity. DACs are essential in this context, rigorously reviewing and regulating access to ensure that both ethical and scientific standards are met [7].

## Toward a harmonized data governance framework

Given the inherent challenges in cross-border data sharing, the development of an integrated governance framework is imperative. Federated data sharing models, like those advocated by the Global Alliance for Genomics and Health (GA4GH), enable decentralized data analyses without necessitating the transfer of raw data, thereby mitigating many associated risks [44]. In tandem, standardizing metadata formats and data submission guidelines across biobanks can facilitate interoperability and streamline multi-country research initiatives [45]. Ultimately, policy alignment among Asian nations, through the establishment of common ethical guidelines, enhanced institutional cooperation, and proactive policymaker engagement, is crucial for developing a unified framework that not only secures data but also maximizes its scientific utility. Such harmonization is essential for advancing genomic research and improving healthcare outcomes across the region.

## Fostering collaborative genomic innovation

Sharing knowledge and technologies in Asian genomics is emerging as a vital strategy to overcome regional fragmentation and drive collaborative progress in precision medicine. MedHackathon Asia 2025 serves as an excellent initiation of this collaborative approach by uniting diverse experts to standardize methodologies, streamline computational processes, and build shared data resources that capture the region’s rich genetic diversity. The initiatives span pangenome development, federated approaches to cross-border analysis, and regional data catalogues that improve dataset discoverability and coordination, e.g., through federated trusted research environments and shared metadata standards. Collectively, these efforts provide practical mechanisms to strengthen sustained collaboration among Asian countries (Table 3; Supplementary File S2).

**Table 3:** Summary of collaborative initiatives and projects launched at MedHackathon Asia 2025.

Project/initiative	Problem or challenge	Description and approach	Hackathon outcome
Asian Pangenome Initiative	The standard linear reference genome (GRCh38) underrepresents Asian genetic diversity, leading to bias and incomplete variant discovery	A collaborative effort to review sequencing and assembly tools and establish a pangenome graph structure that incorporates multiple Asian genomes for better accuracy	Formalized a multilateral partnership (APC and CPC) on 16 April to coordinate mapping efforts and harmonize ethical guidelines
Asian Genome-Phenome Archive (AGA) Data Catalogue	Genomic datasets from Asia are often difficult to discover or access due to fragmentation and lack of a central registry	Development of a regional catalogue for genomic and phenotypic datasets with standardized metadata to facilitate discovery without centralizing the data itself	Defined a milestone-driven development strategy and a governance model where individual biobanks retain management of their respective repositories
Variant Analysis Pipeline Harmonization	Inconsistent variant calling methods across countries make it difficult to compare or combine results	Cataloging and comparing pipelines (e.g., GATK, DeepVariant) used by different Asian biobanks to develop uniform protocols for genetic interpretation	Documented current pipelines to identify methodological divergences and assess the feasibility of region-wide standardization
CNV Analysis for Clinical Interpretation	Accurate detection of copy number variants (CNVs) is computationally challenging but critical for diagnosing genetic disorders	Integration of breakpoint-based detection and coverage-depth analysis into a single pipeline to improve precision in CNV calling	Initiated the development of a comprehensive analysis pipeline and a clinician-friendly interface to support genetic diagnosis
HPV DNA Detection in PBMC	*Human papillomavirus* (HPV) surveillance is critical for cancer prevention, but population-level data are often limited	A bioinformatics pipeline to detect and quantify HPV DNA traces within unmapped reads of whole-genome sequencing (WGS) data from blood samples (PBMC)	A dedicated team is building pipelines to enable large-scale epidemiological monitoring using existing genomic datasets
Advancing Pharmacogenomics (PGx) and Polygenic Risk Scores (PRS)	Precision medicine tools (PGx, PRS) developed in Western populations often lack accuracy or transferability to Asian ancestries	Creation of Asian-specific genotyping arrays and automated systems for PGx reporting (CPIC guidelines) and PRS calculation tailored to local populations	Established plans for automating clinical reporting and assessing the transferability of PRS models to Asian datasets
Federation of Trusted Research Environments (TREs)	Data privacy laws often prevent raw data from leaving the country, hindering cross-border analysis	Exploring a network of secure, decentralized computing environments (TREs) where analysis travels to the data rather than data traveling to researchers	Identified common security requirements and best practices to support federated architecture
Federated gnomAD Aggregated Variant Browser	Researchers need access to allele frequencies for rare disease studies, but individual-level sharing is restricted	Integrating aggregate allele frequency data from major Asian projects (e.g., PRECISE, TMM) into a federated browser compatible with gnomAD	Established as a low-risk “first step” for cross-border sharing that avoids transferring sensitive individual data
Imputation Server for Thai and Asian Genomes	Lack of population-specific reference panels reduces the accuracy of genotype imputation for Asian populations	Developing a standardized imputation pipeline using a Thai-specific reference panel (WGS + HLA), utilizing common workflow language (CWL)	Defined the roadmap for constructing the panel, validating it, and launching a secure web-based imputation server
ELSI in Genomic Data Sharing	Varying legal definitions of “personal information” and cultural trust issues create barriers to international collaboration	Examination of Ethical, Legal, and Social Issues (ELSI) to create frameworks for equitable benefit-sharing and legal compliance across jurisdictions	Initiated discussions to align ethical frameworks and ensure responsible data governance

The region faces an urgent need for common data standards. The Asian Pangenome Initiative undertakes a detailed review of human pangenome studies across Asia by evaluating sequencing technologies, assessing genome assembly quality, and identifying methodological gaps. This effort informs the development of uniform data standards that support robust comparative research across diverse Asian populations.

**Figure 2:**
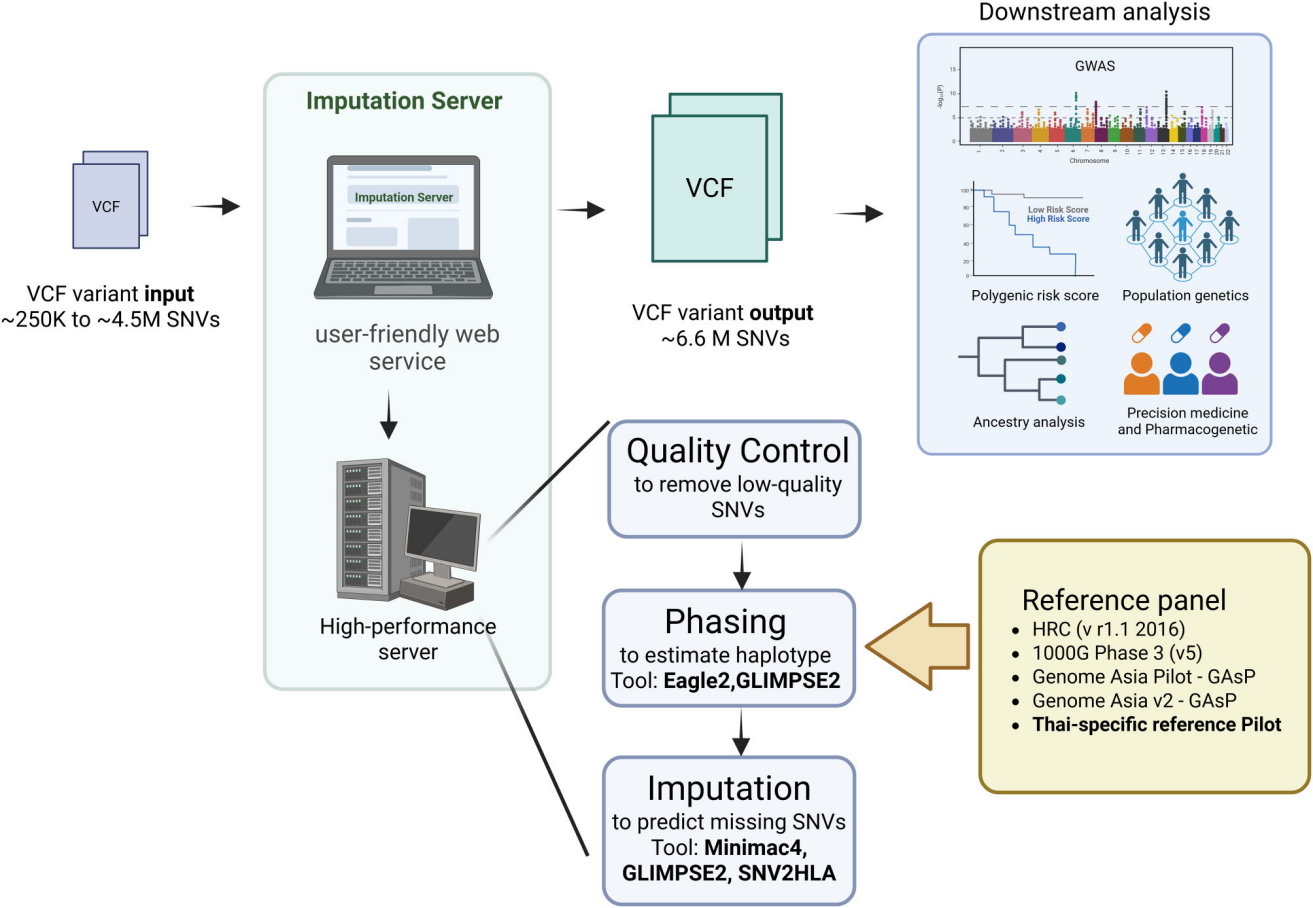
The concept of the imputation server with the Asian and Thai reference panel. The figure illustrates a standardized genotype imputation workflow tailored to Asian populations, incorporating high-quality reference panels such as HRC (v1.1), 1000 Genomes Project Phase 3, Genome Asia Pilot (GAsP), and a Thai-specific reference pilot. The imputation process begins with user-submitted VCF files (~250K to 4.5M SNVs), processed through a user-friendly web service hosted on a high-performance computing server. Uploaded variants undergo quality control to remove low-quality SNVs, followed by haplotype phasing using tools such as Eagle2 and GLIMPSE2, and imputation to predict missing SNVs using Minimac4, GLIMPSE2, or SNV2HLA. The resulting VCF file (∼6.6M SNVs) supports downstream analyses, including genome-wide association studies, polygenic risk scoring, ancestry analyses, precision medicine, and population genetics research. This standardized approach promotes harmonized genomic analyses across Asian populations, facilitating regional collaboration and improved accuracy in genetic interpretations.

At the same time, significant projects are working to harmonize computational pipelines for genomic analysis. The Variant Analysis Pipeline Harmonization project catalogs and compares tools used for variant calling to develop uniform protocols for genetic interpretation. In addition, the CNV Analysis for Clinical Interpretation project is developing a comprehensive pipeline that integrates methods for detecting copy number variants using both breakpoint detection and coverage-depth approaches. A dedicated team is also developing a pipeline for the HPV DNA Detection in PBMC WGS Data project to detect and quantify traces of human papillomavirus DNA from WGS data of peripheral blood mononuclear cells. The Imputation Pipeline/Server for Thai and Asian genome project aligns with the imputation workflow that incorporates Japanese reference panels [32] and is implemented using the Common Workflow Language (CWL) [46]. This initiative further contributes by establishing a standardized genotype imputation framework that is tailored to the Asian and Thai population (Fig. 2).

**Figure 3:**
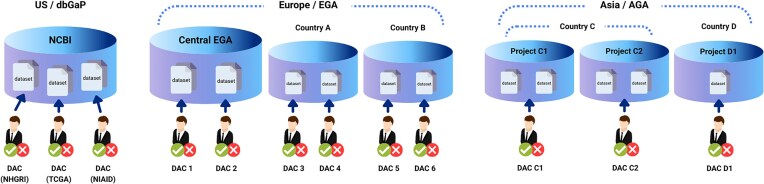
Comparison of data repository structures: dbGaP (USA), EGA (Europe), and the proposed Asian Genome-Phenome Archive (AGA). This figure illustrates conceptual differences in governance and data access among genomic data repositories in the United States (dbGaP), Europe (EGA), and the proposed Asian Genome-Phenome Archive (AGA). In the USA, a centralized repository is managed by the National Center for Biotechnology Information (NCBI), and Data Access Committees (DACs) from individual NIH institutes (e.g., NHGRI, TCGA, NIAID) control access to the data they fund. Europe’s EGA is similarly centralized under the European Bioinformatics Institute (EBI), but individual countries may also host their own federated EGA instances, with data submitters designating which DAC controls their datasets, often forming their own committees. In contrast, the proposed AGA model in Asia suggests that each genomic research project or biobank independently manages its repository and DAC, providing permissions to all datasets within their repositories. Additionally, while dbGaP and EGA typically manage biological specimens independently from digital data (metadata, nucleotide sequences), the AGA allows biobanks themselves to archive both digital data and biological specimens in an integrated manner.

Interoperability and robust metadata management are critical to promoting cross-border collaboration. The Asian Genome-Phenome Archive is a project that seeks to build a comprehensive regional catalogue of genomic and phenotypic datasets, complete with detailed metadata and clearly defined contact points (Fig. 3). Complementing this effort is the Federated gnomAD Aggregated Variant Browser, which aims to integrate allele frequency data from multiple Asian projects to improve data accessibility. Another initiative focuses on establishing a Federation of Trusted Research Environments to provide secure and decentralized platforms for data sharing while ensuring compliance with legal requirements across countries.

Capacity building and training are essential to ensure that technological advancements are matched by a skilled workforce. The Advancing Pharmacogenomics and Polygenic Risk Scores for Precision Medicine project is developing a genotyping array tailored to Asian populations and automating the reporting of pharmacogenomic profiles and polygenic risk scores. This initiative not only standardizes clinical and analytical frameworks but also offers hands-on training opportunities for clinicians, bioinformaticians, and researchers. Such capacity building is crucial for integrating genomic medicine into routine clinical practice and for supporting long-term advancements in precision medicine.

MedHackathon Asia 2025 provides a dynamic platform for sharing knowledge and technologies across Asia. By facilitating collaborative projects that address common challenges and promote innovative solutions, the event lays the groundwork for a more integrated and impactful genomic research ecosystem throughout the region (Supplementary File S2).

## Future perspectives

**Figure 4:**
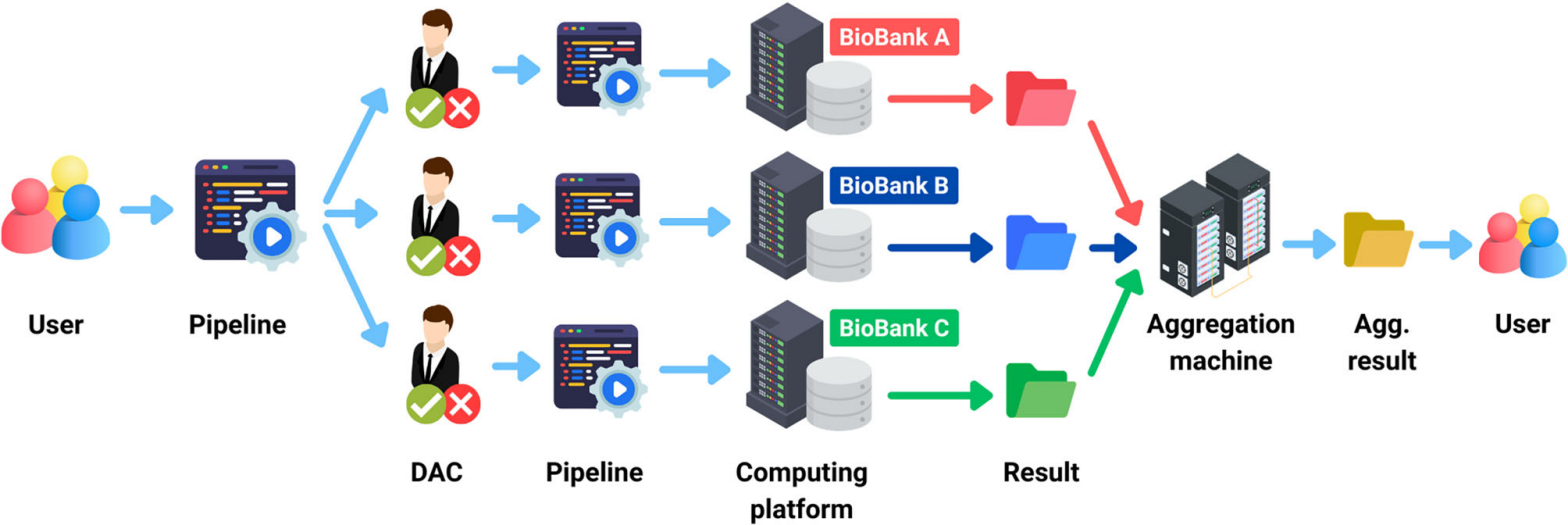
Proposed framework on data sharing across Asian countries. The figure outlines a conceptual framework for secure and decentralized genomic data sharing among biobanks and research institutions across Asia. Under this model, data analysis workflows or pipelines initiated by users undergo approval by each institution’s Data Access Committee (DAC). Upon approval, the workflows are executed on secure computing platforms managed individually by each biobank, ensuring data privacy and regulatory compliance. Results from multiple biobanks are then aggregated securely, enabling the users to access combined analytical outcomes without direct access to raw data. This federated approach facilitates collaborative research across Asian countries while fully respecting each country’s ethical guidelines, regulatory requirements, and data sovereignty.

The vision for genomic research in Asia depends on the successful implementation of standardized data sharing protocols and the development of an integrated data ecosystem. In a future where centralized guidelines and federated data sharing models are widely adopted, Asian institutions would follow uniform procedures for data collection, submission, and metadata annotation. Such standardization would facilitate secure, decentralized analyses while upholding local ethical and regulatory requirements (Fig. 4). MedHackathon Asia 2025 has already initiated this collaborative process by inspiring prototype concepts, such as a unified pangenome framework and a Thai-specific imputation server, which, although still in the conceptual stage, offer promising case studies for validating these protocols.

However, to sustain the technical collaboration among institutes, it is necessary to revive or launch a new coordination hub that is bottom-up and objective-driven by participating researchers. Conferences and even Asian researcher-initiated journals will be necessary to educate the next generation of genomics researchers with a steady ecosystem of paper publication and data sharing in the region. The key problem of failing to have such an Asian control committee or organization is that the ever-changing political environment of all the nations keeps disrupting science-driven guidelines, funding, and policies. Once such a non-government-initiated, bottom-up virtual organization is established, with modest but stable support from governments across the region, it could strengthen coordination among biobanks and genome projects and support their application to other areas of biomedicine. This will enable Asian researchers to efficiently cope with international organizations such as GA4GH and even lead the biobank resources and future precision projects of the world. As AI and distributed information exchange systems such as blockchain are advancing fast, Asia’s enormous body of diversified data and human resources can play a pivotal role in human health and longevity.

Looking forward, if maximum data sharing across Asian genomic initiatives with a proper research-driven coordination hub is achieved, the impact on both research and clinical practice for the world could be profound. An integrated data ecosystem that incorporates diverse genomic and phenotypic datasets would empower researchers to perform large-scale meta-analyses, uncover population-specific genetic insights, and develop innovative computational approaches to disease prediction and personalized treatment. This comprehensive data sharing would, in turn, support transformative advances in precision medicine and public health.

The potential applications of such an open data environment are multifaceted. In the field of population genetics and evolutionary studies, the vast genetic diversity of Asia, shaped by centuries of migration, adaptation, and environmental influences, could be analyzed in unprecedented detail. Researchers would be able to elucidate the genetic structure of diverse ethnic groups, trace historical migration patterns, and identify adaptations unique to specific populations. This inclusive approach would correct the current bias toward Western-centric datasets and ensure that underrepresented groups contribute to and benefit from global genomic research.

Moreover, enhanced data sharing would facilitate precision medicine tailored to Asian populations. By leveraging shared data, researchers could identify genetic risk factors and develop polygenic risk scores that more accurately reflect the genetic architecture of diseases prevalent in Asia, such as hepatocellular carcinoma, nasopharyngeal cancer, and thalassemia. The pooling of data from multiple countries would also enable more effective studies of rare diseases, where limited sample sizes in individual nations have historically constrained research efforts.

In the realm of public health and epidemiology, a fully integrated open and transparent data framework will enable real-time genomic surveillance to track infectious diseases and monitor viral evolution. By linking genomic data with clinical and epidemiological information, it would be possible to identify genetic markers associated with disease severity or vaccine response, thereby informing targeted public health interventions.

MedHackathon Asia 2025 has thus established a promising initial foundation for collaborative data sharing in the region. With the adoption of standardized protocols and the realization of a comprehensive, secure data ecosystem, the collective sharing of genomic resources can substantially accelerate scientific discovery and revolutionize precision medicine across Asia.

## Additional files

**Supplementary Table S1:** Summary of the biobanks. An overview of major biobanks and genomic resources across Asian jurisdictions, tabulating for each resource the jurisdiction, resource type, resource name, approximate number of participants, sample type (healthy/disease), sequencing platform, secure data platform, accessibility (local/global, free/requested), metadata, year of establishment, URLs and contact information, data browser, policy URL, and Data Access Committee (DAC) contacts.

**Supplementary File S2:** Project descriptions. Detailed descriptions of the projects initiated during MedHackathon Asia 2025, including: the Asian Pangenome Initiative; Asian Genome-Phenome Archive (AGA) Data Catalogue; Variant Analysis Pipeline Harmonization; CNV Analysis from Paired-End Sequence for Clinical Interpretation; HPV DNA Detection in PBMC WGS Data; Advancing Pharmacogenomics (PGx) and Polygenic Risk Scores (PRS) for Precision Medicine; Federation of Trusted Research Environments (TREs); Ethical, Legal, and Social Issues (ELSI) in Genomic Data Sharing; Federated gnomAD Aggregated Variant Browser; Imputation Pipeline/Server for Thai and Asian Genomes; and Imputation Server incorporating Japanese Haplotype References.

## Abbreviations

BGSi, Biomedical and Genome Science Initiative; CWL: Common Workflow Language; DAC: Data Access Committee; GA4GH: Global Alliance for Genomics and Health; IRB: Institutional Review Board; JGA: Japanese Genotype-phenotype Archive; TMM: Tohoku Medical Megabank; V@PP: Variant Annotation and Prioritization Platform; WGS: whole-genome sequencing.

## Competing interests

Scott C. Edmunds was employed by GigaScience Press/BGI Group at the time of the first submission of the manuscript. Sungwon Jeon is the CEO of AgingLab and Geromics, Inc. and is employed by Clinomics, Inc. Jong Bhak is a founder of AgingLab. Shoichiro Takahashi is an employee of Trinet Corporation. Tsuyoshi Hachiya is the CEO of Genome Analytics Japan Inc. All other authors declare that they have no potential competing interests.

## Supplementary Material

giag052_Supplemental_Files

giag052_Authors_Response_To_Reviewer_Comments_original_submission

giag052_GIGA-D-25-00441_original_submission

giag052_GIGA-D-25-00441_revision_1

giag052_Reviewer_1_Report_original_submission Reviewer 1 -- 11/19/2025

giag052_Reviewer_1_Report_revision_1 Reviewer 1 -- 4/6/2026

giag052_Reviewer_2_Report_original_submission Reviewer 2 -- 12/4/2025

## Data Availability

Not applicable.
