# Supplementary material for: Unlocking the potential of Asian genomic data: a collaborative framework for precision medicine innovation
Source: Gigascience. 2026 May 5;15:giag052. doi: 10.1093/gigascience/giag052 (PMC13196594; doi:10.1093/gigascience/giag052)
Supplement: giag052_Supplemental_Files [file giag052_supplemental_files.zip › Supplementary File S2 - project descriptions.docx]

## **Supplementary File S2 - Project descriptions**

During the MedHackathon 2025, a variety of projects were initiated, each focusing on the utilization of genomic and phenotypic resources within Asia. While these projects span a wide range of themes, from data sharing to genomic analysis tools, they all share a common goal: to advance scientific research while respecting the rights and sovereignty of data-owner countries and their populations. The collaborative efforts emphasize ethical data use, ensuring that the benefits of shared resources are maximized for the advancement of precision medicine and public health, while maintaining trust and transparency among all participants.

###

### Asian Pangenome Initiative

This project explored the current landscape of human pangenome studies in Asia, examining sequencing approaches, genome assembly quality, and analytical tools used across various studies. Traditional genomics often relies on a single linear reference genome, such as GRCh38, which underrepresents the vast genetic diversity of Asian populations, leading to reference bias and incomplete variant discovery. In contrast, pangenome graph approaches incorporate multiple genomes into a unified structure, allowing for more accurate alignment, improved detection of structural variants, and better representation of population-specific alleles. By identifying current gaps and challenges, this initiative aims to advance pangenome research in Asia and promote a more inclusive understanding of regional genomic diversity. On April 16, representatives from China (Shanghai and Taipei), Japan, India, Vietnam, Malaysia, Thailand, the Philippines, Sri Lanka, Pakistan, and Bangladesh convened to formalize a multilateral partnership agreement between the Asian Pangenome Consortium (APC) and the Chinese Pangenome Consortium (CPC). This landmark collaboration establishes a framework for coordinated pangenome mapping efforts, leveraging complementary resources and expertise to address regional genomic diversity. The consortium aims to catalyze discoveries in population-specific disease mechanisms while advancing therapeutic development through three core initiatives: (1) establishing shared analytical frameworks, (2) harmonizing ethical guidelines for data sovereignty, and (3) developing ancestral-aware clinical interpretation pipelines.

###

### Asian Genome-Phenome Archive Data Catalogue

The Asian Genome-Phenome Archive (AGA) aims to establish a regional platform for improving access to genomic and phenotypic data from Asian populations. To address the underrepresentation of Asian populations in global genomic databases, the project focuses on building a data catalogue that facilitates dataset discovery and access across different countries. During the hackathon, participants worked on drafting a milestone-driven strategy to guide AGA’s development and explored ways to support collaborative research and cross-institutional data sharing within Asia and beyond. By creating a catalogue of available Asian datasets, b, AGA seeks to enable better utilization of Asian genomic data to advance discoveries in disease susceptibility, pharmacogenomics, and precision medicine while promoting equity in global genomic research.

###

### Variant Analysis Pipeline Harmonization

This project investigated the variant calling pipelines used across different Asian countries, focusing on tools such as GATK HaplotypeCaller, DeepVariant, and others. By documenting these pipelines, the project aims to facilitate knowledge exchange, identify common practices, and explore the potential for collaboration in variant analysis. Understanding these differences will help assess the feasibility of cross-country data sharing and harmonization efforts in genomic research.

###

### CNV Analysis from Paired-End Sequence for Clinical Interpretation

This project focuses on improving the accuracy of copy number variant (CNV) detection for clinical applications by integrating two complementary approaches: breakpoint-based detection and coverage-depth analysis. By combining these methods, the project aims to enhance precision in CNV calling. Additionally, comprehensive variant annotation will improve filtering strategies, and the development of a user-friendly interface will support easier clinical interpretation, ultimately aiding in the diagnosis of genetic disorders.

###

### HPV DNA in PBMC WGS Data

Human papillomavirus (HPV) infection is a leading cause of cervical cancer, and its DNA has been detected in plasma. This project aims to develop a bioinformatics pipeline for detecting, quantifying, and characterizing HPV traces in unmapped reads from PBMC whole-genome sequencing (WGS) BAM files. By applying this approach to large-scale genomic datasets, the project seeks to provide new insights into HPV epidemiology at the population level, potentially contributing to better disease monitoring and prevention strategies.

###

### Advancing Pharmacogenomics (PGx) & Polygenic Risk Scores (PRS) for Precision Medicine

This project focuses on building a framework for implementing pharmacogenomics (PGx) and polygenic risk scores (PRS) in Asian populations to advance precision medicine. Key objectives include developing population-specific genotyping arrays using whole-genome sequencing data, automating PGx reporting based on CPIC and DPWG guidelines, and creating an automated PRS calculation system for disease risk prediction. The project also aims to assess the transferability of existing PRS models to Asian ancestries and develop tuned PRS models using LD-aware methods or de-novo GWAS. Additionally, efforts will be made to standardize clinical data frameworks for seamless PGx and PRS integration into electronic health records (EHRs), ultimately improving personalized healthcare across Asia.

###

### Federation of Trusted Research Environments (TREs)

To fully leverage the genomic and phenotypic data cataloged in the future Asian Genome-Phenome Archive, this project explored the establishment of a federation of Trusted Research Environments (TREs) that ensures both data security and research efficiency. A key challenge is aligning the TRE design with the diverse personal data protection laws and analytical needs across Asian countries. This project begins by sharing experiences and ongoing activities in each country, aiming to identify common requirements and best practices for secure and effective data access, ultimately fostering responsible data sharing and cross-border research collaboration.

###

### Ethical, Legal, and Social Issues (ELSI) in Genomic Data Sharing

This project examined the ethical, legal, and social implications (ELSI) of genomic data sharing across Asian countries. Legally, the definition of “personal information” varies by jurisdiction, raising questions about how genomic data is categorized and regulated, particularly in terms of cross-border transfers and data access. Socially, building trust within communities is essential to ensure broad acceptance of data-sharing initiatives and to prevent misunderstandings. Ethically, the project explores frameworks for equitable benefit-sharing among participating countries and research groups. By addressing these challenges, the project aims to create a responsible and sustainable foundation for genomic data governance in Asia.

###

### Federated gnomAD Aggregated Variant Browser

This project explores a federated approach to variant data sharing by partnering with the gnomAD team to develop an aggregated variant browser. Large-scale precision medicine programs across Asia, including PRECISE (Singapore), Kadoorie (China), TMM (Japan), and others, are collaborating to enable safe and secure access to population-wide variant data. Because the browser only provides aggregated variant information, it serves as a low-risk, foundational step toward cross-border genomic data sharing. This initiative aims to enhance the accessibility of genomic insights while respecting data governance frameworks in each participating country.

###

### Imputation Pipeline/Server for Thai and Asian Genome

This project aims to develop a standardized pipeline for constructing a Thai population reference panel, with a vision to expand it to include a broader range of Asian populations in the future. Initially, the focus will be on obtaining genomic data from GenomicThailand to create a Thai-specific reference panel, incorporating WGS and Human leukocyte antigen (HLA) typing data. As the project progresses, the goal is to develop a framework for extending this approach to other Asian populations, creating a more comprehensive reference panel. The protocol for making this reference panel available globally through an imputation server will also be established, with particular attention to data privacy, security, and ethical considerations. The plan for implementation includes: 1. Obtaining reference data (WGS and HLA typing), 2. Constructing the Thai-specific reference panel, 3. Validating the panel with an independent dataset, 4. Creating agreements/policies for data privacy and web server implementation, 5. Launching the imputation server.

###

### Imputation Server incorporating Japanese Haplotype References

The Database Center for Life Science, Japan has developed the TogoImputation system (https://sc.ddbj.nig.ac.jp/en/advanced_guides/TogoImputation/imputation_server/) as a Japanese version of the imputation server that is easy for Japanese researchers to use. This system is currently available in the NIG supercomputer system. Researchers can upload their own genomic data to the server and execute the imputation analysis workflow via the web user interface. After the workflow calculations are completed, the imputed genomic data, which are the results of the calculations, can be downloaded. This system can be used securely by using a virtual private network (SSL-VPN) with encrypted communication. This system uses the following programmes for imputation analysis: (i)Use conform-gt (version24May16) (https://faculty.washington.edu/browning/conform-gt.html) to convert the reference / alternative allele of the input SNP array data to match the reference panel data, (ii) Use Beagle 5.2 (version 21Apr21.304) [1] for fading and imputation analysis, and (iii) Index the genomic data (VCF file) after imputation using bcftools (version 1.9) [2]. A series of workflows are implemented in CWL and are available on GitHub (<https://github.com/ddbj/imputation-server-wf>). The TogoImputation system consists of a fully containerized and portable workflow, designed to flexibly accommodate various security requirements. It can serve as a foundational system for imputation servers in other Asian countries.

1. Browning BL, Zhou Y, Browning SR. A One-Penny Imputed Genome from Next-Generation Reference Panels. *The American Journal of Human Genetics*. 2018; doi: 10.1016/j.ajhg.2018.07.015.

2. Danecek P, Bonfield JK, Liddle J, Marshall J, Ohan V, Pollard MO, et al.. Twelve years of SAMtools and BCFtools. *GigaScience*. 2021; doi: 10.1093/gigascience/giab008.
