# Supplementary material for: Unlocking the potential of Asian genomic data: a collaborative framework for precision medicine innovation
Source: Gigascience. 2026 May 5;15:giag052. doi: 10.1093/gigascience/giag052 (PMC13196594; doi:10.1093/gigascience/giag052)
Supplement: giag052_GIGA-D-25-00441_original_submission [file giag052_giga-d-25-00441_original_submission.pdf]

GigaScience

Unlocking the Potential of Asian Genomic Data: A Collaborative Framework for Precision Medicine Innovation The MedHackathon Asia Community

--Manuscript Draft--

Manuscript Number:	GIGA-D-25-00441
Full Title:	Unlocking the Potential of Asian Genomic Data: A Collaborative Framework for Precision Medicine Innovation The MedHackathon Asia Community
Article Type:	Review
Funding Information:	
Abstract:	Asian genomic datasets possess unparalleled potential to advance global understanding of human genetic diversity. Encompassing the world's largest population pool with diverse ethnicities, these datasets capture comprehensive genomic variations shaped by heterogeneous socioeconomic conditions, climate exposures, and clinical environments. However, current national genome initiatives across Asia demonstrate substantial disunity, stemming from limited cross-border communication and collaborative infrastructure, thereby diminishing their collective impact on biomedical research and precision medicine development. The MedHackathon Asia 2025 catalyzed crucial dialogues toward establishing a regional community dedicated to three pillars: harmonized biobank collaboration, standardized genomic data protocols, and cooperative governance frameworks. This multidisciplinary convening brought together researchers, clinicians, bioinformaticians, and national precision medicine program leaders from across Asia to share best practices, identify implementation challenges, and formulate foundational strategies for sustained cooperation. This white paper synthesizes critical outcomes from these deliberations, emphasizing the imperative for continuous regional collaboration while advocating for the development of sustainable architectures enabling: (1) equitable biobank resource sharing, (2) genomic data standardization, and (3) ethical governance models. Through consolidation and expansion of this emerging network, Asian nations can lead transformative contributions to global genomic science while ensuring appropriate representation in biomedical innovation. Such coordinated efforts promise to accelerate healthcare advancements with equitable benefits extending throughout the region and worldwide.
Corresponding Author:	Tazro Ohta<br><br>JAPAN
Corresponding Author Secondary Information:	
Corresponding Author's Institution:	
Corresponding Author's Secondary Institution:	
First Author:	MedHackathon Asia Community
First Author Secondary Information:	
Order of Authors:	MedHackathon Asia Community
Order of Authors Secondary Information:	
Additional Information:	
**Question**	**Response**
Are you submitting this manuscript to a special series or article collection?	No
**Experimental design and statistics**	Yes

Powered by Editorial Manager® and ProduXion Manager® from Aries Systems Corporation

Full details of the experimental design and statistical methods used should be given in the Methods section, as detailed in our Minimum Standards Reporting Checklist. Information essential to interpreting the data presented should be made available in the figure legends.<br><br>Have you included all the information requested in your manuscript?	
**Resources**<br><br>A description of all resources used, including antibodies, cell lines, animals and software tools, with enough information to allow them to be uniquely identified, should be included in the Methods section. Authors are strongly encouraged to cite Research Resource Identifiers (RRIDs) for antibodies, model organisms and tools, where possible.<br><br>Have you included the information requested as detailed in our Minimum Standards Reporting Checklist?	Yes
**Availability of data and materials**<br><br>All datasets and code on which the conclusions of the paper rely must be either included in your submission or deposited in publicly available repositories (where available and ethically appropriate), referencing such data using a unique identifier in the references and in the “Availability of Data and Materials” section of your manuscript.<br><br>Have you have met the above requirement as detailed in our Minimum Standards Reporting Checklist?	No
If not, please give reasons for any omissions below.	This is a review article (white paper). We did not produce any new data. All materials are attached as supplementary files.

as follow-up to "**Availability of data and materials**<br><br>All datasets and code on which the conclusions of the paper rely must be either included in your submission or deposited in publicly available repositories (where available and ethically appropriate), referencing such data using a unique identifier in the references and in the "Availability of Data and Materials" section of your manuscript.<br><br>Have you have met the above requirement as detailed in our Minimum Standards Reporting Checklist?<br><br>"	
GigaScience has policies and guidelines in place for the use of generative AI-writing tools such as ChatGPT. If you have used such writing tools to assist with writing the manuscript this must be declared and cited in the text. Authors should not list AI-writing tools and other AI-assisted technologies as an author or co-author and should acknowledge that they are fully responsible for text generated or refined by AI-writing tools.<p><br><br>A summary of use (particularly in the introduction or among methods) needs to be included at the end of the paper, and the outputs should also be included as a supplementary file hosted in GigaDB or other open repositories. Please read our guidelines for more information. <p><br><br>By submitting to GigaScience, you are aware of the journal's AI-writing tools policy, and if you have declared use of such tools below, you have acknowledged this where appropriate in your manuscript and have made a summary of use and outputs available. <p><br><b>AI-assisted writing tools have been used in the preparation of this manuscript?	No

Manuscript
Click here to access/download;Manuscript;manuscript_9272025.docx

Unlocking the Potential of Asian Genomic Data: A Collaborative Framework for Precision Medicine Innovation

The MedHackathon Asia Community

(The complete list of co-authors is provided in the supplementary file)

Abstract

Asian genomic datasets possess unparalleled potential to advance global understanding of human genetic diversity. Encompassing the world's largest population pool with diverse ethnicities, these datasets capture comprehensive genomic variations shaped by heterogeneous socioeconomic conditions, climate exposures, and clinical environments. However, current national genome initiatives across Asia demonstrate substantial disunity, stemming from limited cross-border communication and collaborative infrastructure, thereby diminishing their collective impact on biomedical research and precision medicine development. The MedHackathon Asia 2025 catalyzed crucial dialogues toward establishing a regional community dedicated to three pillars: harmonized biobank collaboration, standardized genomic data protocols, and cooperative governance frameworks. This multidisciplinary convening brought

together researchers, clinicians, bioinformaticians, and national precision medicine program leaders from across Asia to share best practices, identify implementation challenges, and formulate foundational strategies for sustained cooperation. This white paper synthesizes critical outcomes from these deliberations, emphasizing the imperative for continuous regional collaboration while advocating for the development of sustainable architectures enabling: (1) equitable biobank resource sharing, (2) genomic data standardization, and (3) ethical governance models. Through consolidation and expansion of this emerging network, Asian nations can lead transformative contributions to global genomic science while ensuring appropriate representation in biomedical innovation. Such coordinated efforts promise to accelerate healthcare advancements with equitable benefits extending throughout the region and worldwide.

Introduction

Asia is home to over half of the world's population and harbors immense genetic, cultural, clinical, and climate diversity, presenting an extraordinary opportunity to enhance our understanding of human genetics and the application of next-generation precision medicine. With the growing number of national and international genome projects across the region, easy, open-minded, friendly, and efficient coordination and interaction among Asian researchers can amplify these benefits, facilitating the most impactful biomedical discoveries and clinical advancements in the world. There have been several notable attempts and achievements in the past to address the challenge of integrating diverse Asian populations and building shared genomic resources, such as the HUGO Pan-Asia SNP consortium [1] and the GenomeAsia 100K

Project [2]. And in the era of artificial intelligence (AI), which is dramatically accelerating communication among Asian countries with diverse languages and cultures, there is great potential for collaboration. This augmented collaboration across Asian countries holds the potential to unlock new breakthroughs and resource optimization, and far easier translation of genomic research into meaningful improvements in the biotech industry, public health, and individual patient healthcare across the region.

One of the key challenges facing Asian genomics research is the reliance on reference genome sequences and annotations derived primarily from Western populations. The genetic architecture of Asian populations differs significantly, influencing disease susceptibility, drug response, and other medically relevant traits [2]. Without a concerted effort to collect, share, compile coherently, and analyze genomic data that represents Asia's diverse populations accurately, precision medicine initiatives may be hampered in properly addressing the needs of people in the region.

The absence of a region-wide framework and advisory board for genomic data sharing in Asia exacerbates several challenges, including inconsistent metadata standards, ethical and regulatory barriers, and the underutilization of local biobanks. Varying privacy protection laws and data governance policies across countries create significant ethical and regulatory hurdles, slowing the sharing of genomic data and hindering cross-border collaborations [3,4]. These complexities make it difficult to harmonize privacy laws and donor consent structures, limiting access to valuable genomic datasets [5,6]. Additionally, the lack of interoperable systems further complicates data sharing, as differing national regulations restrict the ability to integrate data for larger-scale studies [7,8]. Taiwan provides a clear example of these challenges: efforts to advance

genomic data sharing have been set back by a massive data breach, which has heightened public concern and complicated policy development [9]. As a result, biobanks across the region remain underutilized, and the potential for collaborative research is severely limited, hindering the advancement of genomic research and its global impact [10,4].

To overcome these challenges, we propose a region-wide approach to genomic research that prioritizes collaboration, standardization, and continuous knowledge exchange. Establishing a strong network of local researchers across Asia is essential for bridging communication gaps and ensuring that genomic research efforts align with shared goals. By fostering an interconnected research community, we can promote the adoption of common standards for data collection, storage, and sharing, thereby enhancing the impact of national genome projects.

By working together, Asian researchers can build a more inclusive and impactful genomic research ecosystem. A well-coordinated regional approach will not only elevate the value of national efforts but also ensure that Asia contributes meaningfully to global human genetics research. The time to act is now—by fostering stronger collaborations and aligning our research frameworks, we can unlock the full potential of Asia's genomic diversity for the benefit of all.

MedHackathon Asia was initiated as a crucial step toward realizing this vision. By bringing together experts in biobanking, data analysis, and data governance, this initiative has provided a platform for researchers to discuss current challenges, share experiences, and explore strategies for harmonizing genomic research across the region. This white paper summarizes the key discussions from MedHackathon Asia 2025, emphasizing the importance of a continuous and structured effort to strengthen regional collaboration. It highlights the critical need for standardizing genomic data management, addressing regulatory challenges, and establishing a

sustainable model for cross-border data sharing.

This paper represents a summary of an ongoing discussion. Situations and policies within individual countries or initiatives may evolve over time; thus, the statements presented here do not represent the official position of any specific country or institution. Instead, they reflect the constructive and respectful dialogue among the members of our open community.

The Evolving Landscape of Biobanks in Asia

Biobanks play a pivotal role in biomedical research by providing high-quality biological specimens and comprehensive genomic data, which are essential for studying genetic diversity, understanding disease mechanisms, and advancing personalized medicine. Across Asia, biobank initiatives vary significantly in scale, governance, and accessibility, reflecting different national priorities and regulatory contexts. Japan has been proactive in biobank development since the early 2000s, with BioBank Japan, established in 2003, collecting samples from 270,000 participants and contributing significantly to genome-wide association studies (GWAS) through large-scale SNP array genotyping. Building upon advances in next-generation sequencing, the Tohoku Medical Megabank (TMM) project was launched in 2013 as a pioneering population-based cohort, sequencing whole genomes of 100,000 individuals to establish a foundational genomic reference in Asia. Collectively, these initiatives now form “Biobank Network Japan,” storing over 1,680,000 samples from both population-based cohorts and hospital-based biobanks. Additionally, Japan’s Genotype and Phenotype Archive (JGA), although not a biobank itself, serves as an essential platform enabling genomic data sharing among researchers domestically and internationally.

China has made some of the most significant recent investments in biobanking, establishing national-scale repositories and large population cohorts that position it as a central player in global genomics. The China National GeneBank (CNGB), established in 2016 in Shenzhen, functions as a national-level facility dedicated to bioresource conservation, public welfare, and innovation in life sciences. Meanwhile, the China Kadoorie Biobank, initiated in 2004, has enrolled over 510,000 participants, focusing on large-scale data collection efforts to investigate chronic diseases across diverse Chinese populations. Complementing these efforts, the Han100K Initiative systematically catalogs genetic variations across 100,000 Han Chinese individuals, providing a structured genomic resource for China [11]. More recently, Hong Kong launched its first large-scale whole-genome sequencing program through the Hong Kong Genome Project (HKGP), which began in 2021 with the goal of sequencing 45,000–50,000 genomes over five years. The project initially focuses on undiagnosed diseases and hereditary cancers to support the integration of genomics into clinical care [12]. Taiwan has also made significant strides, with the Taiwan Biobank (TWB), established in 2012, enrolling more than 150,000 participants (toward a target of 200,000) and linking genomic data to long-term national health insurance and registry records, enabling robust longitudinal studies [9]. In addition, the Taiwan Precision Medicine Initiative (TPMI), launched in 2019, is building a 1-million-person, hospital-based cohort through a network of 16 major medical centers, with over half of the target already enrolled and genotyped [13]. Although China, Hong Kong and Taiwan are not currently participating in the MedHackathon Asia 2025, their growing contributions to genomic research are highly valued, and we warmly welcome their engagement in future collaborative efforts and upcoming regional gatherings.

Singapore’s National Precision Medicine Programme completed its initial phase (2017–2021) with 10,000 participants and is currently in Phase II (2021–2025), aiming to gather genomic data from an expanded cohort of 100,000 individuals to support precision medicine research. South Korea’s Korean Genome Project and India’s GenomeIndia initiative are also progressively enhancing their genomic databases, although their access models vary significantly due to different regulatory approaches. Thailand’s Genomics Thailand initiative, launched in 2019, specifically targets disease- and ethnicity-related research and emphasizes controlled access to protect data privacy and security [14]. In addition, Indonesia’s BGSi initiative, established in 2022, focuses primarily on disease-specific studies and represents an emerging contribution to the biobanking landscape in the region **(Table 1)**.

Table 1. Summary of Asian biobanks. (See Supplementary Table S1 for full details.)

Jurisdiction	Resource name	Participants	Sample type (healthy/disease)	Accessibility	Since
Thailand	Genomics Thailand	50K	disease/ethnics	Aggregated data publicly available.	2019
Japan	Tohoku Medical Megabank (TMM)	150K	population	Controlled	2011
Japan	BioBank Japan	270K	disease	global - requested	2003
Japan	Biobank Network Japan	800K	disease	Controlled	2018
Japan	JGA (Japanese Genotype-phenotype	800K	population/healthy/ disease	Open/Controlled	2013

	Archive)				
Singapore	SG10K	10K	healthy	Managed-access through a DAC	2017
Singapore	PRECISE-SG100K cohort	100K	healthy	Managed-access through a DAC	2021
South Korea	Korean Genome Project	10K	population/healthy/ disease	Partly Open	2015
India	GenomeIndia	≈19K	population	Supposedly open, but policies still being drafted	2020
Indonesia	Biomedical and Genome Science Initiative (BGSi)	≈4K	disease	Regulated by Indonesia's Ministry of Health.	2022
Hong Kong*	Hong Kong genome project (HKGP)	≈50k	disease	local access; global researchers by approval (de-identified data for approved studies)	2021
Taiwan*	Taiwan Biobank (TWB)	≈200K (ongoing)	population	global via application (IRB approval & fee required)	2012
Taiwan	Taiwan Precision Medicine Initiative (TPMI)	≈500K (enrolled; aim 1M)	various conditions	Open/Controlled approved researchers (partner hospitals & Academia Sinica)	2019
Mainland China*	China Kadoorie Biobank (CKB)	510K	Chronic non-communicable diseases (NCDs)	Controlled global access on request (bona fide researchers apply; data delivered on approval)	2004
Mainland	Han100K	100K	Natural populations	Web-based, Open	2019

China					
Mainland China	Chinese Pangenome Consortium (CPC)	≈1K	Natural populations	Web-based, Open	2021
Asia-Pacific	Asian Pangenome Consortium (APC)	100K (to be enrolled)	Natural populations	Web-based, Open	2025

Note: "*" indicates there was no official representative from that jurisdiction at MEDhackathon Asia 2025.

<u>Data Governance and Data Sharing Policies Across Biobanks</u>

Managing genomic data governance and sample accessibility is a global challenge due to varying privacy laws, data-sharing regulations, and national policies. Asian countries similarly encounter these complexities, generally following comparable approaches for domestic data sharing—typically through Data Access Committees (DACs) or equivalent approval processes, as observed in Japan, South Korea, Thailand, and Singapore. However, cross-border genomic data sharing is substantially more challenging and involves additional considerations, notably personal information protection and national security concerns. Personal information protection governs privacy and data handling, facilitating easier data exchange among countries with comparable standards, such as GDPR equivalence. In contrast, national security concerns—including the classification of genomic data as a potential strategic national resource—complicate international data sharing, leading to stricter regulations or prohibitions. Recent reviews indicate that, despite ASEAN member states having developed individual regulatory arrangements, overlapping legacy frameworks and data sovereignty often impede seamless regional data sharing, underscoring the critical need for consolidated and harmonized governance frameworks

[15].

Country-specific Data Sharing Policies

Asian countries generally adopt a combination of open and controlled-access policies depending on data sensitivity. While aggregated statistical data from biobanks are often openly accessible, individual-level data and biological samples typically require ethical approval from committees such as Institutional Review Boards (IRBs) or DACs. For instance, Japan, South Korea, Thailand, and Singapore follow this general model with slight differences: accessing individual-level data internationally generally requires formal research collaboration agreements with local researchers or institutions, as exemplified by Singapore's managed-access approach. Similarly, Thailand's individual-level data access is currently restricted to domestic researchers, with potential for future expansion. China's genetic data policies reflect a dual commitment to fostering biomedical innovation and safeguarding national interests. While recent reforms have relaxed some controls, strict oversight—particularly for international collaborations—remains. Researchers must navigate a complex landscape of approvals, ethical reviews, and data localization requirements. Despite these commonalities, understanding precise differences in each country's policies remains challenging due to varying legal frameworks and cultural contexts, further emphasizing the importance of working toward harmonized governance frameworks.

Role of Digital Platforms and Infrastructure

Digital platforms play a crucial role in operationalizing data governance and ensuring secure data

access and management. The Genomics Thailand initiative employs multiple digital tools, including an SMS platform for real-time updates, a Variant Annotation and Prioritization Platform (V@PP) for genomic analysis, and the Thai Genome Reference Database (ThaiGeR), along with a Secure Data Environment (SDE) featuring an "Air-lock" mechanism to restrict direct data downloads. Additionally, the Thai Exploratory Aggregated Genome Database (ThxAD) further enhances data exploration. Japan's advanced systems, such as the Tohoku University Tohoku Medical Megabank Organization (ToMMo) Supercomputer and Data Browser, provide multi-tiered access tailored to diverse research needs. Singapore's TRUST platform offers a managed-access, cloud-based environment balancing data accessibility and security. Meanwhile, South Korea's Korean Genome Project follows an open-access and open-source model, while GenomeIndia and Indonesia's BGSi utilize managed, or hybrid approaches tailored to their regulatory and cultural environments.

Emerging Models of Consent and Ethical Considerations

Beyond data access policies and technological solutions, informed consent mechanisms are crucial for ethically sound and sustainable biobanking practices. Emerging models, such as dynamic consent—which allows participants to modify consent preferences in real time—have been explored as potential solutions to address limitations inherent in traditional one-time consent processes [16]. However, dynamic consent faces practical challenges, particularly regarding legacy samples, deceased donors, and long-term participant engagement. As alternatives, approaches emphasizing careful regulation of data usage ("exit control") or frameworks treating health data as common goods ("data commons") are gaining attention. The

European Health Data Space (EHDS) and ongoing discussions on the ownership and governance of individual-level health data further underscore the importance of ensuring individual autonomy, transparency, and societal sustainability in data-sharing practices [17].

Towards Inclusive Dialogue and Regional Harmonization

Addressing these governance challenges will benefit significantly from continued and open dialogue among biobank researchers and genomic scientists across Asia. Forming collaborative communities or advisory boards to facilitate these discussions is a valuable possibility that warrants exploration. However, such efforts must respect each country's unique policies, guidelines, and cultural contexts. MedHackathon discussions emphasized that all Asian countries should have the opportunity to engage and contribute, ensuring inclusive participation without pressure towards any particular governance model. Ultimately, mutual understanding, ongoing cooperation, and collaborative development of harmonized policies will be essential for leveraging Asia's genomic diversity effectively and responsibly in biomedical research.

Balancing Data Security and Research Accessibility

A persistent challenge for biobank management is achieving a balance between robust data security and facilitating meaningful research access. Controlled-access repositories, such as Japan's NBDC Human Database and South Korea's controlled-access genome archive, employ tiered clearance systems based on data sensitivity. Data Access Committees (DACs) are essential in this context, rigorously reviewing and regulating access to ensure that both ethical and scientific standards are met.

Towards a Harmonized Data Governance Framework

Given the inherent challenges in cross-border data sharing, the development of an integrated governance framework is imperative. Federated data-sharing models, like those advocated by the Global Alliance for Genomics and Health (GA4GH), enable decentralized data analyses without necessitating the transfer of raw data, thereby mitigating many associated risks. In tandem, standardizing metadata formats and data submission guidelines across biobanks can facilitate interoperability and streamline multi-country research initiatives. Ultimately, policy alignment among Asian nations—through the establishment of common ethical guidelines, enhanced institutional cooperation, and proactive policymaker engagement—is crucial for developing a unified framework that not only secures data but also maximizes its scientific utility. Such harmonization is essential for advancing genomic research and improving healthcare outcomes across the region.

Fostering Collaborative Genomic Innovation

Sharing knowledge and technologies in Asian genomics is emerging as a vital strategy to overcome regional fragmentation and drive collaborative progress in precision medicine. MedHackathon Asia 2025 serves as an excellent initiation of this collaborative approach by uniting diverse experts to standardize methodologies, streamline computational processes, and build shared data resources that capture the region's rich genetic diversity.

The region faces an urgent need for common data standards. The **Asian Pangenome Initiative** undertakes a detailed review of human pangenome studies across Asia by evaluating

sequencing technologies, assessing genome assembly quality, and identifying methodological gaps. This effort informs the development of uniform data standards that support robust comparative research across diverse Asian populations.

At the same time, significant projects are working to harmonize computational pipelines for genomic analysis. The **Variant Analysis Pipeline Harmonization** project catalogs and compares tools used for variant calling to develop uniform protocols for genetic interpretation. In addition, the **CNV Analysis for Clinical Interpretation** project is developing a comprehensive pipeline that integrates methods for detecting copy number variants using both breakpoint detection and coverage-depth approaches. A dedicated team is also developing a pipeline for the **HPV DNA Detection in PBMC whole-genome sequencing (WGS) Data** project to detect and quantify traces of human papillomavirus DNA from whole-genome sequencing data of peripheral blood mononuclear cells. The **Imputation Pipeline/Server for Thai and Asian genome** project aligns with the imputation workflow that incorporates Japanese reference panels [18] and is implemented using common workflow language (CWL) [19]. This initiative further contributes by establishing a standardized genotype imputation framework that is tailored to the Asian and Thai population **(Figure *1*)**.

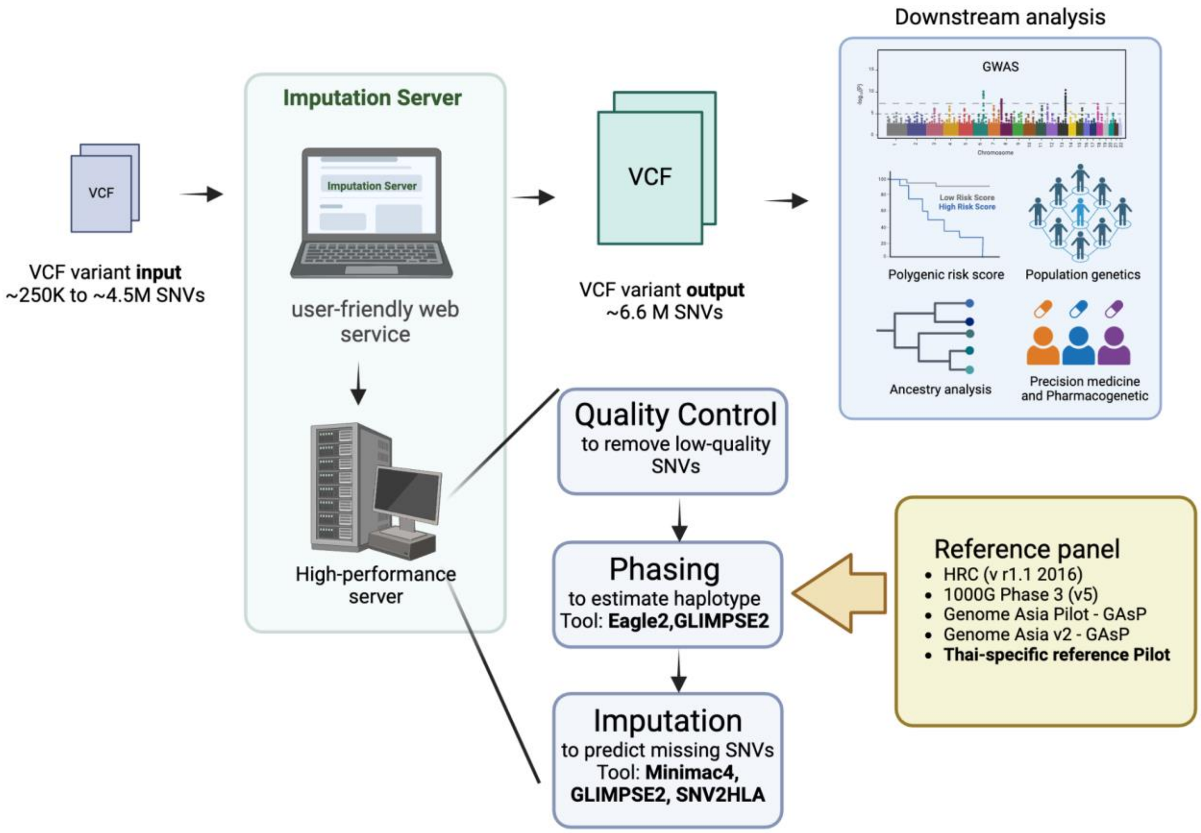

Figure 1. The concept of the imputation server with the Asian and Thai reference panel. The figure illustrates a standardized genotype imputation workflow tailored to Asian populations, incorporating high-quality reference panels such as HRC (v1.1), 1000 Genomes Project Phase 3, Genome Asia Pilot (GAsP), and a Thai-specific reference pilot. The imputation process begins with user-submitted VCF files (approximately 250K to 4.5M SNVs), processed through a user-friendly web service hosted on a high-performance computing server. Uploaded variants undergo quality control to remove low-quality SNVs, followed by haplotype phasing using tools such as Eagle2 and GLIMPSE2, and imputation to predict missing SNVs using Minimac4, GLIMPSE2, or SNV2HLA. The resulting VCF file (~6.6M SNVs) supports downstream analyses, including genome-wide association studies (GWAS), polygenic risk scoring, ancestry analyses, precision medicine, and population genetics research. This standardized approach promotes harmonized genomic analyses across Asian populations, facilitating regional collaboration and improved accuracy in genetic interpretations.

Interoperability and robust metadata management are critical to promoting cross-border collaboration. The **Asian Genome Phenome Archive** is a project that seeks to build a

comprehensive regional catalogue of genomic and phenotypic datasets, complete with detailed metadata and clearly defined contact points **(Figure *2*)**. Complementing this effort is the **Federated gnomAD Aggregated Variant Browser**, which aims to integrate allele frequency data from multiple Asian projects to improve data accessibility. Another initiative focuses on establishing a **Federation of Trusted Research Environments** to provide secure and decentralized platforms for data sharing while ensuring compliance with legal requirements across countries.

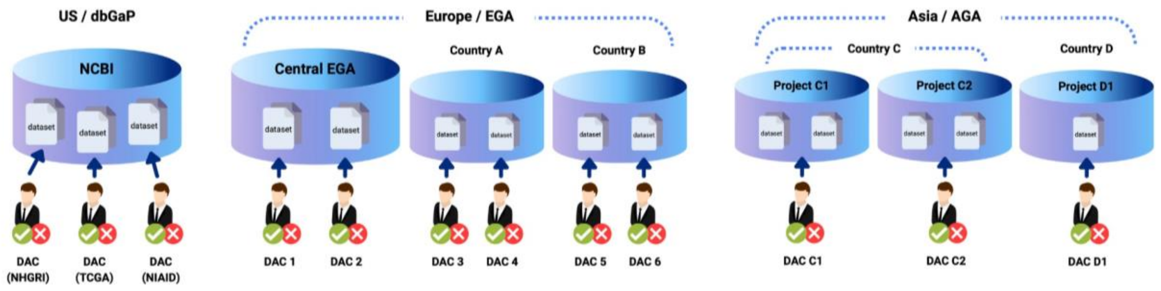

Figure 2. Comparison of data repository structures: dbGaP (USA), EGA (Europe), and the proposed Asian Genome-Phenome Archive (AGA). This figure illustrates conceptual differences in governance and data access among genomic data repositories in the United States (dbGaP), Europe (EGA), and the proposed Asian Genome-Phenome Archive (AGA). In the USA, a centralized repository is managed by the National Center for Biotechnology Information (NCBI), and Data Access Committees (DACs) from individual NIH institutes (e.g., NHGRI, TCGA, NIAID) control access to the data they fund. Europe's EGA is similarly centralized under the European Bioinformatics Institute (EBI), but individual countries may also host their own federated EGA instances, with data submitters designating which DAC controls their datasets, often forming their own committees. In contrast, the proposed AGA model in Asia suggests that each genomic research project or biobank independently manages its repository and DAC, providing permissions to all datasets within their repositories. Additionally, while dbGaP and EGA typically manage biological specimens independently from digital data (metadata, nucleotide sequences), the AGA allows biobanks themselves to archive both digital data and biological specimens in an integrated manner

Capacity building and training are essential to ensure that technological advancements are matched by a skilled workforce. The **Advancing Pharmacogenomics and Polygenic Risk Scores for Precision Medicine** project is developing a genotyping array tailored to Asian populations and automating the reporting of pharmacogenomic profiles and polygenic risk scores. This initiative not only standardizes clinical and analytical frameworks but also offers hands-on training opportunities for clinicians, bioinformaticians, and researchers. Such capacity building is crucial for integrating genomic medicine into routine clinical practice and for supporting long-term advancements in precision medicine.

MedHackathon Asia 2025 provides a dynamic platform for sharing knowledge and technologies across Asia. By facilitating collaborative projects that address common challenges and promote innovative solutions, the event lays the groundwork for a more integrated and impactful genomic research ecosystem throughout the region. **(Supplementary File S2 - Project descriptions)**

Future perspectives

The vision for genomic research in Asia depends on the successful implementation of standardized data sharing protocols and the development of an integrated data ecosystem. In a future where centralized guidelines and federated data sharing models are widely adopted, Asian institutions would follow uniform procedures for data collection, submission, and metadata annotation. Such standardization would facilitate secure, decentralized analyses while upholding local ethical and regulatory requirements **(Figure *3*)**. MedHackathon Asia 2025 has already initiated this collaborative process by inspiring prototype concepts, such as a unified pangenome framework and a Thai-specific imputation server, which, although still in the conceptual stage, offer promising case studies for validating these protocols.

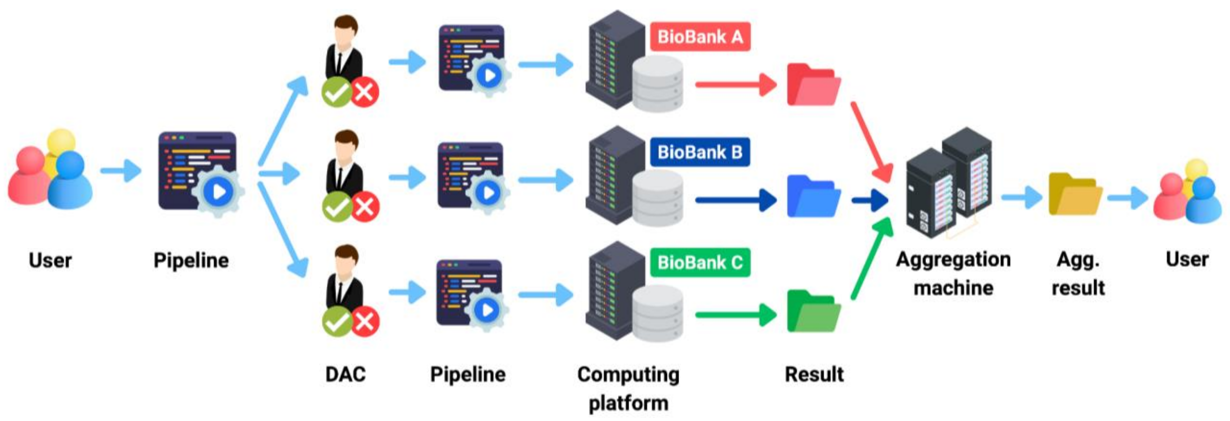

Figure 3. Proposed framework on data sharing across Asian countries. The figure outlines a conceptual framework for secure and decentralized genomic data sharing among biobanks and research institutions across Asia. Under this model, data analysis workflows or pipelines initiated by users undergo approval by each institution's Data Access Committee (DAC). Upon approval, the workflows are executed on secure computing platforms managed individually by each biobank, ensuring data privacy and regulatory compliance. Results from multiple biobanks are then aggregated securely, enabling the users to access combined analytical outcomes without direct access to raw data. This federated approach facilitates collaborative research across Asian countries while fully respecting each country's ethical guidelines, regulatory requirements, and data sovereignty.

However, to sustain the technical collaboration among institutes, it is necessary to revive or launch a new control tower that is bottom-up and objective-driven by participating researchers. Conferences and even Asian researcher-initiated journals will be necessary to educate the next generation of genomics researchers with a steady ecosystem of paper publication and data sharing in the region. The key problem of failing to have such an Asian control committee or organization is that the ever-changing political environment of all the nations keeps disrupting science-driven guidelines, funding, and policies. Once such a non-government initiated bottom-up virtual organization is formed with minimal sustainable funding by region's governments, autonomous and more objective sustainable biobanks, genome projects, and applications towards other areas of biomedicine. This will enable Asian researchers to efficiently cope with

international organizations such as GA4GH and even lead the biobank resources and future precision projects of the world. As AI and distributed information exchange systems such as blockchain are advancing fast, Asia's enormous body of diversified data and human resources can play a pivotal role in human health and longevity.

Looking forward, if maximum data sharing across Asian genomic initiatives with proper research-driven control towers is achieved, the impact on both research and clinical practice for the world could be profound. An integrated data ecosystem that incorporates diverse genomic and phenotypic datasets would empower researchers to perform large-scale meta-analyses, uncover population-specific genetic insights, and develop innovative computational approaches to disease prediction and personalized treatment. This comprehensive data sharing would, in turn, support transformative advances in precision medicine and public health.

The potential applications of such an open data environment are multifaceted. In the field of population genetics and evolutionary studies, the vast genetic diversity of Asia, shaped by centuries of migration, adaptation, and environmental influences, could be analyzed in unprecedented detail. Researchers would be able to elucidate the genetic structure of diverse ethnic groups, trace historical migration patterns, and identify adaptations unique to specific populations. This inclusive approach would correct the current bias toward Western centric datasets and ensure that underrepresented groups contribute to and benefit from global genomic research.

Moreover, enhanced data sharing would facilitate precision medicine tailored to Asian populations. By leveraging shared data, researchers could identify genetic risk factors and develop polygenic risk scores that more accurately reflect the genetic architecture of diseases

prevalent in Asia, such as hepatocellular carcinoma, nasopharyngeal cancer, and thalassemia. The pooling of data from multiple countries would also enable more effective studies of rare diseases, where limited sample sizes in individual nations have historically constrained research efforts.

In the realm of public health and epidemiology, a fully integrated open and transparent data framework will enable real-time genomic surveillance to track infectious diseases and monitor viral evolution. By linking genomic data with clinical and epidemiological information, it would be possible to identify genetic markers associated with disease severity or vaccine response, thereby informing targeted public health interventions.

MedHackathon Asia 2025 has thus established a promising initial foundation for collaborative data sharing in the region. With the adoption of standardized protocols and the realization of a comprehensive, secure data ecosystem, the collective sharing of genomic resources can substantially accelerate scientific discovery and revolutionize precision medicine across Asia.

References

1. Ngamphiw C, Assawamakin A, Xu S, Shaw PJ, Yang JO, Ghang H, et al.. PanSNPdb: The Pan-Asian SNP Genotyping Database. *PLOS ONE*. Public Library of Science; 2011; doi: 10.1371/journal.pone.0021451.

2. Wall JD, Stawiski EW, Ratan A, Kim HL, Kim C, Gupta R, et al.. The GenomeAsia 100K Project enables genetic discoveries across Asia. *Nature*. 2019; doi: 10.1038/s41586-019-1793-z.

3. Byrd JB, Greene AC, Prasad DV, Jiang X, Greene CS. Responsible, practical genomic data sharing that accelerates research. *Nature Reviews Genetics*. 2020; doi: 10.1038/s41576-020-0257-5.

4. Brunfeldt M, Teare H, Soini S, Kääriäinen H. Perceptions of legislation relating to the sharing of genomic biobank results with donors—a survey of BBMRI-ERIC biobanks. *European Journal of Human Genetics*. 2018; doi: 10.1038/s41431-017-0049-3.

5. Takai-Igarashi T, Kinoshita K, Nagasaki M, Ogishima S, Nakamura N, Nagase S, et al.. Security controls in an integrated Biobank to protect privacy in data sharing: rationale and study design. *BMC Medical Informatics and Decision Making*. 2017; doi: 10.1186/s12911-017-0494-5.

6. Sanderson SC, Brothers KB, Mercaldo ND, Clayton EW, Antommaria AHM, Aufox SA, et al.. Public Attitudes toward Consent and Data Sharing in Biobank Research: A Large Multi-site Experimental Survey in the US. *The American Journal of Human Genetics*. Elsevier; 2017; doi: 10.1016/j.ajhg.2017.01.021.

7. Mandl KD, Glauser T, Krantz ID, Avillach P, Bartels A, Beggs AH, et al.. The Genomics Research and Innovation Network: creating an interoperable, federated, genomics learning system. *Genetics in Medicine*. Elsevier; 2020; doi: 10.1038/s41436-019-0646-3.

8. Knoppers BM, Harris JR, Tassé AM, Budin-Ljøsne I, Kaye J, Deschênes M, et al.. Towards a data sharing Code of Conduct for international genomic research. *Genome Medicine*. 2011; doi: 10.1186/gm262.

9. Lin J-C, Fan C-T, Liao C-C, Chen Y-S. Taiwan Biobank: making cross-database convergence possible in the Big Data era. *GigaScience*. 2018; doi: 10.1093/gigascience/gix110.

10. Garrison N, Sathe NA, Antommaria AHM, Holm IA, Sanderson SC, Smith ME, et al.. A systematic literature review of individuals' perspectives on broad consent and data sharing in the

United States. *Genetics in Medicine*. Elsevier; 2016; doi: 10.1038/gim.2015.138.

11. Gao Y, Zhang C, Yuan L, Ling Y, Wang X, Liu C, et al.. PGG.Han: the Han Chinese genome database and analysis platform. *Nucleic Acids Research*. 2020; doi: 10.1093/nar/gkz829.

12. Annie T. W. Chu, Jasmine L. F. Fung, Amy H. Y. Tong, Sin Man Chow, Kelvin Y. K. Chan, Kit San Yeung, et al.. Potentials and challenges of launching the pilot phase of Hong Kong Genome Project. *Journal of Translational Genetics and Genomics*. 2022; doi: 10.20517/jtgg.2022.02.

13. Yang H-C, Kwok P-Y, Li L-H, Liu Y-M, Jong Y-J, Lee K-Y, et al.. The Taiwan Precision Medicine Initiative: A Cohort for Large-Scale Studies. *bioRxiv*. 2024; doi: 10.1101/2024.10.14.616932.

14. Shotelersuk V, Tongsima S, Pithukpakorn M, Eu-ahsunthornwattana J, Mahasirimongkol S. Precision medicine in Thailand. *American Journal of Medical Genetics Part C: Seminars in Medical Genetics*. John Wiley & Sons, Ltd; 2019; doi: 10.1002/ajmg.c.31694.

15. Medina PB, Armon S, Bin Abdul Aziz MF, Cheong IH, de Leon MP, Drobysz S, et al.. A Review of Regulatory Frameworks for Biobanking in Southeast Asia. *Biopreservation and Biobanking*. Mary Ann Liebert, Inc., publishers; 2025; doi: 10.1089/bio.2024.0044.

16. Budin-Ljøsne I, Teare HJA, Kaye J, Beck S, Bentzen HB, Caenazzo L, et al.. Dynamic Consent: a potential solution to some of the challenges of modern biomedical research. *BMC Medical Ethics*. 2017; doi: 10.1186/s12910-016-0162-9.

17. Piasecki J, Cheah PY. Ownership of individual-level health data, data sharing, and data governance. *BMC Medical Ethics*. 2022; doi: 10.1186/s12910-022-00848-y.

18. Hachiya T, Ishii M, Kawai Y, Khor S-S, Kawashima M, Toyo-Oka L, et al.. The NBDC-DDBJ imputation server facilitates the use of controlled access reference panel datasets in Japan. *Human Genome Variation*. 2022; doi: 10.1038/s41439-022-00225-6.

19. Crusoe MR, Abeln S, Iosup A, Amstutz P, Chilton J, Tijanić N, et al.. Methods included: standardizing computational reuse and portability with the Common Workflow Language. *Commun ACM*. New York, NY, USA: Association for Computing Machinery; 2022; doi: 10.1145/3486897.

Click here to access/download
Supplementary Material
Author_list.xlsx

Click here to access/download
Supplementary Material
SupplementaryTable_S1_9272025.xlsx
