# Supplementary material for: Unlocking the potential of Asian genomic data: a collaborative framework for precision medicine innovation
Source: Gigascience. 2026 May 5;15:giag052. doi: 10.1093/gigascience/giag052 (PMC13196594; doi:10.1093/gigascience/giag052)
Supplement: giag052_GIGA-D-25-00441_revision_1 [file giag052_giga-d-25-00441_revision_1.pdf]

GigaScience

Unlocking the Potential of Asian Genomic Data: A Collaborative Framework for Precision Medicine Innovation

--Manuscript Draft--

**Manuscript Number:**	GIGA-D-25-00441R1
**Full Title:**	Unlocking the Potential of Asian Genomic Data: A Collaborative Framework for Precision Medicine Innovation
**Article Type:**	Review
**Funding Information:**	
**Abstract:**	Asian genomic datasets possess unparalleled potential to advance global understanding of human genetic diversity. Encompassing the world's largest population pool with diverse ethnicities, these datasets capture comprehensive genomic variations shaped by heterogeneous socioeconomic conditions, climate exposures, and clinical environments. However, current national genome initiatives across Asia demonstrate substantial disunity, stemming from limited cross-border communication and collaborative infrastructure, thereby diminishing their collective impact on biomedical research and precision medicine development. The MedHackathon Asia 2025 catalyzed crucial dialogues toward establishing a regional community dedicated to three pillars: harmonized biobank collaboration, standardized genomic data protocols, and cooperative governance frameworks. This multidisciplinary convening brought together researchers, clinicians, bioinformaticians, and national precision medicine program leaders from across Asia to share best practices, identify implementation challenges, and formulate foundational strategies for sustained cooperation. This community review synthesizes critical outcomes from these deliberations, emphasizing the imperative for continuous regional collaboration while advocating for the development of sustainable architectures enabling: (1) equitable biobank resource sharing, (2) genomic data standardization, and (3) ethical governance models. Through consolidation and expansion of this emerging network, Asian nations can lead transformative contributions to global genomic science while ensuring appropriate representation in biomedical innovation. Such coordinated efforts promise to accelerate healthcare advancements with equitable benefits extending throughout the region and worldwide.
**Corresponding Author:**	Tazro Ohta<br><br>JAPAN
**Corresponding Author Secondary Information:**	
**Corresponding Author's Institution:**	
**Corresponding Author's Secondary Institution:**	
**First Author:**	MedHackathon Asia Community
**First Author Secondary Information:**	
**Order of Authors:**	MedHackathon Asia Community
**Order of Authors Secondary Information:**	
**Response to Reviewers:**	Response to Reviewer #1<br><br>We sincerely thank the reviewer for the thoughtful and supportive assessment of our manuscript. We are grateful that you found the topic timely and relevant, and we appreciate your recognition of the collective expertise represented in the MedHackathon Asia community.<br><br>We acknowledge the reviewer’s concern regarding the difficulty in distinguishing which statements are grounded in existing academic literature and policy, and which

Powered by Editorial Manager® and ProduXion Manager® from Aries Systems Corporation

represent recommendations or perspectives developed through discussions at MedHackathon Asia 2025. In response, we have added missing citations to attribute statements that draw on existing research or online resources. We have also refined the language in sections containing recommendations or viewpoints from the hackathon discussions to ensure these contributions are clearly distinguished from literature-based descriptions.

We believe these revisions significantly improve clarity for the reader and better highlight the contributions of both the academic literature and the hackathon community. We thank the reviewer again for helping us strengthen the manuscript.

> ## Abstract
> I think the term "Whitepaper" is used in many different ways, often it applies to not-peer-reviewed papers too. I would suggest replacing this with a more appropriate term.

We thank the reviewer for this suggestion. We agree that the term “whitepaper” may be ambiguous and have replaced it with “community review” to more accurately reflect the nature of this manuscript.

> ## Intro
> "in the era of artificial intelligence (AI), which is dramatically accelerating communication among Asian countries with diverse languages and cultures, there is great potential for collaboration." -> I'm not 100% sure I follow the argument, is this about machine translation that enables communication between those countries? It would be nice to clarify this.

Our intention was not only to refer to AI-enabled tools such as machine translation that can facilitate communication across linguistic and cultural boundaries, but also to highlight that the era of AI increases the value of high-quality, diverse datasets. This creates new opportunities for Asian countries to collaborate and collectively maximize the impact of their unique genomic and health resources. We have revised the first paragraph of the Introduction section to clarify this point and make the connection between AI and collaborative potential more explicit.

> The example of the data breach in Taiwan seems a bit out of place: In the sentences before, the authors highlight the challenges of privacy laws & consent structures, as well as lack of interoperability. But I'm not sure how a data breach demonstrates something across those factors? It would be good to embed this better/expand the argument appropriately.

We agree that the previous phrasing did not clearly connect the “breach” example to the surrounding discussion of privacy/consent requirements and interoperability. In revising this section, we also realized that our earlier mention of a “Taiwan Biobank data breach” was not supported by an official confirmation and could therefore be misleading. We have removed this unconfirmed example to avoid an unintended or inaccurate argument, and revised the section to more clearly emphasize the broader challenges biobanks face in cross-border collaboration. We sincerely thank the reviewer for raising this point and helping us correct the manuscript.

> It would be good to get some demographics/statistics of the hackathon: how many people and from which regions/countries did participate in the event and later write-up? Later on it's mentioned that there weren't participants from HK/China/TW, but readers don't get an idea of who was in the room

To provide clearer context on the perspectives represented, we have added a short paragraph and new Figure 1 summarizing the number of participants and the countries and regions involved in both the MedHackathon Asia 2025 event and the subsequent manuscript development. We agree that this information helps readers understand who contributed to the discussions and clarifies the geographic scope of the community.

> ## Biobanks
> The current structure on biobanks (section "The Evolving Landscape of Biobanks in Asia") flows a bit strangely, I presume all mentioned countries except for HK/China/Taiwan participated in the Hackathon? If that's the case it feels a bit strange

	to break the flow to go from the participating countries into a large block of non-participating countries, before heading back to particpating ones.<br><br>We agree that the current structure may feel uneven, as it shifts between countries that participated in the hackathon and those that did not. To improve readability and clarity, we have revised the section to first summarize the biobanking landscape based on the experiences shared by participating countries, followed by a separate paragraph describing major initiatives in regions without representation at the event (e.g., China, Hong Kong, Taiwan). This restructuring provides a more coherent narrative while ensuring comprehensive regional coverage.<br><br>> While the sections on Japan & China are well-sourced, it would be good if the authors could also include references to the other biobanks and efforts, in particular for Singapore, South Korea, India and Indonesia<br>We have added appropriate references for these resources in the revised manuscript.<br><br>> The section on "Data Governance and Data Sharing Policies Across Biobanks" lacks citations, with only one given so far. I appreciate that there would be lots of different regulations that could apply across nations, but it would be good to at least give a some concrete examples (e.g. which countries apply national security concerns to genetic data?)<br><br>We agree that the section would benefit from more concrete, well-sourced examples. In the revision, we added additional references to national data governance frameworks and published discussions of genomic data governance and national-security considerations where authoritative sources are available. We also added a comparative table (Japan, Singapore, and Thailand) that summarizes key governance and access features (e.g., domestic vs. international access, hosting/localization models, and approval/ethics review processes) to make the policy differences more explicit and actionable for readers.<br><br>> The same lack of sources applies to the country-specific data sharing policies section and the role of digital platforms/infrastructure sections. Many systems are called out in the latter section in particular, but none are given citations.<br><br>We thank the reviewer for highlighting the need for additional sourcing in these sections. In the revised manuscript, we have added references for the data-sharing policies and digital platforms mentioned, including official documentation, published descriptions, or institutional websites when peer-reviewed sources are not available.<br><br>> The "Emerging models of consent" section mentions "approaches emphasizing careful regulation of data usage" that are gaining attention. By whom do they gain attention and is this the case in Asia or generally? A reference would be appreciated here too<br><br>The intention of this section was to reflect a trend observed both in Asia and internationally, based on discussions among MedHackathon participants as well as broader developments in biobank ethics. To avoid ambiguity, we have revised the text to clarify that these approaches, such as strengthened data-use regulation (“exit control”) and data commons models, are gaining attention in global discussions on biobank governance. We have also added appropriate citations to support this statement.<br><br>> "Balancing Data Security and Research Accessibility": Are those the opinions of the hackathon attendees or generally accepted, citeable facts?<br><br>The statements in this section reflect both the perspectives shared by MedHackathon attendees and widely recognized challenges discussed in the biobanking and data governance literature. To avoid ambiguity, we have revised the text to clarify this and have added citations where suitable sources are available.<br><br>> Towards a harmonized data governance framework: please reference GA4GH's work you mention. Otherwise: Same as above, are the rest of the statements claims made by the attendees/authors or based on existing work?

Powered by Editorial Manager® and ProduXion Manager® from Aries Systems Corporation

We have added citations to the relevant Global Alliance for Genomics and Health (GA4GH) frameworks mentioned in this section.

> ## Fostering Collaborative Genomic Innovation
> Are the different, bolded "teams" or "projects" in this section teams that formed during the hackathon? If so that's not fully clear and should be expanded upon, otherwise please reference the external projects

The bolded teams and projects in this section represent collaborative groups that formed during MedHackathon Asia 2025, rather than pre-existing external initiatives. To make this clearer for readers, we added Table 3 (“Summary of Collaborative Initiatives and Projects Launched at MedHackathon Asia 2025”), which lists these teams/projects and summarizes their scope and outputs.

Response to Reviewer #2

> Overall Assessment
> This whitepaper provides a timely and thoughtful overview of the rapidly evolving genomics landscape in Asia. It brings together a wide range of national initiatives, describes the governance challenges and opportunities for regional collaboration in Asian genomics, and highlights the technical efforts that emerged from MedHackathon Asia 2025. This topic is timely and important: the underrepresentation of Asian genomics is a major goal gap, and consolidated regional infrastructure and collaboration can significantly improve precision medicine for a large portion of the world's population.
> Overall, this whitepaper lays a strong foundation for future collaborative efforts in Asian genomics and will be of strong interest to readers working on large-scale biobanks, international data governance, and precision medicine implementation.

We sincerely thank the reviewer for the thoughtful and encouraging assessment of our manuscript. We greatly appreciate your recognition of the importance of strengthening regional collaboration in Asian genomics and your acknowledgement that this work lays a meaningful foundation for future initiatives. Your positive feedback on the relevance and timeliness of this paper is highly motivating for our community. We thank you again for your constructive comments and for supporting the advancement of collaborative genomic efforts across Asia.

> #1: In Supplementary Table 1, the authors provide a comprehensive summary of biobanks across Asia. To enhance its comprehensiveness, the authors may wish to incorporate a few additional regional initiatives, including:
> ChinaHEART: https://academic.oup.com/ije/article/52/5/e273/7187439
> GenomeAsia 100k: https://www.nature.com/articles/s41586-019-1793-z
> Pakistan Genome Resource: https://www.nature.com/articles/nature22034

We thank the reviewer for these valuable suggestions. We have incorporated GenomeAsia 100K and the Pakistan Genome Resource into Table1 and Supplementary Table 1 as recommended.
Regarding ChinaHEART, after reviewing the cited publication, we found that the initiative primarily focuses on large-scale cardiovascular epidemiology and clinical data collection, without establishing a biobank for biological specimens or generating genomic datasets. As our table specifically summarizes biobanks and genomic resources, we believe ChinaHEART does not fit the scope of this section. Therefore, we have not included it in the revised table.

> #2: The manuscript identifies governance challenges well, but could benefit from a slightly deeper discussion on policy and governance examples. For instance, in page 10, the authors note:
> "Researchers must navigate a complex landscape of approvals, ethical reviews, and data localization requirements. Despite these commonalities, understanding precise differences in each country's policies remains challenging due to varying legal frameworks and cultural contexts, further emphasizing the importance of working toward harmonized governance frameworks."
> While Supplementary Table 1 summarizes platforms and access policies across biobanks, the manuscript would be strengthened by providing more detail on:

Powered by Editorial Manager® and ProduXion Manager® from Aries Systems Corporation

	> * The steps involved in "approval, ethical review, and data localization" processes in each country<br>> * The key similarities and the differences between policies across countries particularly those arising from distinct "legal frameworks and cultural contexts"<br>> Providing a few illustrative comparisons (e.g., Thailand vs. Singapore vs. Korea) would make this section more concrete and more useful for readers seeking to understand cross-border interoperability challenges.<br><br>Thank you for this suggestion. We agree that the manuscript would benefit from more concrete governance examples. In the revision, we added a new comparative table (Table 2) that summarizes, side-by-side, the practical steps involved in approval/ethics review and data localization across Japan, Singapore, and Thailand, including domestic vs. international access pathways, hosting models (download/remote vs. TRE vs. secure national environment), and the key similarities and differences shaped by legal and institutional contexts. We also added brief text in the main manuscript to refer readers to this table when discussing cross-border interoperability challenges.<br><br>> #3: The manuscript mentions a series of initiatives launched at MedHackathon Asia 2025. For improved clarity, the authors could consider listing these initiatives in a table, including project description, the problem that they are trying to address, and the current state of these projects/outcomes from the Hackathon.<br><br>We thank the reviewer for this excellent suggestion. To improve clarity and help readers understand the scope and purpose of each initiative, we have added a new table (Table 3) summarizing the projects launched during MedHackathon Asia 2025, including a brief description, the problem each initiative addresses, and the current status or outcomes from the hackathon. We believe this addition makes the section more accessible and strengthens the overall manuscript.
**Additional Information:**	
**Question**	**Response**
Are you submitting this manuscript to a special series or article collection?	No
**Experimental design and statistics**<br><br>Full details of the experimental design and statistical methods used should be given in the Methods section, as detailed in our Minimum Standards Reporting Checklist. Information essential to interpreting the data presented should be made available in the figure legends.<br><br>Have you included all the information requested in your manuscript?	Yes
**Resources**<br><br>A description of all resources used, including antibodies, cell lines, animals and software tools, with enough information to allow them to be uniquely identified, should be included in the	Yes

Methods section. Authors are strongly encouraged to cite Research Resource Identifiers (RRIDs) for antibodies, model organisms and tools, where possible.<br><br>Have you included the information requested as detailed in our Minimum Standards Reporting Checklist?	
**Availability of data and materials**<br><br>All datasets and code on which the conclusions of the paper rely must be either included in your submission or deposited in publicly available repositories (where available and ethically appropriate), referencing such data using a unique identifier in the references and in the “Availability of Data and Materials” section of your manuscript.<br><br>Have you have met the above requirement as detailed in our Minimum Standards Reporting Checklist?	No
If not, please give reasons for any omissions below.<br><br>as follow-up to "**Availability of data and materials**<br><br>All datasets and code on which the conclusions of the paper rely must be either included in your submission or deposited in publicly available repositories (where available and ethically appropriate), referencing such data using a unique identifier in the references and in the “Availability of Data and Materials” section of your manuscript.<br><br>Have you have met the above requirement as detailed in our Minimum Standards Reporting Checklist?	This is a review article (white paper). We did not produce any new data. All materials are attached as supplementary files.

"	
GigaScience has policies and guidelines in place for the use of generative AI-writing tools such as ChatGPT. If you have used such writing tools to assist with writing the manuscript this must be declared and cited in the text. Authors should not list AI-writing tools and other AI-assisted technologies as an author or co-author and should acknowledge that they are fully responsible for text generated or refined by AI-writing tools.<p><br><br>A summary of use (particularly in the introduction or among methods) needs to be included at the end of the paper, and the outputs should also be included as a supplementary file hosted in GigaDB or other open repositories. Please read our guidelines for more information. <p><br><br>By submitting to GigaScience, you are aware of the journal's AI-writing tools policy, and if you have declared use of such tools below, you have acknowledged this where appropriate in your manuscript and have made a summary of use and outputs available. <p><br><b>AI-assisted writing tools have been used in the preparation of this manuscript?	No

Main text Click here to access/download;Manuscript;manuscript_final_submission.docx

Unlocking the Potential of Asian Genomic Data: A Collaborative Framework for Precision Medicine Innovation

The MedHackathon Asia Community
(The complete list of authors is provided in the supplementary file)

Abstract
Asian genomic datasets possess unparalleled potential to advance global understanding of human genetic diversity. Encompassing the world's largest population pool with diverse ethnicities, these datasets capture comprehensive genomic variations shaped by heterogeneous socioeconomic conditions, climate exposures, and clinical environments. However, current national genome initiatives across Asia demonstrate substantial disunity, stemming from limited cross-border communication and collaborative infrastructure, thereby diminishing their collective impact on biomedical research and precision medicine development. The MedHackathon Asia 2025 catalyzed crucial dialogues toward establishing a regional community dedicated to three pillars: harmonized biobank collaboration, standardized genomic data protocols, and cooperative governance frameworks. This multidisciplinary convening brought together researchers, clinicians, bioinformaticians, and national precision medicine program leaders from across Asia to share best practices, identify implementation challenges, and formulate foundational strategies for sustained cooperation. This community review synthesizes critical outcomes from these deliberations, emphasizing the imperative for continuous regional collaboration while advocating for the development of sustainable architectures enabling: (1) equitable biobank resource sharing, (2) genomic data standardization, and (3) ethical governance models. Through consolidation and expansion of this emerging network, Asian nations are expected to lead transformative contributions to global genomic science while ensuring appropriate representation in biomedical innovation. Such coordinated efforts promise to accelerate healthcare advancements with equitable benefits extending throughout the region and worldwide.

Introduction

Asia is home to over half of the world's population and harbors immense genetic, cultural, clinical, and climate diversity, presenting an extraordinary opportunity to enhance our understanding of human genetics and the application of next-generation precision medicine. With the growing number of national and international genome projects across the region, easy, open-minded, friendly, and efficient coordination and interaction among Asian researchers can amplify these benefits, facilitating the most impactful biomedical discoveries and clinical advancements in the world. There have been several notable attempts and achievements in the past to address the challenge of integrating diverse Asian populations and building shared genomic resources, such as the HUGO Pan-Asia SNP consortium [1] and the GenomeAsia 100K Project [2]. Moreover, recent advances in AI, particularly language technologies such as neural machine translation, are lowering communication barriers across Asia's diverse languages and cultures, making cross-border collaboration easier. At the same time, AI-driven analyses in genomics often benefit from large, high-quality, and ancestrally diverse datasets; pooling and harmonizing Asian genomic resources can therefore increase both scientific insight and clinical utility. Together, these trends create a strong opportunity for Asian countries to collaborate and maximize the impact of their unique genomic resources on biotechnology, public health, and patient care.

The challenges inherent in genomic data sharing in Asia span multiple, distinct layers. One major layer concerns data governance and policy: differing privacy protection laws and national data governance policies, together with uneven levels of institutional maturity and financial, technical, and human resources across countries, create substantial ethical, regulatory, and practical barriers that delay genomic data sharing and impede cross-border collaboration [3–6]. Another layer involves technical standardization. The lack of interoperable systems further complicates data sharing, as it becomes difficult to integrate datasets across jurisdictions and institutions. A third layer relates to privacy and security [7,8]. If high-profile security incidents were to occur, they could erode public trust and prompt more cautious or stricter interpretations of privacy protections and consent requirements, which in turn would further constrain cross-border genomic data sharing. Prior legal scholarship on biobank governance emphasizes that these risks should be addressed through careful governance design from the outset [9]. This includes transparent rules for information management and data access, robust controls for linkage and sharing, and a predefined incident-response process.

To overcome these challenges, we propose a region-wide approach to genomic research that prioritizes collaboration, standardization, and continuous knowledge exchange. Establishing a strong network of local researchers across Asia is essential for bridging communication gaps and ensuring that genomic research efforts align with shared goals. By fostering an interconnected research community, we can promote the adoption of common standards for data collection, storage, and sharing, thereby enhancing the impact of national genome projects.

By working together, Asian researchers can build a more inclusive and impactful genomic

research ecosystem. A well-coordinated regional approach will not only elevate the value of national efforts but also ensure that Asia contributes meaningfully to global human genetics research. The time to act is now, by fostering stronger collaborations and aligning our research frameworks, we can unlock the full potential of Asia's genomic diversity for the benefit of all.

MedHackathon Asia was initiated as a crucial step toward realizing this vision. By bringing together experts in biobanking, data analysis, and data governance, this initiative has provided a platform for researchers to discuss current challenges, share experiences, and explore strategies for harmonizing genomic research across the region. This community review summarizes the key discussions from MedHackathon Asia 2025, emphasizing the importance of a continuous and structured effort to strengthen regional collaboration. It highlights the critical need for standardizing genomic data management, addressing regulatory challenges, and establishing a sustainable model for cross-border data sharing.

This paper represents a summary of an ongoing discussion. Situations and policies within individual countries or initiatives may evolve over time; thus, the statements presented here do not represent the official position of any specific country or institution. Instead, they reflect constructive, respectful dialogue among members of our open community.

Participants of MedHackathon Asia 2025

The geographic distribution of participants in MedHackathon Asia 2025, along with bio-resources across Asia, is illustrated in **Figure 1**. Thailand, as the host of this inaugural event, had the highest number of participants, followed by Japan, with other countries contributing smaller numbers. A comprehensive inventory of existing bio-resources reveals that several countries host multiple initiatives, including national biobanks, registries, and alliances. Japan stands out with multiple national biobanks as well as alliance and registry initiatives. Thailand, Singapore, South Korea, India, Indonesia, Hong Kong SAR, Taiwan, China, and Pakistan maintain one or more biobanks, while multinational and Asia-Pacific alliances further highlight regional collaboration in biobanking and data sharing (**Table 1**, **Supplementary Table S1**). This visualization underscores the geographic reach of MedHackathon 2025 and the distribution of bio-resource infrastructure across Asia.

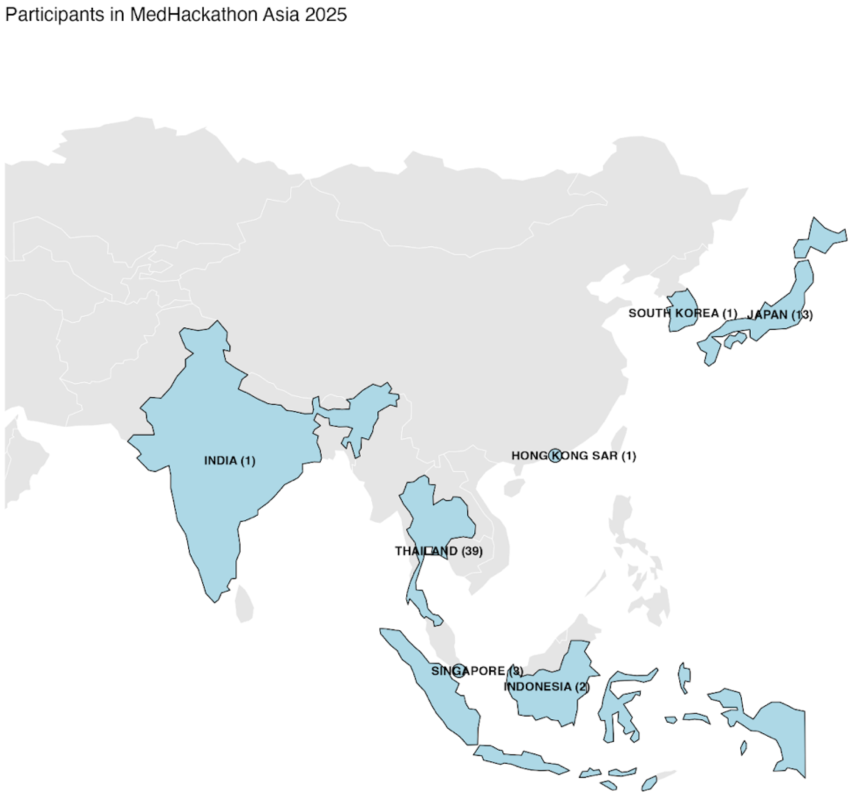

Figure 1. Distribution of participants and bio-resources in MedHackathon Asia, 2025. Countries with participants are highlighted in light blue, with the number of participants indicated in parentheses.

The Evolving Landscape of Biobanks in Asia

Biobanks play a pivotal role in biomedical research by providing high-quality biological specimens and comprehensive genomic data, which are essential for studying genetic diversity, understanding disease mechanisms, and advancing personalized medicine. Across Asia, biobank initiatives vary significantly in scale, governance, and accessibility, reflecting different national priorities and regulatory contexts. Japan has been proactive in biobank development since the early 2000s, with BioBank Japan, established in 2003, collecting samples from 270,000 participants and contributing significantly to genome-wide association studies (GWAS) through large-scale SNP array genotyping. Building upon advances in next-generation sequencing, the Tohoku Medical Megabank (TMM) project was launched in 2013 as a pioneering population-based cohort, sequencing whole genomes of 100,000 individuals to establish a foundational genomic reference in Asia. Collectively, these initiatives now form "Biobank Network Japan," storing over 1,680,000 samples from both population-based cohorts and hospital-based biobanks. Additionally, Japan's Genotype and Phenotype Archive (JGA), although not a biobank itself, serves as an essential platform enabling genomic data sharing among researchers domestically and internationally.

At a regional level, the GenomeAsia 100K Project provides an additional cross-country reference resource; its pilot phase reported a whole-genome sequencing dataset from 1,739 individuals across 219 population groups and 64 countries in Asia [2]. Singapore's National Precision Medicine Programme completed its initial phase (2017–2021) with 10,000 participants (SG10K) [10], and subsequently concluded Phase II (2021–2025) with over 100,000 genomes sequenced under the PRECISE-SG100K cohort [11]. The programme has since entered Phase III (launched 14 November 2025), which aims to scale national implementation by recruiting up to ~10% of Singapore's resident population and integrating genomics into routine healthcare delivery [12]. South Korea's Korean Genome Project [13,14] and India's GenomeIndia [15] initiative are also progressively enhancing their genomic databases, although their access models vary significantly due to different regulatory approaches. Thailand's Genomics Thailand initiative, launched in 2019, reports approximately 50,000 genomes across six groups (cancer, rare diseases, non-communicable diseases, infectious diseases, pharmacogenomics, and population genomics/ethnic groups) and emphasizes controlled access to protect data privacy and security [16–18]. In addition, Indonesia's BGSi [19] initiative, established in 2022, focuses primarily on disease-specific studies and represents an emerging contribution to the biobanking landscape in the region (**Table 1**).

In regions without representatives at MedHackathon Asia 2025, several large-scale initiatives continue to shape Asia's genomic landscape. China has made major investments in biobanking, including the China National GeneBank (CNGB), established in 2016 in Shenzhen, which serves as a national-level facility for bioresource conservation, public welfare, and life science innovation. Meanwhile, the China Kadoorie Biobank, initiated in 2004, has enrolled over

510,000 participants to investigate chronic diseases across diverse Chinese populations, and the Han100K Initiative systematically catalogs genetic variations across 100,000 Han Chinese individuals, providing a structured genomic resource for China [20]. Hong Kong launched its first large-scale whole-genome sequencing program through the Hong Kong Genome Project (HKGP), which began in 2021 with the goal of sequencing 45,000–50,000 genomes over five years, initially focusing on undiagnosed diseases and hereditary cancers to support the integration of genomics into clinical care [21]. Taiwan has also made significant strides, with the Taiwan Biobank (TWB), established in 2012, enrolling more than 150,000 participants (toward a target of 200,000) and linking genomic data to long-term national health insurance and registry records, enabling robust longitudinal studies [22]. In addition, the Taiwan Precision Medicine Initiative (TPMI), launched in 2019, is building a 1-million-person, hospital-based cohort through a network of 16 major medical centers, with over half of the target already enrolled and genotyped [23]. Pakistan also hosts an important national-scale resource, the Pakistan Genome Resource (PGR), which has been described as a biobank comprising whole-exome and whole-genome sequences of 145,037 participants, with data access handled via academic request and confidentiality agreements [24,25]. Although China, Hong Kong, and Taiwan were not represented at MedHackathon Asia 2025, their contributions to genomic research are highly valued, and we warmly welcome engagement in future collaborative efforts and regional gatherings (**Table 1**).

Table 1. Summary of Asian biobanks. (See Supplementary Table S1 for full details.)

Jurisdiction	Resource name	Participants	Sample type (healthy/disease)	Accessibility	Since
**Thailand**	Genomics Thailand	50K	Disease/Ethnicity	Aggregated data publicly available.<br><br>Controlled Local access on request.	2019
**Japan**	Tohoku Medical Megabank project (TMM)	150K	Population	Controlled	2013
**Japan**	BioBank Japan	270K	Disease	Controlled	2003
**Japan**	Biobank Network Japan	800K	Disease	Controlled	2018
**Japan**	JGA (Japanese Genotype-phenotype Archive)	422K (samples)	Population/Healthy /Disease	Controlled	2013
**Singapore**	SG10K	10K	Healthy	Managed-access through a DAC	2017
**Singapore**	PRECISE-SG100K cohort	100K	Healthy	Managed-access through a DAC	2021
**South Korea**	Korean Genome Project	10K	Population/Healthy /Disease	Partly Open	2015
**India**	GenomeIndia	≈19K	Population	Supposedly open, but policies still being drafted	2020
**Indonesia**	Biomedical and Genome Science Initiative (BGSi)	≈4K	Disease	Regulated by Indonesia’s Ministry of Health.	2022
**Multi-National**	GenomeAsia 100k	100K (to be enrolled)	Natural populations	Controlled global access on request	2016

**Hong Kong***	Hong Kong genome project (HKGP)	≈50k	Disease	Local access; global researchers by approval (de-identified data for approved studies)	2021
**Taiwan***	Taiwan Biobank (TWB)	≈200K (ongoing)	Population	Global via application (IRB approval & fee required)	2012
**Taiwan***	Taiwan Precision Medicine Initiative (TPMI)	≈500K (enrolled; aim 1M)	Various conditions	Open/Controlled approved researchers (partner hospitals & Academia Sinica)	2019
**Mainland China***	China Kadoorie Biobank (CKB)	510K	Chronic non-communicable diseases (NCDs)	Controlled global access on request (bona fide researchers apply; data delivered on approval)	2004
**Mainland China***	Han100K	100K	Natural populations	Web-based, Open	2019
**Mainland China***	Chinese Pangenome Consortium (CPC)	≈1K	Natural populations	Web-based, Open	2021
**Asia-Pacific***	Asian Pangenome Consortium (APC)	100K (to be enrolled)	Natural populations	Web-based, Open	2025
**Pakistan***	Pakistan Genome Resource	≈145K	Healthy/Disease	Controlled by CNCD	2014

Note: “*” indicates there was no official representative from that jurisdiction at MedHackathon Asia 2025.

Data Governance and Data Sharing Policies Across Biobanks

Managing genomic data governance and sample accessibility is a global challenge due to varying privacy laws, data-sharing regulations, and national policies [3]. Asian countries similarly encounter these complexities, generally following comparable approaches for domestic data sharing, typically through Data Access Committees (DACs) or equivalent approval processes, as observed in Japan, South Korea, Thailand, and Singapore [4]. However, cross-border genomic data sharing is substantially more challenging and involves additional considerations, notably personal information protection, national security and economic security concerns. Personal information protection governs privacy and data handling, facilitating easier data exchange among countries with comparable standards, such as GDPR equivalence [26]. In contrast, national economic security concerns, including the classification of genomic data as a potential strategic national resource, introduce additional oversight and restrictions on international sharing [27,28]. For example, in China, cross-border sharing of genomic data is subject to overlapping legal and administrative controls. Human genetic resource information is regulated alongside genetic materials, and provision of such information to foreign entities may require filing and, where national security or public-interest concerns arise, security review [29,30]. Recent reviews indicate that, despite ASEAN member states having developed individual regulatory arrangements, overlapping legacy frameworks and data sovereignty often impede seamless regional data sharing, underscoring the critical need for consolidated and harmonized governance frameworks [31].

Country-specific Data Sharing Policies

Asian countries generally adopt a combination of open and controlled-access policies depending on data sensitivity. While aggregated statistical data from biobanks are often openly accessible, individual-level data and biological samples typically require ethical approval from committees such as Institutional Review Boards (IRBs) or DACs. For instance, Japan, South Korea, Thailand, and Singapore follow this general model with slight differences: accessing individual-level data internationally generally requires formal research collaboration agreements with local researchers or institutions, as exemplified by Singapore's managed-access approach [7,32,33]. Similarly, Thailand's individual-level data access is currently restricted to domestic researchers, with potential for future expansion. China's genetic data policies reflect a dual commitment to fostering biomedical innovation and safeguarding national interests. While recent reforms have relaxed some controls, strict oversight, particularly for international collaborations, remains [30].

Researchers must navigate a complex landscape of approvals, ethical reviews, and data localization requirements. Despite these commonalities, understanding precise differences in each country's policies remains challenging due to varying legal frameworks and cultural contexts, further emphasizing the importance of working toward harmonized governance frameworks. These differences are highlighted when access pathways are compared across three illustrative

country examples (**Table 2**).

To illustrate how governance priorities translate into practical access pathways, **Table 2** compares the access requirements for three national genomic resources in Japan, Singapore, and Thailand. Across all three settings, individual-level genomic and linked clinical data are handled under controlled-access frameworks, typically requiring institutional ethics approval and review by a Data Access Committee (or equivalent), while summary-level outputs are generally easier to share. The main differences emerge for cross-border use and the technical model of access. In Japan, many datasets can be accessed by overseas investigators through JGA/NBDC processes, although permissions are dataset-specific and some resources are restricted to domestic use; access may be provided via encrypted download under strict security requirements or through supervised "data-visiting" arrangements (e.g., ToMMo's trusted research environment model). Singapore adopts a managed-access approach centered on a Trusted Research Environment (TRUST), where international projects typically require a Singapore-based PI and analyses are performed within the platform rather than through direct raw-data transfer. Thailand currently emphasizes domestic health-system use, with access largely limited to Thai institutions and international work commonly conducted through Thai collaborators within national secure environments; in practice, external partners typically receive approved outputs or summary results rather than individual-level data. Together, these examples show how policy and infrastructure co-evolve, through platforms to balance scientific utility with privacy, oversight, and data-sovereignty considerations.

Table 2. Comparison of Access Requirements for National Genomic Resources in Japan, Singapore and Thailand and as Illustrative Examples

Access Aspect	Japan	Singapore	Thailand
**Primary Resource**	**Tohoku Medical Megabank project, BioBank Japan, and Biobank Network Japan / NBDC Human Database / JGA** – WGS + omics + phenotype.	**PRECISE-SG100K** via **TRUST Platform** – integrated WGS + phenotype + EHR for ~100k participants.	**Genomics Thailand** – national WGS resource (target ~50,000 Thai participants) covering five priority disease areas and diverse Thai ethnic populations.
**Domestic Access**	**Permitted Controlled access and collaboration with data provider Permitted (with review)**<br>• Japanese academic /clinical and industry researchers apply to use data after approval by DAC and ethics committee.	**Permitted (via national calls)**<br>• Singapore public academic/clinical researchers apply to PRECISE calls and TRUST DAC for access to PRECISE-SG100K.	**Only Permitted (Thai institutions)**<br>• Thai investigators access Genomics Thailand data under national governance and ethics review.<br>• Data are framed as a national health resource.
**International Access**	**Currently limited**<br>• Meta-analyses have been widely conducted.<br>• The Japanese government is currently developing a national economic security framework, and it is not permitted at this time.	**Allowed (with Singapore PI)**<br>• Overseas or industry users must apply **with** a Singapore research team leading the project.<br>•Data is accessed through TRUST, not by downloading.	**Currently limited**<br>• Policies prioritise Thai researchers and domestic health-system use.<br>• International projects usually work via Thai collaborators who run the analyses inside national systems.
**Data Hosting Model**	**Trusted Research Environment (TRE) & download**<br>• **ToMMo supercomputer:** on-premises secure analytics with VDI remote access since 2015; the integrated dbTMM dataset is only accessible **inside ToMMo supercomputer** with DAC approval.<br>• **JGA / NBDC:** encrypted download of controlled-access data to approved institutional or "off-premise" servers is allowed under strict security rules.	**Trusted Research Environment (TRE)**<br>• **TRUST Platform:** cloud-based secure analytics; the integrated PRECISE-SG100K dataset is only accessible **inside TRUST** with DAC approval.<br>• Raw data are generally **not downloaded**; analysis is done in-platform.	**Secure Data Environment (SDE-style)**<br>• **Genomics Thailand** run national systems for WGS processing, variant annotation and a genome databank, with explicit "security system for data protection".<br>• Individual-level data are kept on national servers; routine download to external sites is not described in public materials.
**Procedure Paperwork & Governance**	**Medium Complexity**<br>1. Register as an authorized researcher.<br>2. Submit proposal for review by DAC after ethical approval.	**High Complexity (Managed)**<br>1. Apply via PRECISE "Call for Proposals" or collaboration route (2024–2026) with Singapore public academic/clinical PI.	**High Complexity (Restricted)**<br>1. Register as an authorized researcher within Genomics Thailand (Thai institution).<br>2. Submit proposal for review by national governance bodies.

	3. Sign an MTA/DUA / security agreement; periodic reporting expected.	2. Scientific + governance review, including TRUST DAC for EHR-linked data.<br>3. Accounts created on TRUST; outputs are checked before export.	3. Institutional verification + compliance with Thai data-protection rules; strict control on what can leave the secure environment.
**Key Systems**	• **ToMMo Supercomputer** – Trusted Research Environment.<br>• **dbTMM** – integrated database of Tohoku Medical Megabank project.<br>• **JGA** – Japanese Genotype-phenotype Archive (data archive & access).<br>• **NBDC Human Database** – policy and application portal.	• **TRUST** – Trusted Research and Real-world Utilisation and Sharing Tech; national TRE hosting integrated genomic, phenotype and clinical data from PRECISE-SG100K cohort.	• **ThaiGeR** – Thai Genome Reference Database (allele frequencies from >14k Thai genomes).<br>• **V@PP** – Variant Annotation & Prioritisation Platform for clinical/rare disease and cancer use.<br>• **Genomics Thailand Supercomputer** – national informatics and storage infrastructure.
**Possible Ideal Use Case (Illustrative)**	A collaboration that wants to **run analyses on WGS + phenotype data inside a TRE**, without moving data out, involving a Japanese-based PI.	A collaboration that wants to **run analyses on linked WGS + EHR data inside a cloud TRE**, without moving data out, led by a Singapore-based PI using the TRUST platform.	A Thai-led precision medicine project that **analyses Thai genomes in a secure national environment** (e.g. for variant interpretation or clinical filtering), with any international partners receiving **summary results, not raw data**.

Role of Digital Platforms and Infrastructure

Digital platforms play a crucial role in operationalizing data governance and ensuring secure data access and management. The Genomics Thailand initiative employs multiple digital tools, including an SMS platform for real-time updates, a Variant Annotation and Prioritization Platform (V@PP) for genomic analysis, and the Thai Genome Reference Database (ThaiGeR), along with a Secure Data Environment (SDE) featuring an "Air-lock" mechanism to restrict direct data downloads [16,18,34,35]. Additionally, the Thai Exploratory Aggregated Genome Database (ThxAD) further enhances data exploration [36]. Japan's advanced systems, such as the Tohoku University Tohoku Medical Megabank Organization (ToMMo) Supercomputer and Data Browser, provide multi-tiered access tailored to diverse research needs [37]. Singapore's TRUST platform offers a managed-access, cloud-based environment balancing data accessibility and security [38]. Meanwhile, South Korea's Korean Genome Project follows an open-access and open-source model [39], while GenomeIndia and Indonesia's BGSi utilize managed, or hybrid approaches tailored to their regulatory and cultural environments [15].

Emerging Models of Consent and Ethical Considerations

Beyond data access policies and technological solutions, informed consent mechanisms are

crucial for ethically sound and sustainable biobanking practices. Emerging models, such as dynamic consent, which allows participants to modify consent preferences in real time, have been explored as potential solutions to address limitations inherent in traditional one-time consent processes [40]. However, dynamic consent faces practical challenges, particularly regarding legacy samples, deceased donors, and long-term participant engagement [41]. As alternatives, approaches emphasizing careful regulation of data usage ("exit control") or frameworks treating health data as common goods ("data commons") are gaining attention [6,42,43]. The European Health Data Space (EHDS) and ongoing discussions on the ownership and governance of individual-level health data further underscore the importance of ensuring individual autonomy, transparency, and societal sustainability in data-sharing practices [43].

Towards Inclusive Dialogue and Regional Harmonization

Addressing these governance challenges will benefit significantly from continued and open dialogue among biobank researchers and genomic scientists across Asia. Forming collaborative communities or advisory boards to facilitate these discussions is a valuable possibility that warrants exploration. However, such efforts must respect each country's unique policies, guidelines, and cultural contexts. MedHackathon discussions emphasized that all Asian countries should have the opportunity to engage and contribute, ensuring inclusive participation without pressure towards any particular governance model. Ultimately, we believe that mutual understanding, ongoing cooperation, and collaborative development of harmonized policies will be essential for leveraging Asia's genomic diversity effectively and responsibly in biomedical research.

Balancing Data Security and Research Accessibility

A persistent challenge for biobank management is achieving a balance between robust data security and facilitating meaningful research access. Controlled-access repositories, such as Japan's NBDC Human Database and South Korea's controlled-access genome archive, employ tiered clearance systems based on data sensitivity. Data Access Committees (DACs) are essential in this context, rigorously reviewing and regulating access to ensure that both ethical and scientific standards are met [7].

Towards a Harmonized Data Governance Framework

Given the inherent challenges in cross-border data sharing, the development of an integrated governance framework is imperative. Federated data-sharing models, like those advocated by the Global Alliance for Genomics and Health (GA4GH), enable decentralized data analyses without necessitating the transfer of raw data, thereby mitigating many associated risks [44]. In tandem, standardizing metadata formats and data submission guidelines across biobanks can facilitate interoperability and streamline multi-country research initiatives [45]. Ultimately, policy

alignment among Asian nations, through the establishment of common ethical guidelines, enhanced institutional cooperation, and proactive policymaker engagement, is crucial for developing a unified framework that not only secures data but also maximizes its scientific utility. Such harmonization is essential for advancing genomic research and improving healthcare outcomes across the region.

Fostering Collaborative Genomic Innovation

Sharing knowledge and technologies in Asian genomics is emerging as a vital strategy to overcome regional fragmentation and drive collaborative progress in precision medicine. MedHackathon Asia 2025 serves as an excellent initiation of this collaborative approach by uniting diverse experts to standardize methodologies, streamline computational processes, and build shared data resources that capture the region's rich genetic diversity. The initiatives span pangenome development, federated approaches to cross-border analysis, and regional data catalogues that improve dataset discoverability and coordination, for example through federated trusted research environments and shared metadata standards. Collectively, these efforts provide practical mechanisms to strengthen sustained collaboration among Asian countries (**Table 3** and **Supplementary File S2**).

Table 3. Summary of Collaborative Initiatives and Projects Launched at MedHackathon Asia 2025

Project / Initiative	Problem or Challenge	Description & Approach	Hackathon Outcome
**Asian Pangenome Initiative**	The standard linear reference genome (GRCh38) underrepresents Asian genetic diversity, leading to bias and incomplete variant discovery.	A collaborative effort to review sequencing and assembly tools and establish a pangenome graph structure that incorporates multiple Asian genomes for better accuracy.	Formalized a multilateral partnership (APC & CPC) on April 16 to coordinate mapping efforts and harmonize ethical guidelines.
**Asian Genome-Phenome Archive (AGA) Data Catalogue**	Genomic datasets from Asia are often difficult to discover or access due to fragmentation and lack of a central registry.	Development of a regional catalogue for genomic and phenotypic datasets with standardized metadata to facilitate discovery without centralizing the data itself.	Defined a milestone-driven development strategy and a governance model where individual biobanks retain management of their respective repositories.
**Variant Analysis Pipeline Harmonization**	Inconsistent variant calling methods across countries make it difficult to compare or combine results.	Cataloging and comparing pipelines (e.g., GATK, DeepVariant) used by different Asian biobanks to develop uniform protocols for genetic interpretation.	Documented current pipelines to identify methodological divergences and assess the feasibility of region-wide standardization.
**CNV Analysis for Clinical Interpretation**	Accurate detection of Copy Number Variants (CNVs) is computationally challenging but critical for diagnosing genetic disorders.	Integration of breakpoint-based detection and coverage-depth analysis into a single pipeline to improve precision in CNV calling.	Initiated the development of a comprehensive analysis pipeline and a clinician-friendly interface to support genetic diagnosis.
**HPV DNA Detection in PBMC**	*Human Papillomavirus* (HPV) surveillance is critical for cancer prevention, but population-level data is often limited.	A bioinformatics pipeline to detect and quantify HPV DNA traces within unmapped reads of Whole Genome Sequencing (WGS) data from blood samples (PBMC).	A dedicated team is building pipelines to enable large-scale epidemiological monitoring using existing genomic datasets.
**Advancing Pharmacogenomics (PGx) & Polygenic Risk Scores (PRS)**	Precision medicine tools (PGx, PRS) developed in Western populations often lack accuracy or transferability to Asian ancestries.	Creation of Asian-specific genotyping arrays and automated systems for PGx reporting (CPIC guidelines) and PRS calculation tailored to local populations.	Established plans for automating clinical reporting and assessing the transferability of PRS models to Asian datasets.

**Federation of Trusted Research Environments (TREs)**	Data privacy laws often prevent raw data from leaving the country, hindering cross-border analysis.	Exploring a network of secure, decentralized computing environments (TREs) where analysis travels to the data rather than data traveling to researchers.	Identified common security requirements and best practices to support federated architecture.
**Federated gnomAD Aggregated Variant Browser**	Researchers need access to allele frequencies for rare disease studies, but individual-level sharing is restricted.	Integrating aggregate allele frequency data from major Asian projects (e.g., PRECISE, TMM) into a federated browser compatible with gnomAD.	Established as a low-risk "first step" for cross-border sharing that avoids transferring sensitive individual data.
**Imputation Server for Thai & Asian Genomes**	Lack of population-specific reference panels reduces the accuracy of genotype imputation for Asian populations.	Developing a standardized imputation pipeline using a Thai-specific reference panel (WGS + HLA), utilizing Common Workflow Language (CWL).	Defined the roadmap for constructing the panel, validating it, and launching a secure web-based imputation server.
**ELSI in Genomic Data Sharing**	Varying legal definitions of "personal information" and cultural trust issues create barriers to international collaboration.	Examination of Ethical, Legal, and Social Issues (ELSI) to create frameworks for equitable benefit-sharing and legal compliance across jurisdictions.	Initiated discussions to align ethical frameworks and ensure responsible data governance.

The region faces an urgent need for common data standards. The **Asian Pangenome Initiative** undertakes a detailed review of human pangenome studies across Asia by evaluating sequencing technologies, assessing genome assembly quality, and identifying methodological gaps. This effort informs the development of uniform data standards that support robust comparative research across diverse Asian populations.

At the same time, significant projects are working to harmonize computational pipelines for genomic analysis. The **Variant Analysis Pipeline Harmonization** project catalogs and compares tools used for variant calling to develop uniform protocols for genetic interpretation. In addition, the **CNV Analysis for Clinical Interpretation** project is developing a comprehensive pipeline that integrates methods for detecting copy number variants using both breakpoint detection and coverage-depth approaches. A dedicated team is also developing a pipeline for the **HPV DNA Detection in PBMC whole-genome sequencing (WGS) Data** project to detect and quantify traces of human papillomavirus DNA from whole-genome sequencing data of peripheral blood mononuclear cells. The **Imputation Pipeline/Server for Thai and Asian genome** project aligns with the imputation workflow that incorporates Japanese reference panels [32] and is implemented using common workflow language (CWL) [46]. This initiative further contributes by establishing a standardized genotype imputation framework that is tailored to the Asian and Thai population **(Figure 2)**.

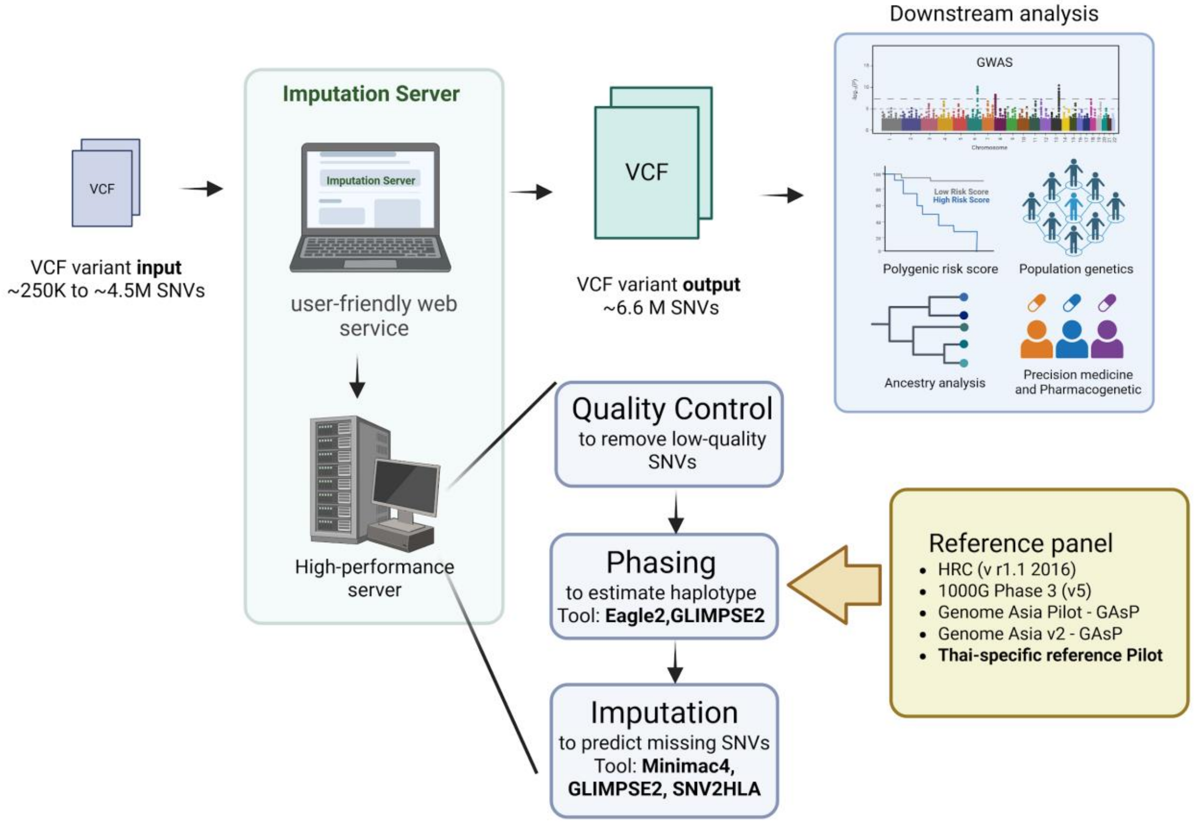

Figure 2. The concept of the imputation server with the Asian and Thai reference panel. The figure illustrates a standardized genotype imputation workflow tailored to Asian populations, incorporating high-quality reference panels such as HRC (v1.1), 1000 Genomes Project Phase 3, Genome Asia Pilot (GAsP), and a Thai-specific reference pilot. The imputation process begins with user-submitted VCF files (approximately 250K to 4.5M SNVs), processed through a user-friendly web service hosted on a high-performance computing server. Uploaded variants undergo quality control to remove low-quality SNVs, followed by haplotype phasing using tools such as Eagle2 and GLIMPSE2, and imputation to predict missing SNVs using Minimac4, GLIMPSE2, or SNV2HLA. The resulting VCF file (~6.6M SNVs) supports downstream analyses, including genome-wide association studies (GWAS), polygenic risk scoring, ancestry analyses, precision medicine, and population genetics research. This standardized approach promotes harmonized genomic analyses across Asian populations, facilitating regional collaboration and improved accuracy in genetic interpretations.

Interoperability and robust metadata management are critical to promoting cross-border collaboration. The **Asian Genome Phenome Archive** is a project that seeks to build a comprehensive regional catalogue of genomic and phenotypic datasets, complete with detailed metadata and clearly defined contact points **(Figure 3)**. Complementing this effort is the **Federated gnomAD Aggregated Variant Browser**, which aims to integrate allele frequency data from multiple Asian projects to improve data accessibility. Another initiative focuses on establishing a **Federation of Trusted Research Environments** to provide secure and decentralized platforms for data sharing while ensuring compliance with legal requirements across countries.

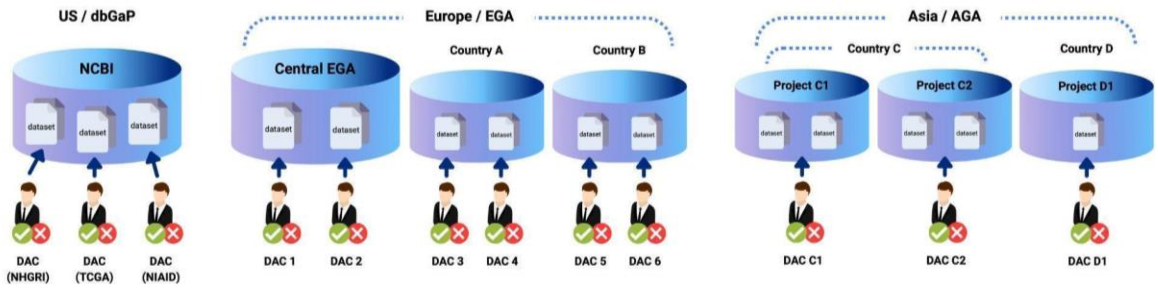

Figure 3. Comparison of data repository structures: dbGaP (USA), EGA (Europe), and the proposed Asian Genome-Phenome Archive (AGA). This figure illustrates conceptual differences in governance and data access among genomic data repositories in the United States (dbGaP), Europe (EGA), and the proposed Asian Genome-Phenome Archive (AGA). In the USA, a centralized repository is managed by the National Center for Biotechnology Information (NCBI), and Data Access Committees (DACs) from individual NIH institutes (e.g., NHGRI, TCGA, NIAID) control access to the data they fund. Europe's EGA is similarly centralized under the European Bioinformatics Institute (EBI), but individual countries may also host their own federated EGA instances, with data submitters designating which DAC controls their datasets, often forming their own committees. In contrast, the proposed AGA model in Asia suggests that each genomic research project or biobank independently manages its repository and DAC, providing permissions to all datasets within their repositories. Additionally, while dbGaP and EGA typically manage biological specimens independently from digital data (metadata, nucleotide sequences), the AGA allows biobanks themselves to archive both digital data and biological specimens in an integrated manner

Capacity building and training are essential to ensure that technological advancements are matched by a skilled workforce. The **Advancing Pharmacogenomics and Polygenic Risk Scores for Precision Medicine** project is developing a genotyping array tailored to Asian populations and automating the reporting of pharmacogenomic profiles and polygenic risk scores. This initiative not only standardizes clinical and analytical frameworks but also offers hands-on training opportunities for clinicians, bioinformaticians, and researchers. Such capacity building is crucial for integrating genomic medicine into routine clinical practice and for supporting long-term advancements in precision medicine.

MedHackathon Asia 2025 provides a dynamic platform for sharing knowledge and technologies across Asia. By facilitating collaborative projects that address common challenges and promote innovative solutions, the event lays the groundwork for a more integrated and impactful genomic research ecosystem throughout the region. (**Supplementary File S2**)

Future perspectives

The vision for genomic research in Asia depends on the successful implementation of standardized data sharing protocols and the development of an integrated data ecosystem. In a future where centralized guidelines and federated data sharing models are widely adopted, Asian institutions would follow uniform procedures for data collection, submission, and metadata annotation. Such standardization would facilitate secure, decentralized analyses while upholding local ethical and regulatory requirements (**Figure 4**). MedHackathon Asia 2025 has already initiated this collaborative process by inspiring prototype concepts, such as a unified pangenome framework and a Thai-specific imputation server, which, although still in the conceptual stage, offer promising case studies for validating these protocols.

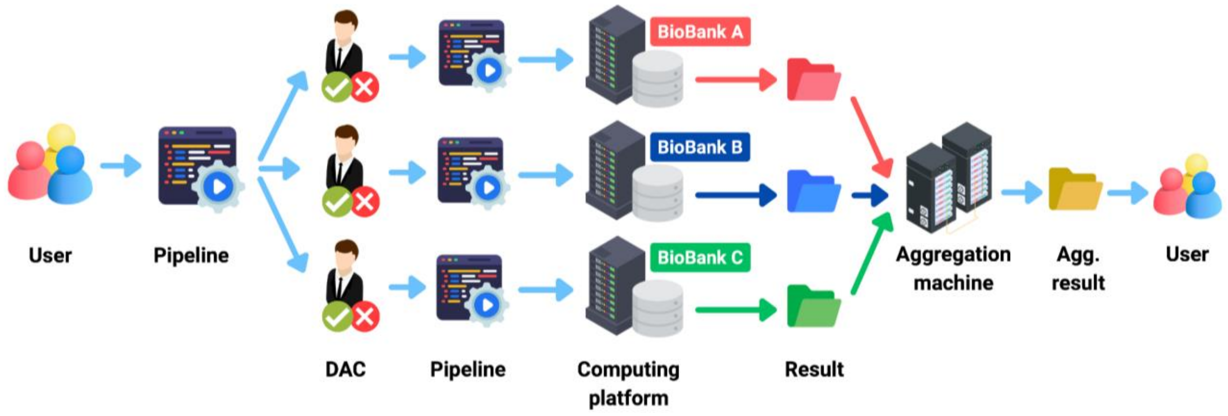

Figure 4. Proposed framework on data sharing across Asian countries. The figure outlines a conceptual framework for secure and decentralized genomic data sharing among biobanks and research institutions across Asia. Under this model, data analysis workflows or pipelines initiated by users undergo approval by each institution's Data Access Committee (DAC). Upon approval, the workflows are executed on secure computing platforms managed individually by each biobank, ensuring data privacy and regulatory compliance. Results from multiple biobanks are then aggregated securely, enabling the users to access combined analytical outcomes without direct access to raw data. This federated approach facilitates collaborative research across Asian countries while fully respecting each country's ethical guidelines, regulatory requirements, and data sovereignty.

However, to sustain the technical collaboration among institutes, it is necessary to revive or launch a new coordination hub that is bottom-up and objective-driven by participating researchers. Conferences and even Asian researcher-initiated journals will be necessary to educate the next generation of genomics researchers with a steady ecosystem of paper publication and data sharing in the region. The key problem of failing to have such an Asian control committee or organization is that the ever-changing political environment of all the nations keeps disrupting science-driven guidelines, funding, and policies. Once such a non-government-initiated, bottom-up virtual organization is established, with modest but stable support from governments across the region, it could strengthen coordination among biobanks and genome projects and support their application to other areas of biomedicine. This will enable Asian researchers to efficiently cope with international organizations such as GA4GH and even lead the biobank resources and future precision projects of the world. As AI and distributed information exchange systems such as blockchain are advancing fast, Asia's enormous body of diversified data and human resources can play a pivotal role in human health and longevity.

Looking forward, if maximum data sharing across Asian genomic initiatives with a proper research-driven coordination hub is achieved, the impact on both research and clinical practice for the world could be profound. An integrated data ecosystem that incorporates diverse genomic and phenotypic datasets would empower researchers to perform large-scale meta-analyses, uncover population-specific genetic insights, and develop innovative computational approaches to disease prediction and personalized treatment. This comprehensive data sharing would, in turn, support transformative advances in precision medicine and public health.

The potential applications of such an open data environment are multifaceted. In the field of population genetics and evolutionary studies, the vast genetic diversity of Asia, shaped by centuries of migration, adaptation, and environmental influences, could be analyzed in unprecedented detail. Researchers would be able to elucidate the genetic structure of diverse ethnic groups, trace historical migration patterns, and identify adaptations unique to specific populations. This inclusive approach would correct the current bias toward Western centric datasets and ensure that underrepresented groups contribute to and benefit from global genomic research.

Moreover, enhanced data sharing would facilitate precision medicine tailored to Asian populations. By leveraging shared data, researchers could identify genetic risk factors and develop polygenic risk scores that more accurately reflect the genetic architecture of diseases prevalent in Asia, such as hepatocellular carcinoma, nasopharyngeal cancer, and thalassemia. The pooling of data from multiple countries would also enable more effective studies of rare diseases, where limited sample sizes in individual nations have historically constrained research efforts.

In the realm of public health and epidemiology, a fully integrated open and transparent data framework will enable real-time genomic surveillance to track infectious diseases and

monitor viral evolution. By linking genomic data with clinical and epidemiological information, it would be possible to identify genetic markers associated with disease severity or vaccine response, thereby informing targeted public health interventions.

MedHackathon Asia 2025 has thus established a promising initial foundation for collaborative data sharing in the region. With the adoption of standardized protocols and the realization of a comprehensive, secure data ecosystem, the collective sharing of genomic resources can substantially accelerate scientific discovery and revolutionize precision medicine across Asia.

Data Availability

Not applicable

Competing Interests

Scott C. Edmunds was employed by GigaScience Press/BGI Group at the time of the first submission of the manuscript. Sungwon Jeon is the CEO of AgingLab and Geromics, Inc. and is employed by Clinomics, Inc. Jong Bhak is a founder of AgingLab. Shoichiro Takahashi is an employee of Trinet Corporation. Tsuyoshi Hachiya is the CEO of Genome Analytics Japan Inc. All other authors declare that they have no potential competing interests.

Funding

This work received funding from the following sources. Tazro Ohta was supported by ROIS-DS-JOINT (039RP2024) and the SECOM Science and Technology Foundation through the SECOM Challenge 2023 Research Grant. Afiahayati was supported by DCSE UGM. Ayu Kasamatsu was supported by the "Social Implementation of Infectious Disease Control Utilizing Genomic Information and Innovative Technology" project, funded by the Japan International Cooperation Agency (JICA). Warunyoo Phannasorn, Tanyaluck Kampoun, Peerut Chienwichai, Viraporn Thepbundit, and Thanapak Jaimalai were supported by the Program Management Unit for Human Resources & Institutional Development, Research and Innovation (PMU-B), under the "Researcher capability boost up for post-doctoral resource in genomic-bioinformatics for health discovery research" programme. Piyakrit Wongboonchai was supported by the Thai Society for Human Genetics (TSHG). Sunchai Payungporn was supported as the MedHackathon Asia 2025 Organizer. Apiwat Sangphukieo and Pitiporn Noisagul were supported by the Hub of Talents for Thailand Bioinformatics Research Network. Worawich Phornsiricharoenphant and Watcharapot Janpoung were supported by Genomics Thailand. Surakameth Mahasirimongkol was supported by the World Health Organization/MOPH WHO-CCS. Shuhua Xu was supported by the National Key Research and Development Program of China (No. 2023YFC2605400), the National Natural Science Foundation of China (NSFC; grant 3228801), the Shanghai Science and Technology Commission Program (25JS2810100, 23JS1410100), Fundamental and Interdisciplinary Disciplines Breakthrough Plan of the Ministry of Education of China (JYB2025XDXM508), Fund of Fudan University and Cao'ejiang Basic Research (24FCB08), and the Office of Global Partnerships (Key Projects Development Fund). Nicolas Bertin was supported by the National Research Foundation (NRF) Singapore under its National Precision Medicine Programme (NPM) Phase II

Funding (MOH-000588, awarded to Prof. Patrick Tan) and administered by the Singapore Ministry of Health's National Medical Research Council (NMRC). No specific funding was received by the other authors for this work.

Additional Files

Supplementary Table S1 — Summary of the biobanks. An overview of major biobanks and genomic resources across Asian jurisdictions, tabulating for each resource the jurisdiction, resource type, resource name, approximate number of participants, sample type (healthy/disease), sequencing platform, secure data platform, accessibility (local/global, free/requested), metadata, year of establishment, URLs and contact information, data browser, policy URL, and Data Access Committee (DAC) contacts.

Supplementary File S2 — Project descriptions. Detailed descriptions of the projects initiated during MedHackathon Asia 2025, including: the Asian Pangenome Initiative; Asian Genome-Phenome Archive (AGA) Data Catalogue; Variant Analysis Pipeline Harmonization; CNV Analysis from Paired-End Sequence for Clinical Interpretation; HPV DNA Detection in PBMC WGS Data; Advancing Pharmacogenomics (PGx) & Polygenic Risk Scores (PRS) for Precision Medicine; Federation of Trusted Research Environments (TREs); Ethical, Legal, and Social Issues (ELSI) in Genomic Data Sharing; Federated gnomAD Aggregated Variant Browser; Imputation Pipeline/Server for Thai and Asian Genomes; and Imputation Server incorporating Japanese Haplotype References.

Authors' Contributions

Conceptualization (and organization of MedHackathon): Jakris Eu-ahsunthornwattana, Tsuyoshi Hachiya, Mayumi Kamada, Toshiaki Katayama, Yosuke Kawai, Surakameth Mahasirimongkol, Pitiporn Noisagul, Soichi Ogishima, Tazro Ohta, Wasin Poncheewin, Nuttinee Teerakulkittipong, Licht Toyo-oka. Writing — original draft: Tazro Ohta, Wasin Poncheewin, Pitiporn Noisagul. Writing — review & editing: all co-authors. Figures and tables: Tazro Ohta, Wasin Poncheewin, Pitiporn Noisagul, Apiwat Sangphukieo, Shih Wee Seow, Soichi Ogishima, Nicolas Bertin, Jakris Eu-ahsunthornwattana, Tsuyoshi Hachiya, Yosuke Kawai, Minae Kawashima, Toshiaki Katayama. Supervision and senior review: Tazro Ohta, Toshiaki Katayama, Soichi Ogishima, Jong Bhak, Shuhua Xu, Mohammed S Mustak and Shih Wee Seow. All authors read and approved the final version of the manuscript.

References

1. Ngamphiw C, Assawamakin A, Xu S, Shaw PJ, Yang JO, Ghang H, et al.. PanSNPdb: The Pan-Asian SNP Genotyping Database. *PLOS ONE*. Public Library of Science; 2011; doi: 10.1371/journal.pone.0021451.

2. Wall JD, Stawiski EW, Ratan A, Kim HL, Kim C, Gupta R, et al.. The GenomeAsia 100K Project enables genetic discoveries across Asia. *Nature*. 2019; doi: 10.1038/s41586-019-1793-z.

3. Byrd JB, Greene AC, Prasad DV, Jiang X, Greene CS. Responsible, practical genomic data sharing that accelerates research. *Nature Reviews Genetics*. 2020; doi: 10.1038/s41576-020-0257-5.

4. Brunfeldt M, Teare H, Soini S, Kääriäinen H. Perceptions of legislation relating to the sharing of genomic biobank results with donors—a survey of BBMRI-ERIC biobanks. *European Journal of Human Genetics*. 2018; doi: 10.1038/s41431-017-0049-3.

5. Sanderson SC, Brothers KB, Mercaldo ND, Clayton EW, Antommaria AHM, Aufox SA, et al.. Public Attitudes toward Consent and Data Sharing in Biobank Research: A Large Multi-site Experimental Survey in the US. *The American Journal of Human Genetics*. Elsevier; 2017; doi: 10.1016/j.ajhg.2017.01.021.

6. Knoppers BM, Harris JR, Tassé AM, Budin-Ljøsne I, Kaye J, Deschênes M, et al.. Towards a data sharing Code of Conduct for international genomic research. *Genome Medicine*. 2011; doi: 10.1186/gm262.

7. Takai-Igarashi T, Kinoshita K, Nagasaki M, Ogishima S, Nakamura N, Nagase S, et al.. Security controls in an integrated Biobank to protect privacy in data sharing: rationale and study design. *BMC Medical Informatics and Decision Making*. 2017; doi: 10.1186/s12911-017-0494-5.

8. Mandl KD, Glauser T, Krantz ID, Avillach P, Bartels A, Beggs AH, et al.. The Genomics Research and Innovation Network: creating an interoperable, federated, genomics learning system. *Genetics in Medicine*. Elsevier; 2020; doi: 10.1038/s41436-019-0646-3.

9. Harmon SH, Yen S-Y, Tang S-M. Biobank governance: the cautionary tale of taiwan biobank. *SCRIPTed*. HeinOnline; 15:1032018;

10. Chan SH, Bylstra Y, Teo JX, Kuan JL, Bertin N, Gonzalez-Porta M, et al.. Analysis of clinically relevant variants from ancestrally diverse Asian genomes. *Nature Communications*. 2022; doi: 10.1038/s41467-022-34116-9.

11. Bellis C, Kolle G, Yong J, Hebrard M, Bertin N, Alexandrine Lin BC, et al.. National Scale Genomic Engine for Precision Medicine: Singapore PRECISE-SG100K Experience. *bioRxiv*. 2025; doi: 10.1101/2025.03.13.642552.

12. PRECISE — Precision Health Research, Singapore. Singapore launches Phase III of National

Precision Medicine (NPM) Programme. https://www.npm.sg/singapore-launches-phase-iii-of-national-precision-medicine-npm-programme/ (Accessed 14 April 2026)

13. An K, Jeon S, Kwon Y, Yoon C, Choi Y, Jeon Y, et al.. 10,239 whole genomes with multi-omic and clinical health information as the Korean population multi-omic reference dataset. *bioRxiv*. 2025; doi: 10.1101/2025.11.17.688763.

14. Bhak J. Korean Genome Project. https://koreangenome.org/ (Accessed 14 April 2026)

15. Bhattacharyya C, Subramanian K, Uppili B, Biswas NK, Ramdas S, Tallapaka KB, et al.. Mapping genetic diversity with the GenomeIndia project. *Nature Genetics*. 2025; doi: 10.1038/s41588-025-02153-x.

16. Shotelersuk V, Tongsima S, Pithukpakorn M, Eu-ahsunthornwattana J, Mahasirimongkol S. Precision medicine in Thailand. *American Journal of Medical Genetics Part C: Seminars in Medical Genetics*. John Wiley & Sons, Ltd; 2019; doi: 10.1002/ajmg.c.31694.

17. Genomics Thailand. Genomics Thailand Initiative home page. https://genomicsthailand.com/ (Accessed 14 April 2026)

18. Shotelersuk V, Tongsima S, Pithukpakorn M, Mahasirimongkol S, Eu-ahsunthornwattana J, Palittapongarnpim P, et al.. Advancing precision public health at Genomics Thailand. *Nature Health*. 2026; doi: 10.1038/s44360-026-00059-4.

19. Biomedical and Genome Science Initiative (BGSi), Ministry of Health of the Republic of Indonesia. BGSi home page. https://bgsi.kemkes.go.id/en (Accessed 14 April 2026)

20. Gao Y, Zhang C, Yuan L, Ling Y, Wang X, Liu C, et al.. PGG.Han: the Han Chinese genome database and analysis platform. *Nucleic Acids Research*. 2020; doi: 10.1093/nar/gkz829.

21. Annie T. W. Chu, Jasmine L. F. Fung, Amy H. Y. Tong, Sin Man Chow, Kelvin Y. K. Chan, Kit San Yeung, et al.. Potentials and challenges of launching the pilot phase of Hong Kong Genome Project. *Journal of Translational Genetics and Genomics*. 2022; doi: 10.20517/jtgg.2022.02.

22. Lin J-C, Fan C-T, Liao C-C, Chen Y-S. Taiwan Biobank: making cross-database convergence possible in the Big Data era. *GigaScience*. 2018; doi: 10.1093/gigascience/gix110.

23. Yang H-C, Kwok P-Y, Li L-H, Liu Y-M, Jong Y-J, Lee K-Y, et al.. The Taiwan Precision Medicine Initiative: A Cohort for Large-Scale Studies. *bioRxiv*. 2024; doi: 10.1101/2024.10.14.616932.

24. Riaz M, Tiller J, Ajmal M, Azam M, Qamar R, Lacaze P. Implementation of public health genomics in Pakistan. *European Journal of Human Genetics*. 2019; doi: 10.1038/s41431-019-0428-z.

25. Lamarche LB, Koch C, Khalid S, Khan MZ, Zessis R, Clement ME, et al.. Complete loss of SLC30A8 in humans improves glucose metabolism and beta cell function. *Diabetologia*. 2025;

doi: 10.1007/s00125-025-06530-3.

26. Phillips M. International data-sharing norms: from the OECD to the General Data Protection Regulation (GDPR). *Human Genetics*. 2018; doi: 10.1007/s00439-018-1919-7.

27. Berger KM, Schneck PA. National and Transnational Security Implications of Asymmetric Access to and Use of Biological Data. *Frontiers in Bioengineering and Biotechnology*. Volume 7-20192019;

28. McKibbin K, Shabani M. Genomic Data as a National Strategic Resource: Implications for the Genomic Commons and International Data Sharing for Biomedical Research and Innovation. *Journal of Law, Medicine & Ethics*. 2023/09/01 ed. Cambridge University Press; 2023; doi: 10.1017/jme.2023.77.

29. Chen Y, Song L. China: concurring regulation of cross-border genomic data sharing for statist control and individual protection. *Human Genetics*. 2018; doi: 10.1007/s00439-018-1903-2.

30. National Health Commission of the People’s Republic of China. Notice on the administration of human genetic resources. https://www.nhc.gov.cn/qjjys/rlyczygl/202503/c5373f14621e4011b6cbc932184086a2.shtml (Accessed 14 April 2026)

31. Medina PB, Armon S, Bin Abdul Aziz MF, Cheong IH, de Leon MP, Drobysz S, et al.. A Review of Regulatory Frameworks for Biobanking in Southeast Asia. *Biopreservation and Biobanking*. Mary Ann Liebert, Inc., publishers; 2025; doi: 10.1089/bio.2024.0044.

32. Hachiya T, Ishii M, Kawai Y, Khor S-S, Kawashima M, Toyo-Oka L, et al.. The NBDC-DDBJ imputation server facilitates the use of controlled access reference panel datasets in Japan. *Human Genome Variation*. 2022; doi: 10.1038/s41439-022-00225-6.

33. Precision Health Research, Singapore — PRECISE SG100K. https://www.npm.sg/data-access-principles-precise-sg100k/ (Accessed 14 April 2026).

34. Genomics Thailand. ThaiGeR — Thai Genome Reference Database. https://thaiger.genomicsthailand.com/ (Accessed 14 April 2026)

35. National Biobank of Thailand. V@PP — Variant Annotation and Prioritization Platform. https://vapp.nbt.or.th/ (Accessed 14 April 2026)

36. Genomics Thailand. ThxAD — Thai Exploratory Aggregated Genome Database. https://thxad.genomicsthailand.com/ (Accessed 14 April 2026)

37. Ogishima S, Nagaie S, Mizuno S, Ishiwata R, Iida K, Shimokawa K, et al.. dbTMM: an integrated database of large-scale cohort, genome and clinical data for the Tohoku Medical Megabank Project. *Human Genome Variation*. 2021; doi: 10.1038/s41439-021-00175-5.

38. Wong E, Bertin N, Hebrard M, Tirado-Magallanes R, Bellis C, Lim WK, et al.. The Singapore National Precision Medicine Strategy. *Nature Genetics*. 2023; doi: 10.1038/s41588-022-01274-x.

39. Jeon S, Bhak Y, Choi Y, Jeon Y, Kim S, Jang J, et al.. Korean Genome Project: 1094 Korean personal genomes with clinical information. *Science Advances*. American Association for the Advancement of Science; doi: 10.1126/sciadv.aaz7835.

40. Budin-Ljøsne I, Teare HJA, Kaye J, Beck S, Bentzen HB, Caenazzo L, et al.. Dynamic Consent: a potential solution to some of the challenges of modern biomedical research. *BMC Medical Ethics*. 2017; doi: 10.1186/s12910-016-0162-9.

41. Beskow LM, Dombeck CB, Thompson CP, Watson-Ormond JK, Weinfurt KP. Informed consent for biobanking: consensus-based guidelines for adequate comprehension. *Genetics in Medicine*. 2015; doi: 10.1038/gim.2014.102.

42. Forlini C, Hall W. A prospectus for ethical analysis of ageing individuals' responsibility to prevent cognitive decline. *Bioethics*. John Wiley & Sons, Ltd; 2017; doi: 10.1111/bioe.12387.

43. Piasecki J, Cheah PY. Ownership of individual-level health data, data sharing, and data governance. *BMC Medical Ethics*. 2022; doi: 10.1186/s12910-022-00848-y.

44. Rehm HL, Page AJH, Smith L, Adams JB, Alterovitz G, Babb LJ, et al.. GA4GH: International policies and standards for data sharing across genomic research and healthcare. *Cell Genomics*. 2021; doi: 10.1016/j.xgen.2021.100029.

45. Jacobsen JOB, Baudis M, Baynam GS, Beckmann JS, Beltran S, Buske OJ, et al.. The GA4GH Phenopacket schema defines a computable representation of clinical data. *Nature Biotechnology*. 2022; doi: 10.1038/s41587-022-01357-4.

46. Crusoe MR, Abeln S, Iosup A, Amstutz P, Chilton J, Tijanić N, et al.. Methods included: standardizing computational reuse and portability with the Common Workflow Language. *Commun ACM*. New York, NY, USA: Association for Computing Machinery; 2022; doi: 10.1145/3486897.

Figure4

Click here to access/download;Figure;Figure4.png

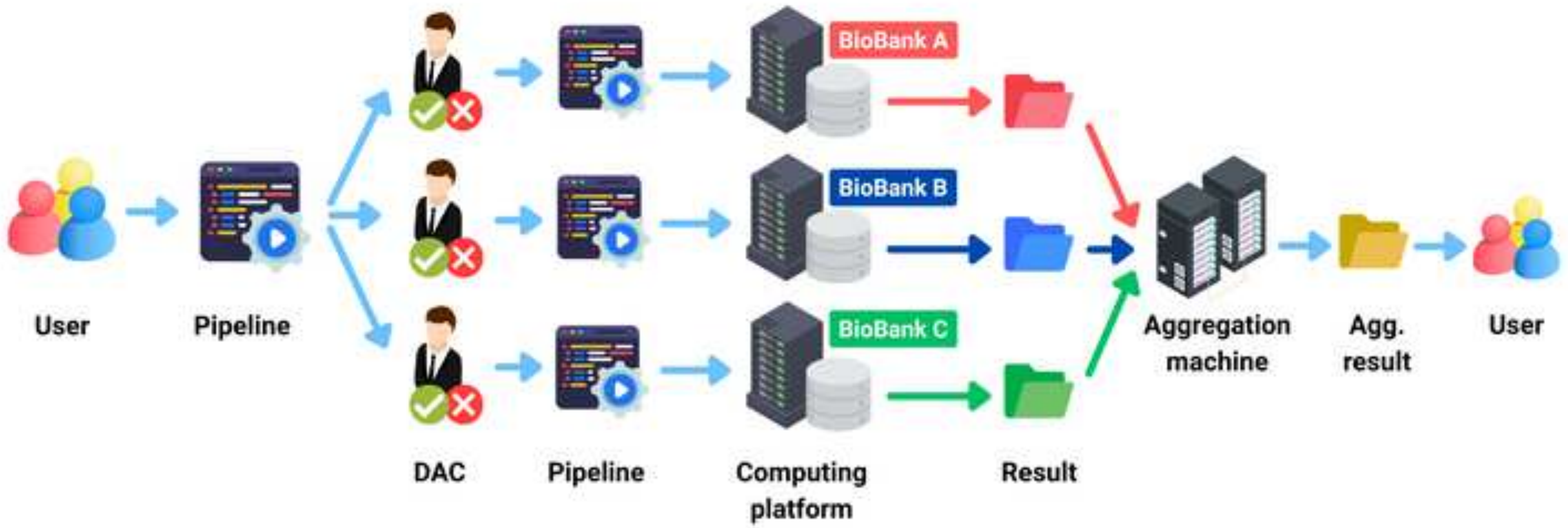

Figure1

Click here to access/download;Figure;Figure1.png

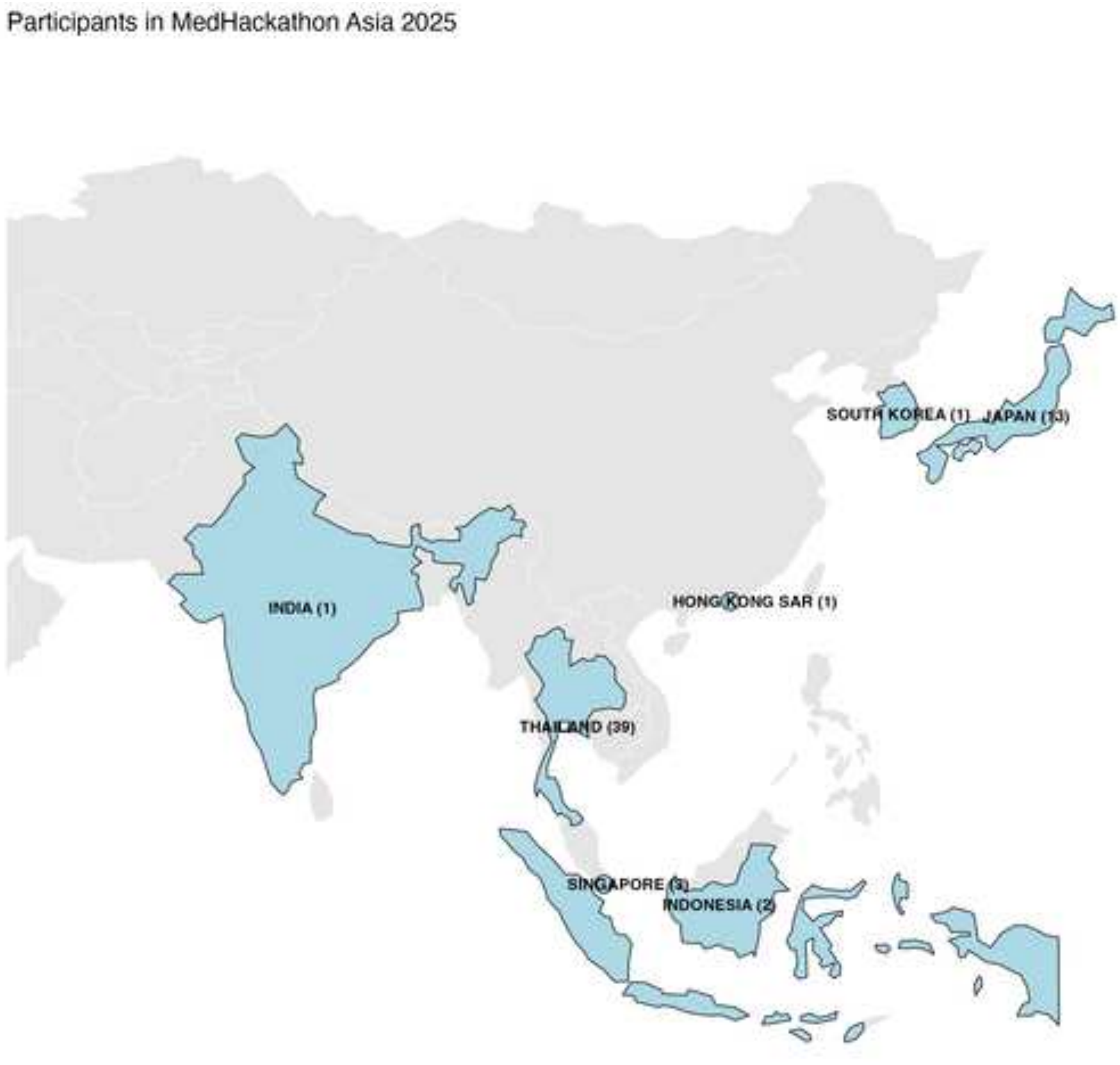

Figure2

Click here to access/download;Figure;Figure2.png

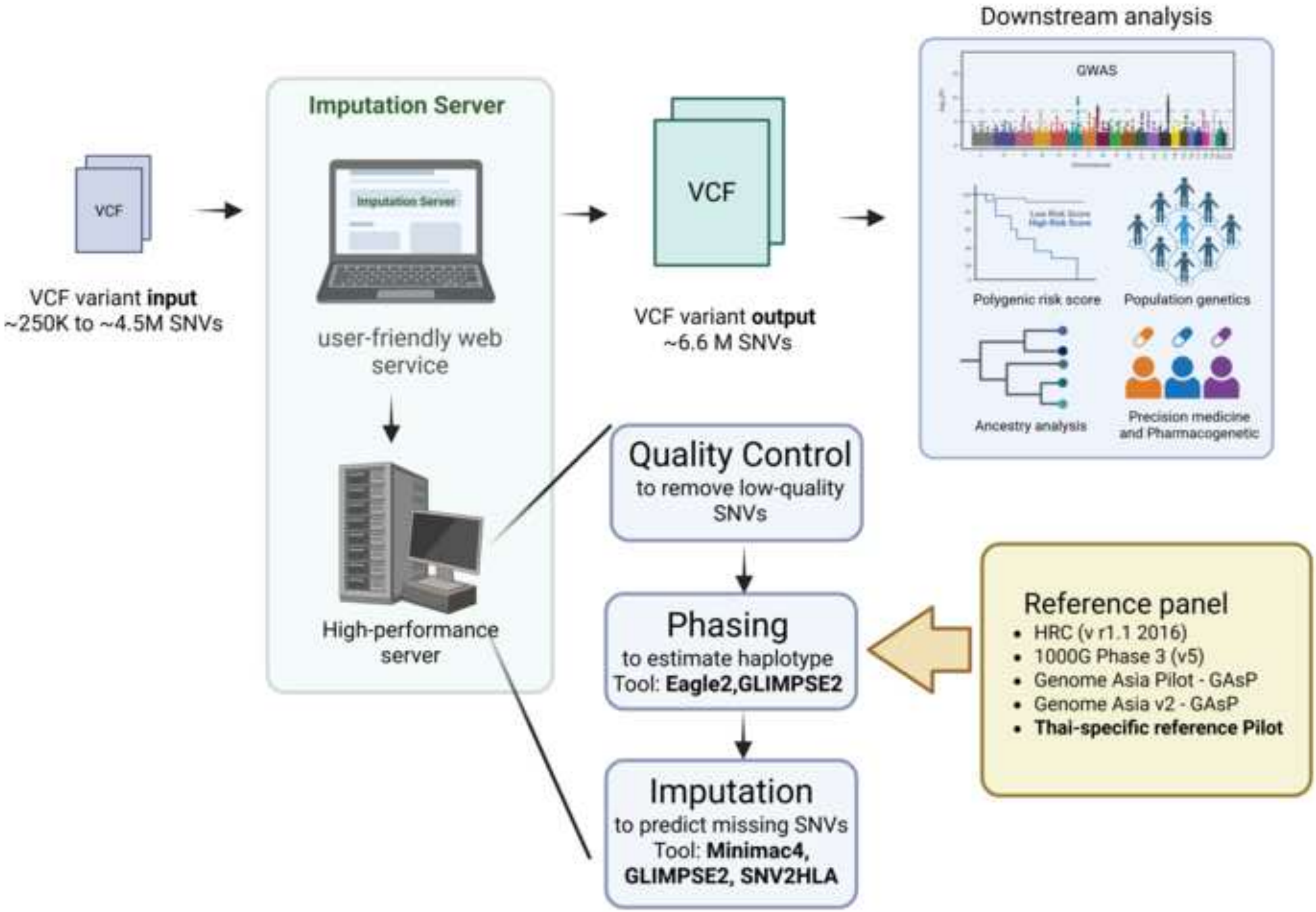

Figure3

Click here to access/download;Figure;Figure3.png

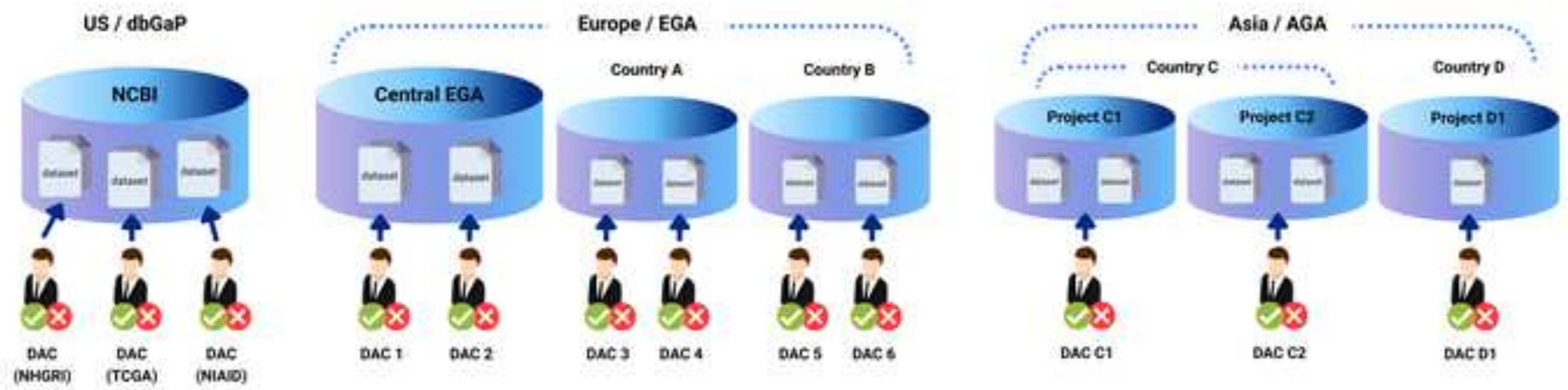

Supplementary Table S1

Click here to access/download
Supplementary Material
Supplementary Table S1 - summary of the biobanks.xlsx

Supplementary File S2

Click here to access/download

Supplementary Material

Supplementary File S2 - project descriptions.docx

Author_list

Click here to access/download
Supplementary Material
Author_list.xlsx
