# Supplementary material for: Unlocking the potential of Asian genomic data: a collaborative framework for precision medicine innovation
Source: Gigascience. 2026 May 5;15:giag052. doi: 10.1093/gigascience/giag052 (PMC13196594; doi:10.1093/gigascience/giag052)
Supplement: giag052_Authors_Response_To_Reviewer_Comments_original_submission [file giag052_authors_response_to_reviewer_comments_original_submission.pdf]

Author's Response To Reviewer Comments

Response to Reviewer #1

We sincerely thank the reviewer for the thoughtful and supportive assessment of our manuscript. We are grateful that you found the topic timely and relevant, and we appreciate your recognition of the collective expertise represented in the MedHackathon Asia community.

We acknowledge the reviewer’s concern regarding the difficulty in distinguishing which statements are grounded in existing academic literature and policy, and which represent recommendations or perspectives developed through discussions at MedHackathon Asia 2025. In response, we have added missing citations to attribute statements that draw on existing research or online resources. We have also refined the language in sections containing recommendations or viewpoints from the hackathon discussions to ensure these contributions are clearly distinguished from literature-based descriptions.

We believe these revisions significantly improve clarity for the reader and better highlight the contributions of both the academic literature and the hackathon community. We thank the reviewer again for helping us strengthen the manuscript.

> ## Abstract
> I think the term "Whitepaper" is used in many different ways, often it applies to not-peer-reviewed papers too. I would suggest replacing this with a more appropriate term.

We thank the reviewer for this suggestion. We agree that the term “whitepaper” may be ambiguous and have replaced it with “community review” to more accurately reflect the nature of this manuscript.

> ## Intro
> "in the era of artificial intelligence (AI), which is dramatically accelerating communication among Asian countries with diverse languages and cultures, there is great potential for collaboration." -> I'm not 100% sure I follow the argument, is this about machine translation that enables communication between those countries? It would be nice to clarify this.

Our intention was not only to refer to AI-enabled tools such as machine translation that can facilitate communication across linguistic and cultural boundaries, but also to highlight that the era of AI increases the value of high-quality, diverse datasets. This creates new opportunities for Asian countries to collaborate and collectively maximize the impact of their unique genomic and health resources. We have revised the first paragraph of the Introduction section to clarify this point and make the connection between AI and collaborative potential more explicit.

> The example of the data breach in Taiwan seems a bit out of place: In the sentences before, the authors highlight the challenges of privacy laws & consent structures, as well as lack of interoperability. But I'm not sure how a data breach demonstrates something across those factors? It would be good to embed this better/expand the argument appropriately.

We agree that the previous phrasing did not clearly connect the “breach” example to the surrounding discussion of privacy/consent requirements and interoperability. In revising this section, we also realized that our earlier mention of a “Taiwan Biobank data breach” was not supported by an official confirmation and could therefore be misleading. We have removed this unconfirmed example to avoid an unintended or inaccurate argument, and revised the section to more clearly emphasize the broader challenges biobanks face in cross-border collaboration. We sincerely thank the reviewer for raising this point and helping us correct the manuscript.

> It would be good to get some demographics/statistics of the hackathon: how many people and from which regions/countries did participate in the event and later write-up? Later on it's mentioned that there weren't participants from HK/China/TW, but readers don't get an idea of who was in the room

To provide clearer context on the perspectives represented, we have added a short paragraph and new Figure 1 summarizing the number of participants and the countries and regions involved in both the MedHackathon Asia 2025 event and the subsequent manuscript development. We agree that this information helps readers understand who contributed to the discussions and clarifies the geographic scope of the community.

> ## Biobanks
> The current structure on biobanks (section "The Evolving Landscape of Biobanks in Asia") flows a bit strangely, I presume all mentioned countries except for HK/China/Taiwan participated in the Hackathon? If that's the case it feels a bit strange to break the flow to go from the participating countries into a large block of non-participating countries, before heading back to particpating ones.

We agree that the current structure may feel uneven, as it shifts between countries that participated in the hackathon and those that did not. To improve readability and clarity, we have revised the section to first summarize the biobanking landscape based on the experiences shared by participating countries, followed by a separate paragraph describing major initiatives in regions without representation at the event (e.g., China, Hong Kong, Taiwan). This restructuring provides a more coherent narrative while ensuring comprehensive regional coverage.

> While the sections on Japan & China are well-sourced, it would be good if the authors could also include references to the other biobanks and efforts, in particular for Singapore, South Korea, India and Indonesia
We have added appropriate references for these resources in the revised manuscript.

> The section on "Data Governance and Data Sharing Policies Across Biobanks" lacks citations, with only one given so far. I appreciate that there would be lots of different regulations that could apply across nations, but it would be good to at least give a some concrete examples (e.g. which countries apply national security concerns to genetic data?)

We agree that the section would benefit from more concrete, well-sourced examples. In the revision, we added additional references to national data governance frameworks and published discussions of genomic data governance and national-security considerations where authoritative sources are available. We also added a comparative table (Japan, Singapore, and Thailand) that summarizes key governance and access features (e.g., domestic vs. international access, hosting/localization models, and approval/ethics review processes) to make the policy differences more explicit and actionable for readers.

> The same lack of sources applies to the country-specific data sharing policies section and the role of digital platforms/infrastructure sections. Many systems are called out in the latter section in particular, but none are given citations.

We thank the reviewer for highlighting the need for additional sourcing in these sections. In the revised manuscript, we have added references for the data-sharing policies and digital platforms mentioned, including official documentation, published descriptions, or institutional websites when peer-reviewed sources are not available.

> The "Emerging models of consent" section mentions "approaches emphasizing careful regulation of data usage" that are gaining attention. By whom do they gain attention and is this the case in Asia or generally? A reference would be appreciated here too

The intention of this section was to reflect a trend observed both in Asia and internationally, based on discussions among MedHackathon participants as well as broader developments in biobank ethics. To avoid ambiguity, we have revised the text to clarify that these approaches, such as strengthened data-use regulation (“exit control”) and data commons models, are gaining attention in global discussions on biobank governance. We have also added appropriate citations to support this statement.

> "Balancing Data Security and Research Accessibility": Are those the opinions of the hackathon attendees or generally accepted, citeable facts?

The statements in this section reflect both the perspectives shared by MedHackathon attendees and widely recognized challenges discussed in the biobanking and data governance literature. To avoid ambiguity, we have revised the text to clarify this and have added citations where suitable sources are available.

> Towards a harmonized data governance framework: please reference GA4GH's work you mention. Otherwise: Same as above, are the rest of the statements claims made by the attendees/authors or based on existing work?

We have added citations to the relevant Global Alliance for Genomics and Health (GA4GH) frameworks mentioned in this section.

> ## Fostering Collaborative Genomic Innovation
> Are the different, bolded "teams" or "projects" in this section teams that formed during the hackathon? If so that's not fully clear and should be expanded upon, otherwise please reference the external projects

The bolded teams and projects in this section represent collaborative groups that formed during MedHackathon Asia 2025, rather than pre-existing external initiatives. To make this clearer for readers, we added Table 3 ("Summary of Collaborative Initiatives and Projects Launched at MedHackathon Asia 2025"), which lists these teams/projects and summarizes their scope and outputs.

Response to Reviewer #2

> Overall Assessment
> This whitepaper provides a timely and thoughtful overview of the rapidly evolving genomics landscape in Asia. It brings together a wide range of national initiatives, describes the governance challenges and opportunities for regional collaboration in Asian genomics, and highlights the technical efforts that emerged from MedHackathon Asia 2025. This topic is timely and important: the underrepresentation of Asian genomics is a major goal gap, and consolidated regional infrastructure and collaboration can significantly improve precision medicine for a large portion of the world's population.
> Overall, this whitepaper lays a strong foundation for future collaborative efforts in Asian genomics and will be of strong interest to readers working on large-scale biobanks, international data governance, and precision medicine implementation.

We sincerely thank the reviewer for the thoughtful and encouraging assessment of our manuscript. We greatly appreciate your recognition of the importance of strengthening regional collaboration in Asian genomics and your acknowledgement that this work lays a meaningful foundation for future initiatives. Your positive feedback on the relevance and timeliness of this paper is highly motivating for our community. We thank you again for your constructive comments and for supporting the advancement of collaborative genomic efforts across Asia.

> #1: In Supplementary Table 1, the authors provide a comprehensive summary of biobanks across Asia. To enhance its comprehensiveness, the authors may wish to incorporate a few additional regional initiatives, including:
> ChinaHEART: https://academic.oup.com/ije/article/52/5/e273/7187439
> GenomeAsia 100k: https://www.nature.com/articles/s41586-019-1793-z
> Pakistan Genome Resource: https://www.nature.com/articles/nature22034

We thank the reviewer for these valuable suggestions. We have incorporated GenomeAsia 100K and the Pakistan Genome Resource into Table1 and Supplementary Table 1 as recommended.
Regarding ChinaHEART, after reviewing the cited publication, we found that the initiative primarily focuses on large-scale cardiovascular epidemiology and clinical data collection, without establishing a biobank for biological specimens or generating genomic datasets. As our table specifically summarizes biobanks and genomic resources, we believe ChinaHEART does not fit the scope of this section. Therefore, we have not included it in the revised table.

> #2: The manuscript identifies governance challenges well, but could benefit from a slightly deeper discussion on policy and governance examples. For instance, in page 10, the authors note:

> "Researchers must navigate a complex landscape of approvals, ethical reviews, and data localization requirements. Despite these commonalities, understanding precise differences in each country's policies remains challenging due to varying legal frameworks and cultural contexts, further emphasizing the importance of working toward harmonized governance frameworks."
> While Supplementary Table 1 summarizes platforms and access policies across biobanks, the manuscript would be strengthened by providing more detail on:
> * The steps involved in "approval, ethical review, and data localization" processes in each country
> * The key similarities and the differences between policies across countries particularly those arising from distinct "legal frameworks and cultural contexts"
> Providing a few illustrative comparisons (e.g., Thailand vs. Singapore vs. Korea) would make this section more concrete and more useful for readers seeking to understand cross-border interoperability challenges.

Thank you for this suggestion. We agree that the manuscript would benefit from more concrete governance examples. In the revision, we added a new comparative table (Table 2) that summarizes, side-by-side, the practical steps involved in approval/ethics review and data localization across Japan, Singapore, and Thailand, including domestic vs. international access pathways, hosting models (download/remote vs. TRE vs. secure national environment), and the key similarities and differences shaped by legal and institutional contexts. We also added brief text in the main manuscript to refer readers to this table when discussing cross-border interoperability challenges.

> #3: The manuscript mentions a series of initiatives launched at MedHackathon Asia 2025. For improved clarity, the authors could consider listing these initiatives in a table, including project description, the problem that they are trying to address, and the current state of these projects/outcomes from the Hackathon.

We thank the reviewer for this excellent suggestion. To improve clarity and help readers understand the scope and purpose of each initiative, we have added a new table (Table 3) summarizing the projects launched during MedHackathon Asia 2025, including a brief description, the problem each initiative addresses, and the current status or outcomes from the hackathon. We believe this addition makes the section more accessible and strengthens the overall manuscript.

em Editorial Manager®

Cookie Preference Center

We use cookies which are necessary to make our site work. We may also use additional cookies to analyze, improve and personalize our content and your digital experience. For more information, see our Cookie Policy.

You may choose not to allow some types of cookies. However, blocking some types may impact your experience of our site and the services we are able to offer. See the different category headings below to find out more or change your settings.

Allow all

Manage Consent Preferences

Strictly Necessary Cookies

Always active

These cookies are necessary for the website to function and cannot be switched off in our systems. They are usually only set in response to actions made by you which amount to a request for services, such as setting your privacy preferences, logging in or filling in forms. You can set your browser to block or alert you about these cookies, but some parts of the site will not then work.

Functional Cookies

These cookies enable the website to provide enhanced functionality and personalisation. They may be set by us or by third party providers whose services we have added to our pages. If you do not allow these cookies then some or all of these services may not function properly.

Performance Cookies

These cookies allow us to count visits and traffic sources so we can measure and improve the performance of our site. They help us to know which pages are the most and least popular and see how visitors move around the site.

Targeting Cookies

These cookies may be set through our site by our advertising partners. They may be used by those companies to build a profile of your interests and show you relevant adverts on other sites. If you do not allow these cookies, you will experience less targeted advertising.

Cookie List

Clear

Apply Cancel

Consent Leg.Interest

Confirm my choices

Power
