# Supplementary material for: Unlocking the potential of Asian genomic data: a collaborative framework for precision medicine innovation
Source: Gigascience. 2026 May 5;15:giag052. doi: 10.1093/gigascience/giag052 (PMC13196594; doi:10.1093/gigascience/giag052)
Supplement: giag052_Reviewer_1_Report_original_submission — Reviewer 1 -- 11/19/2025 [file giag052_reviewer_1_report_original_submission.pdf]

**Reviewer Report**

**Title: Unlocking the Potential of Asian Genomic Data: A Collaborative Framework for Precision Medicine Innovation**

**Version: Original Submission** **Date:** 11/19/2025

**Reviewer name: Reviewer 1**

**Reviewer Comments to Author:**

The authors present a paper that is the outcome of a collaborative dialogue held at the 2025 MedHackathon Asia, to foster improvements in collaboration and communication around genomic data and biobanks, to support biomedical research and precision medicine. In particular, they advocate for better resource, data and infrastructure sharing under ethical governance principles.
I think that this is a very relevant and timely contribution, and I have no doubt that the authors have a lot of expertise and experience to make robust recommendations.
My only/main issue with the manuscript as it currently stands, is that - at times - it is very hard to understand as a reader, which parts of the different recommendations and claims are based in the academic literature & or existing policy and which ones were developed by the authors during the hackathon based on the discussions. I think this is due to two factors:
1. Many claims across the different sections that seemingly are based on external factors do not have references to the sources of those claims.
2. Where recommendations are (likely) made by the authors, there are no clear indications that this is the case. By slightly adjusting the language, e.g. adding "we think", "we recommend", "should/will/would" etc., it would help see the contributions of the hackathon community
By adding in the missing references - and where appropriate clarifying the language - this confusion could be resolved quite easily I think.
I've tried to leave some detailed feedback on the different sections below, which mostly is about these bits:
## Abstract
- I think the term "Whitepaper" is used in many different ways, often it applies to not-peer-reviewed papers too. I would suggest replacing this with a more appropriate term.
## Intro
- "in the era of artificial intelligence (AI), which is dramatically accelerating communication among Asian countries with diverse languages and cultures, there is great
potential for collaboration." -> I'm not 100% sure I follow the argument, is this about machine translation that enables communication between those countries? It would be nice to clarify this.
- The example of the data breach in Taiwan seems a bit out of place: In the sentences before, the authors highlight the challenges of privacy laws & consent structures, as well as lack of interoperability. But I'm not sure how a data breach demonstrates something across those factors? It would be good to embed this better/expand the argument appropriately.
- It would be good to get some demographics/statistics of the hackathon: how many people and from

which regions/countries did participate in the event and later write-up? Later on it's mentioned that there weren't participants from HK/China/TW, but readers don't get an idea of who was in the room
## Biobanks
- The current structure on biobanks (section "The Evolving Landscape of Biobanks in Asia") flows a bit strangely, I presume all mentioned countries except for HK/China/Taiwan participated in the Hackathon? If that's the case it feels a bit strange to break the flow to go from the participating countries into a large block of non-participating countries, before heading back to particpating ones.
- While the sections on Japan & China are well-sourced, it would be good if the authors could also include references to the other biobanks and efforts, in particular for Singapore, South Korea, India and Indonesia
- The section on "Data Governance and Data Sharing Policies Across Biobanks" lacks citations, with only one given so far. I appreciate that there would be lots of different regulations that could apply across nations, but it would be good to at least give a some concrete examples (e.g. which countries apply national security concerns to genetic data?)
- The same lack of sources applies to the country-specific data sharing policies section and the role of digital platforms/infrastructure sections. Many systems are called out in the latter section in particular, but none are given citations.
- The "Emerging models of consent" section mentions "approaches emphasizing careful regulation of data usage" that are gaining attention. By whom do they gain attention and is this the case in Asia or generally? A reference would be appreciated here too
- "Balancing Data Security and Research Accessibility": Are those the opinions of the hackathon attendees or generally accepted, citeable facts?
- Towards a harmonized data governance framework: please reference GA4GH's work you mention. Otherwise: Same as above, are the rest of the statements claims made by the attendees/authors or based on existing work?
## Fostering Collaborative Genomic Innovation
- Are the different, bolded "teams" or "projects" in this section teams that formed during the hackathon? If so that's not fully clear and should be expanded upon, otherwise please reference the external projects

**Methods**

Are the methods appropriate to the aims of the study, are they well described, and are necessary controls included? Choose an item.

**Conclusions**

Are the conclusions adequately supported by the data shown? Choose an item.

**Reporting Standards**

Does the manuscript adhere to the journal's guidelines on minimum standards of reporting? Choose an item.

Choose an item.

**Statistics**

Are you able to assess all statistics in the manuscript, including the appropriateness of statistical tests used? Choose an item.

**Quality of Written English**

Please indicate the quality of language in the manuscript: Choose an item.

**Declaration of Competing Interests**

Please complete a declaration of competing interests, considering the following questions:

- Have you in the past five years received reimbursements, fees, funding, or salary from an organisation that may in any way gain or lose financially from the publication of this manuscript, either now or in the future?
- Do you hold any stocks or shares in an organisation that may in any way gain or lose financially from the publication of this manuscript, either now or in the future?
- Do you hold or are you currently applying for any patents relating to the content of the manuscript?
- Have you received reimbursements, fees, funding, or salary from an organization that holds or has applied for patents relating to the content of the manuscript?
- Do you have any other financial competing interests?
- Do you have any non-financial competing interests in relation to this paper?

If you can answer no to all of the above, write 'I declare that I have no competing interests' below. If your reply is yes to any, please give details below.

I declare that I have no competing interests

I agree to the open peer review policy of the journal. I understand that my name will be included on my report to the authors and, if the manuscript is accepted for publication, my named report including any attachments I upload will be posted on the website along with the authors' responses. I agree for my report to be made available under an Open Access Creative Commons CC-BY license (http://creativecommons.org/licenses/by/4.0/). I understand that any comments which I do not wish to be included in my named report can be included as confidential comments to the editors, which will not be published.

Choose an item.

To further support our reviewers, we have joined with Publons, where you can gain additional credit to further highlight your hard work (see: https://publons.com/journal/530/gigascience). On publication of this paper, your review will be automatically added to Publons, you can then choose whether or not to claim your Publons credit. I understand this statement.

Yes Choose an item.
