# Supplementary material for: Unlocking the potential of Asian genomic data: a collaborative framework for precision medicine innovation
Source: Gigascience. 2026 May 5;15:giag052. doi: 10.1093/gigascience/giag052 (PMC13196594; doi:10.1093/gigascience/giag052)
Supplement: giag052_Reviewer_2_Report_original_submission — Reviewer 2 -- 12/4/2025 [file giag052_reviewer_2_report_original_submission.pdf]

Reviewer Report

Title: Unlocking the Potential of Asian Genomic Data: A Collaborative Framework for Precision Medicine Innovation

Version: Original Submission **Date:** 12/4/2025

Reviewer name: Reviewer 2

Reviewer Comments to Author:

Manuscript ID: GIGA-D-25-00441
Title: Unlocking the Potential of Asian Genomic Data: A Collaborative Framework for Precision Medicine Innovation
Overall Assessment
This whitepaper provides a timely and thoughtful overview of the rapidly evolving genomics landscape in Asia. It brings together a wide range of national initiatives, describes the governance challenges and opportunities for regional collaboration in Asian genomics, and highlights the technical efforts that emerged from MedHackathon Asia 2025. This topic is timely and important: the underrepresentation of Asian genomics is a major goal gap, and consolidated regional infrastructure and collaboration can significantly improve precision medicine for a large portion of the world's population.
Overall, this whitepaper lays a strong foundation for future collaborative efforts in Asian genomics and will be of strong interest to readers working on large-scale biobanks, international data governance, and precision medicine implementation.
Here I offer some constructive suggestions aimed at strengthening the clarity and flow of the manuscript:
#1: In Supplementary Table 1, the authors provide a comprehensive summary of biobanks across Asia. To enhance its comprehensiveness, the authors may wish to incorporate a few additional regional initiatives, including:
ChinaHEART: https://academic.oup.com/ije/article/52/5/e273/7187439
GenomeAsia 100k: https://www.nature.com/articles/s41586-019-1793-z
Pakistan Genome Resource: https://www.nature.com/articles/nature22034
#2: The manuscript identifies governance challenges well, but could benefit from a slightly deeper discussion on policy and governance examples. For instance, in page 10, the authors note:
"Researchers must navigate a complex landscape of approvals, ethical reviews, and data localization requirements. Despite these commonalities, understanding precise differences in each country's policies remains challenging due to varying legal frameworks and cultural contexts, further emphasizing the importance of working toward harmonized governance frameworks."
While Supplementary Table 1 summarizes platforms and access policies across biobanks, the manuscript would be strengthened by providing more detail on:
* The steps involved in "approval, ethical review, and data localization" processes in each country
* The key similarities and the differences between policies across countries particularly those arising from distinct "legal frameworks and cultural contexts"

Providing a few illustrative comparisons (e.g., Thailand vs. Singapore vs. Korea) would make this section more concrete and more useful for readers seeking to understand cross-border interoperability challenges.
#3: The manuscript mentions a series of initiatives launched at MedHackathon Asia 2025. For improved clarity, the authors could consider listing these initiatives in a table, including project description, the problem that they are trying to address, and the current state of these projects/outcomes from the Hackathon.

Methods

Are the methods appropriate to the aims of the study, are they well described, and are necessary controls included? Choose an item.

Conclusions

Are the conclusions adequately supported by the data shown? Choose an item.

Reporting Standards

Does the manuscript adhere to the journal's guidelines on minimum standards of reporting? Choose an item.

Choose an item.

Statistics

Are you able to assess all statistics in the manuscript, including the appropriateness of statistical tests used? Choose an item.

Quality of Written English

Please indicate the quality of language in the manuscript: Choose an item.

Declaration of Competing Interests

Please complete a declaration of competing interests, considering the following questions:

- Have you in the past five years received reimbursements, fees, funding, or salary from an organisation that may in any way gain or lose financially from the publication of this manuscript, either now or in the future?
- Do you hold any stocks or shares in an organisation that may in any way gain or lose financially from the publication of this manuscript, either now or in the future?
- Do you hold or are you currently applying for any patents relating to the content of the manuscript?

- Have you received reimbursements, fees, funding, or salary from an organization that holds or has applied for patents relating to the content of the manuscript?
- Do you have any other financial competing interests?
- Do you have any non-financial competing interests in relation to this paper?

If you can answer no to all of the above, write 'I declare that I have no competing interests' below. If your reply is yes to any, please give details below.

I declare that I have no competing interests

I agree to the open peer review policy of the journal. I understand that my name will be included on my report to the authors and, if the manuscript is accepted for publication, my named report including any attachments I upload will be posted on the website along with the authors' responses. I agree for my report to be made available under an Open Access Creative Commons CC-BY license (http://creativecommons.org/licenses/by/4.0/). I understand that any comments which I do not wish to be included in my named report can be included as confidential comments to the editors, which will not be published.

Choose an item.

To further support our reviewers, we have joined with Publons, where you can gain additional credit to further highlight your hard work (see: https://publons.com/journal/530/gigascience). On publication of this paper, your review will be automatically added to Publons, you can then choose whether or not to claim your Publons credit. I understand this statement.

Yes Choose an item.
